# Correction: Lu et al. Autophagy Alters Bladder Angiogenesis and Improves Bladder Hyperactivity in the Pathogenesis of Ketamine-Induced Cystitis in a Rat Model. *Biology* 2021, *10*, 488

**DOI:** 10.3390/biology14111630

**Published:** 2025-11-20

**Authors:** Jian-He Lu, Yi-Hsuan Wu, Tai-Jui Juan, Hung-Yu Lin, Rong-Jyh Lin, Kuang-Shun Chueh, Yi-Chen Lee, Chao-Yuan Chang, Yung-Shun Juan

**Affiliations:** 1 Emerging Compounds Research Center, Department of Environmental Science and Engineering, College of Engineering, National Pingtung University of Science and Technology, Pingtung 91201, Taiwan; toddherpuma@yahoo.com.tw; 2 Department of Urology, College of Medicine, Kaohsiung Medical University, Kaohsiung 80708, Taiwan; maivy0314@gmail.com; 3 Department of Urology, Kaohsiung Medical University Hospital, Kaohsiung 80756, Taiwan; spacejason69@yahoo.com.tw; 4 Graduate Institute of Clinical Medicine, College of Medicine, Kaohsiung Medical University, Kaohsiung 80708, Taiwan; 5 Department of Medicine, National Defense Medical College, Taipei 11490, Taiwan; u9181002@gmail.com; 6 School of Medicine, College of Medicine, I-Shou University, Kaohsiung 84001, Taiwan; ed100464@edah.org.tw; 7 Division of Urology, Department of Surgery, E-Da Cancer Hospital, Kaohsiung 82445, Taiwan; 8 Division of Urology, Department of Surgery, E-Da Hospital, Kaohsiung 82445, Taiwan; 9 Department of Parasitology, College of Medicine, Kaohsiung Medical University, Kaohsiung 80708, Taiwan; rjlin@kmu.edu.tw; 10 Graduate Institute of Medicine, College of Medicine, Kaohsiung Medical University, Kaohsiung 80708, Taiwan; yichen83@kmu.edu.tw (Y.-C.L.); chaoyuah@kmu.edu.tw (C.-Y.C.); 11 Department of Urology, Kaohsiung Municipal Ta-Tung Hospital, Kaohsiung 80661, Taiwan; 12 Department of Anatomy, School of Medicine, College of Medicine, Kaohsiung Medical University, Kaohsiung 80708, Taiwan

## Error in Figures 6 and 8

In the original publication [1], there was a mistake in Figure 6C (Ketamine + Rapamycin group, immunofluorescence analysis of laminin) and Figure 8A (Western blot of Akt) as published. Image panels were mistakenly duplicated or misplaced during figure preparation. The corrected Figure 6C and Figure 8A appears below. The authors state that the scientific conclusions are unaffected. This correction was approved by the Academic Editor. The original publication has also been updated. 

## Figures and Tables

**Figure 6:**
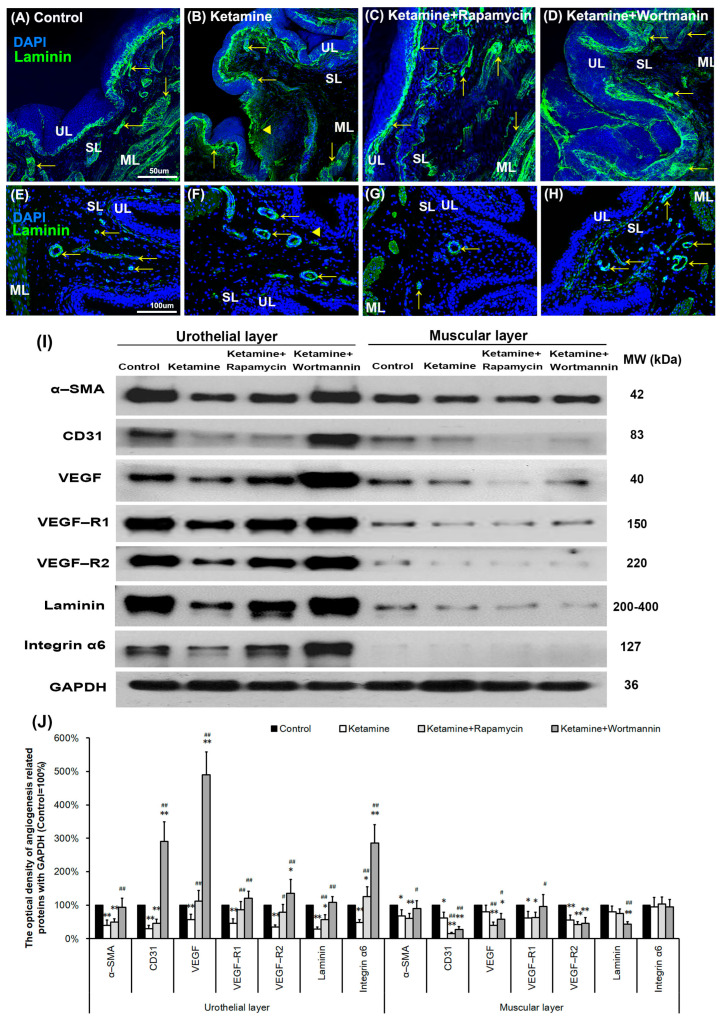
Autophagy altered bladder angiogenesis in the pathogenesis of KIC. (**A**–**H**) Immunofluorescence analysis of laminin, and α-SMA (**E**–**H**) (angiogenesis marker, arrows) expression after treatment with saline (**A**,**E**), ketamine (**B,F**), ketamine+rapamycin (**C**,**G**), and ketamine+wortmannin (**D**,**H**) in urothelial layer (UL), suburothelial layer (SL) and muscular layer (ML) of bladder tissue. In the ketamine+rapamycin group, bladder angiogenesis was mainly expressed in the marginal zone of lamina propria (suburothelial layer) near the urothelial basement membrane. The ketamine+wortmannin group significantly intensified the expression levels in the urothelial layer as compared to the other groups. The nuclei were counterstained by DAPI (blue). Yellow arrowheads indicate the denuded urothelial mucosa. Scale bar = 50 μm (Magnification ×200) and 100 μm (Magnification ×400). (**I**) Western blots of angiogenesis markers, including α-SMA, CD31 (endothelial marker), VEGF, VEGF-R1, VEGF-R2 (VEGF receptor), laminin, and integrin-α6 (laminin receptor), were quantified against glyceraldehyde-3-phosphate dehydrogenase (GAPDH). (**J**) Results were normalized to the control group (equal to 100%). α-SMA, alpha-smooth muscle actin; CD31, cluster of differentiation 31; VEGF, vascular endothelial growth factor. Data are expressed as means ± SD for n = 6, * *p* < 0.05, ** *p* < 0.01 versus the control group; ^#^ *p* < 0.05, ^##^ *p* < 0.01 versus the ketamine group.

**Figure 8:**
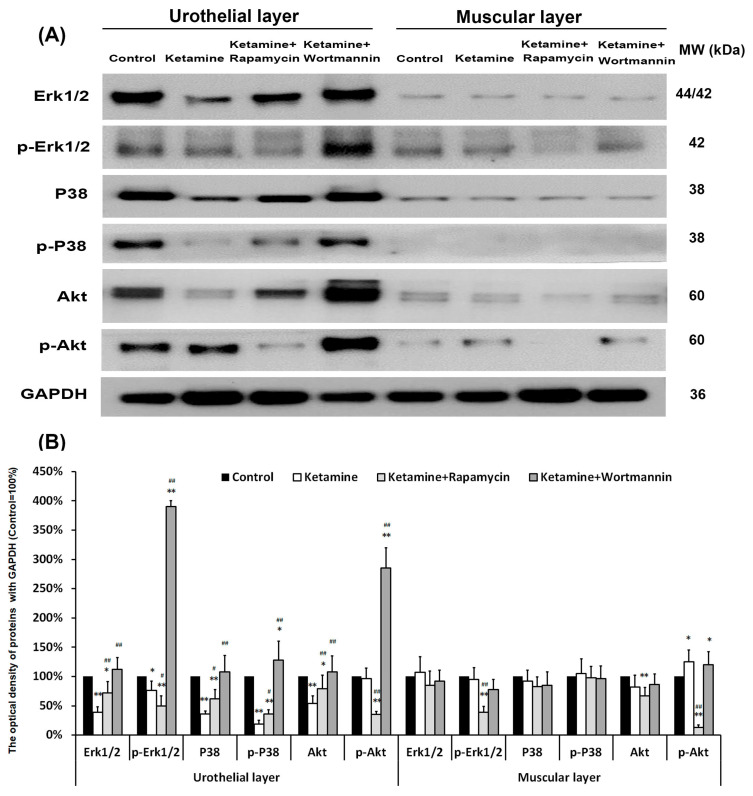
Western blots of signaling pathway kinase, including Erk1/2, p-Erk1/2, P38, p-P38, Akt, and p-Akt, were quantified against GAPDH (**A**). (**B**) Results were normalized to the control group (equal to 100%). The data revealed that treatment with ketamine significantly increased the Akt phosphorylation, but reduced the phosphorylation of Erk1/2 and p38 as compared to the control group. However, treatment with rapamycin increased the p38 phosphorylation, but suppressed the expression of p-Erk1/2 and p-Akt. Data were expressed as means ± SD for n = 6, * *p* < 0.05, ** *p* < 0.01 versus the control group; ^#^ *p* < 0.05, ^##^ *p* < 0.01 versus the ketamine group.
