# Supplementary material for: Autophagy Alters Bladder Angiogenesis and Improves Bladder Hyperactivity in the Pathogenesis of Ketamine-Induced Cystitis in a Rat Model
Source: Biology (Basel). 2021 May 30;10(6):488. doi: 10.3390/biology10060488 (PMC8228861; doi:10.3390/biology10060488)

Western Blot

Urothelial layer (UL)

Muscular layer (ML)

Control (C)

Ketamine (K)

Ketamine+ Rapamycin (K+R)

Ketamine+ Wortmannin (K+W)

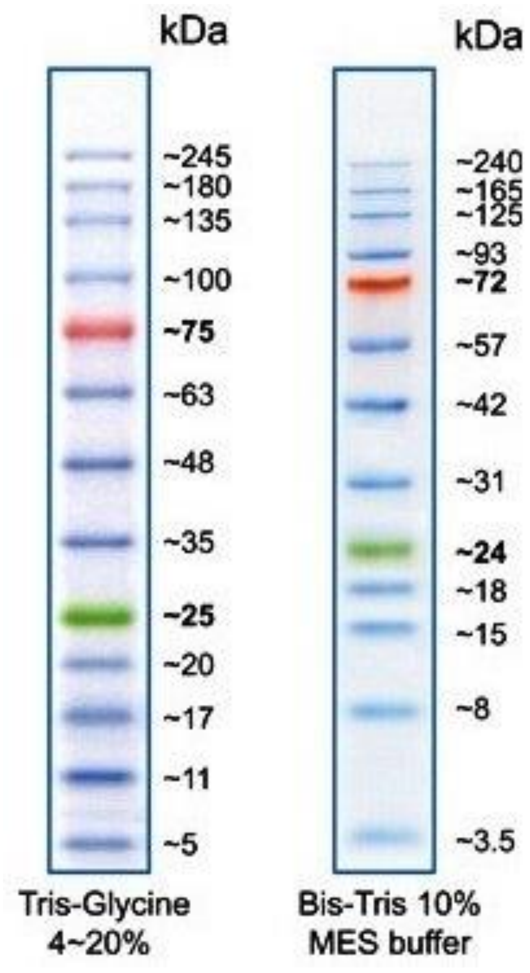

Western blot

Protein: mTOR

1^0 Ab: Cell Signaling, rabbit monoclonal IgG

Catalog number: no.2983

Molecular weight (kDa):289 kDa

Working concentration: 1:1000

Gel (%): 8%

Control (C) Ketamine (K) Ketamine+ Rapamycin (K+R)

Ketamine+ Wortmannin (K+W)

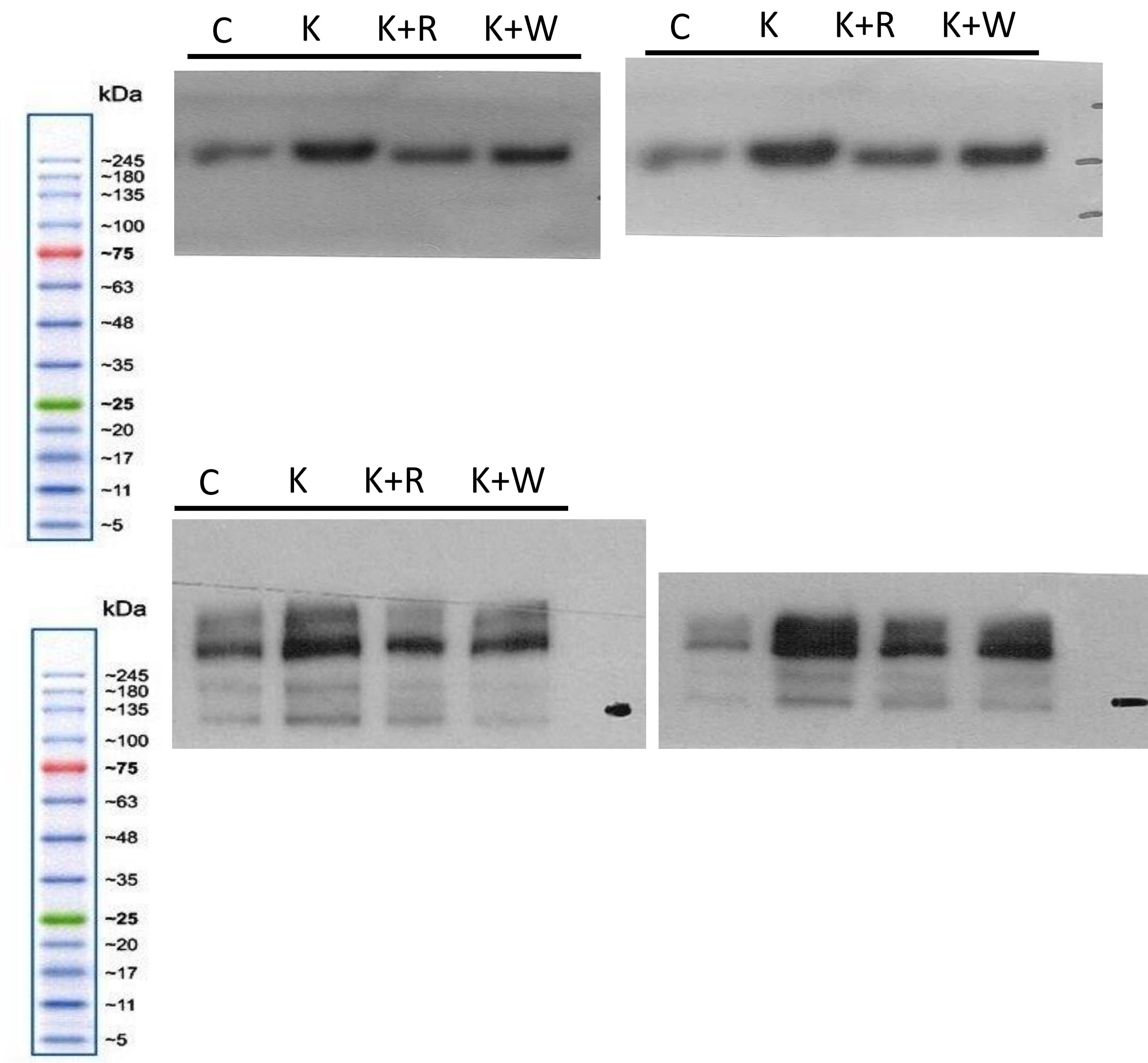

Western blot

Protein: p-mTOR

1^{0} Ab: Cell Signaling, rabbit monoclonal IgG

Catalog number: no.5536

Molecular weight (kDa):289 kDa

Working concentration: 1:1000

Gel (%): 8%

Control (C) Ketamine (K) Ketamine+ Rapamycin (K+R)

Ketamine+ Wortmannin (K+W)

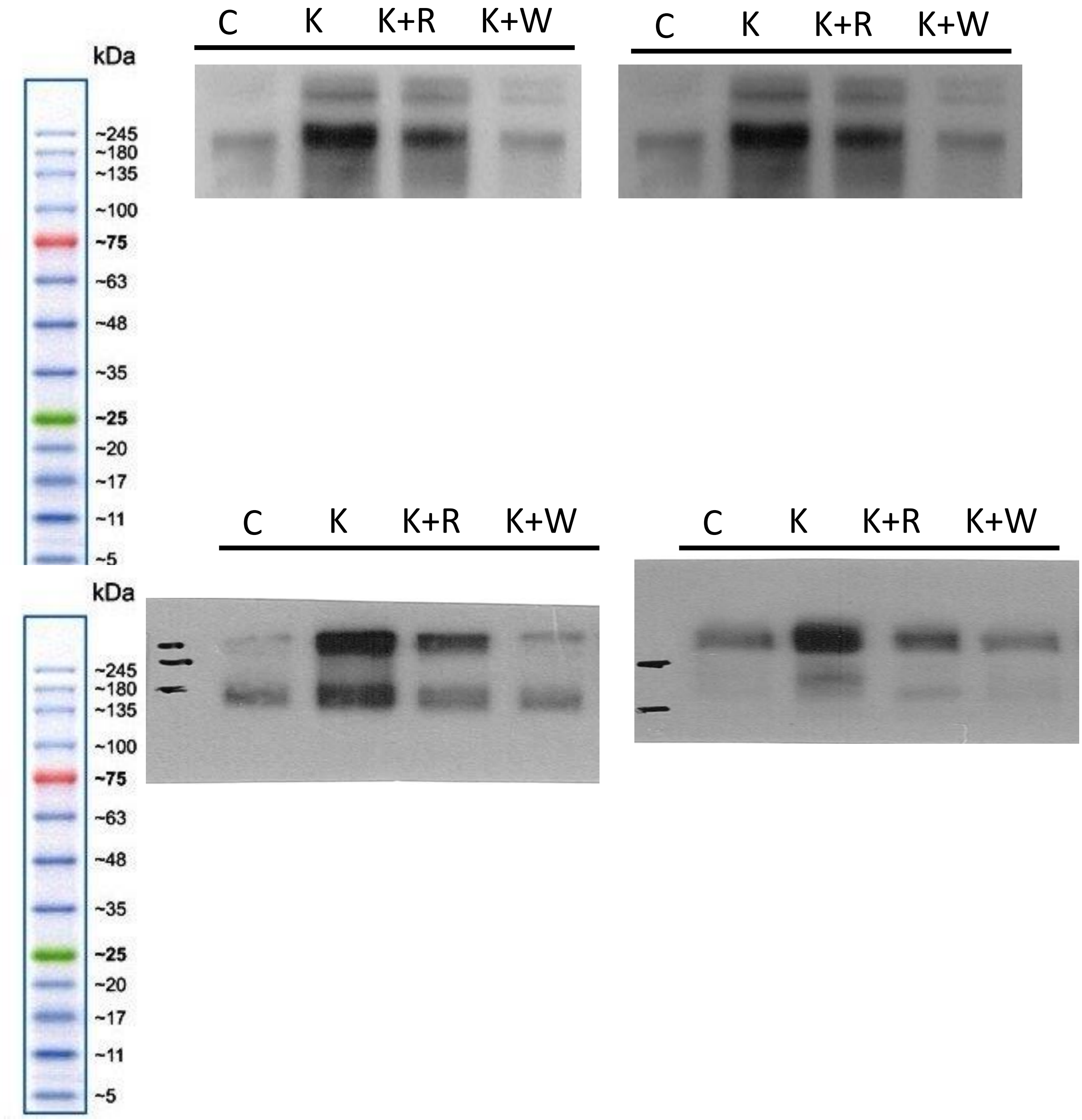

Western blot

Protein: Atg 12

1^0 Ab: Proteintech, rabbit polyclonal IgG

Catalog number: no.11264-1-AP   Molecular weight (kDa):48-55 kDa

Working concentration: 1:1000   Gel (%): 8%

Control (C)   Ketamine (K)   Ketamine+ Rapamycin (K+R)

Ketamine+ Wortmannin (K+W)

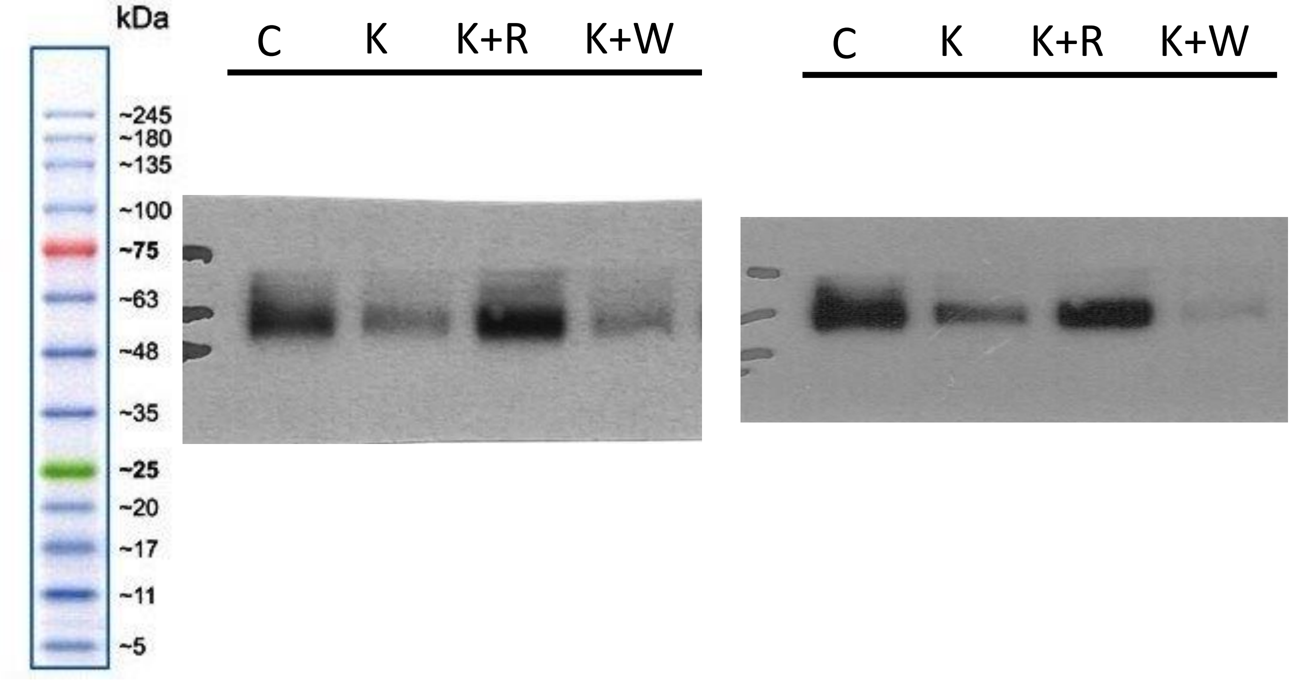

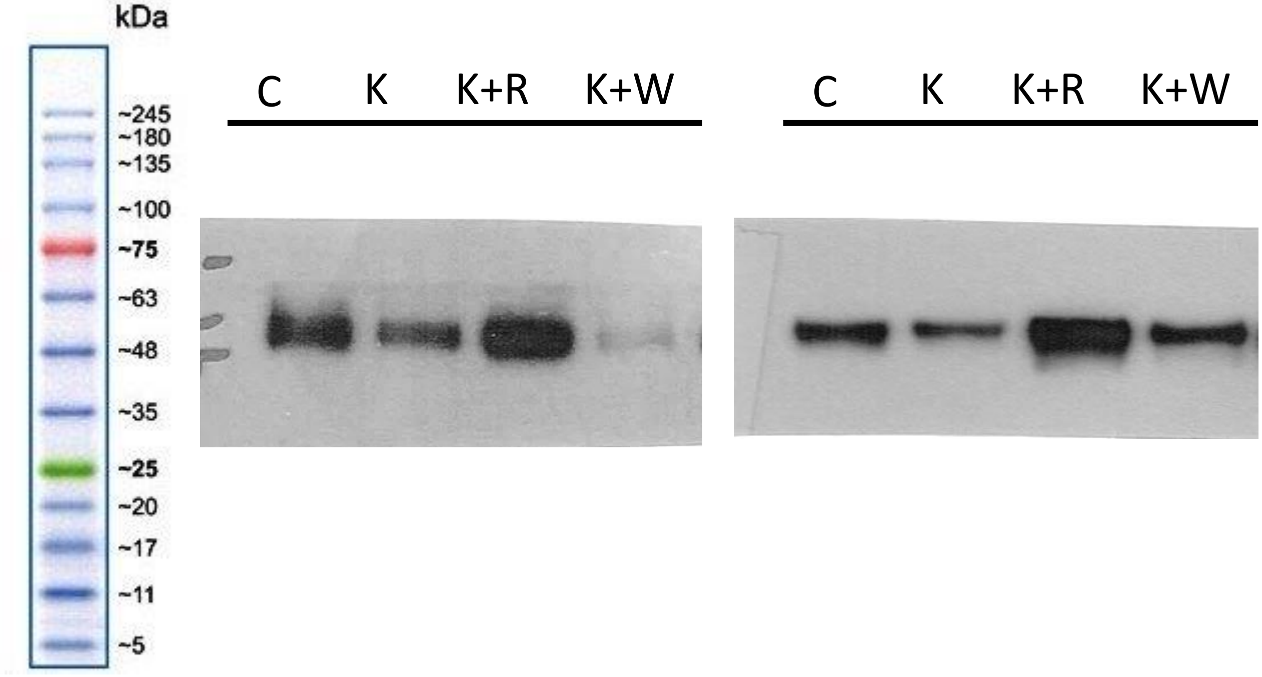

Western blot

Protein: Atg 7

1^0 Ab: Cell Signaling, rabbit monoclonal IgG

Catalog number: no.8558

Molecular weight (kDa):78 kDa

Working concentration: 1:1000

Gel (%): 8%

Control (C) Ketamine (K) Ketamine+ Rapamycin (K+R)

Ketamine+ Wortmannin (K+W)

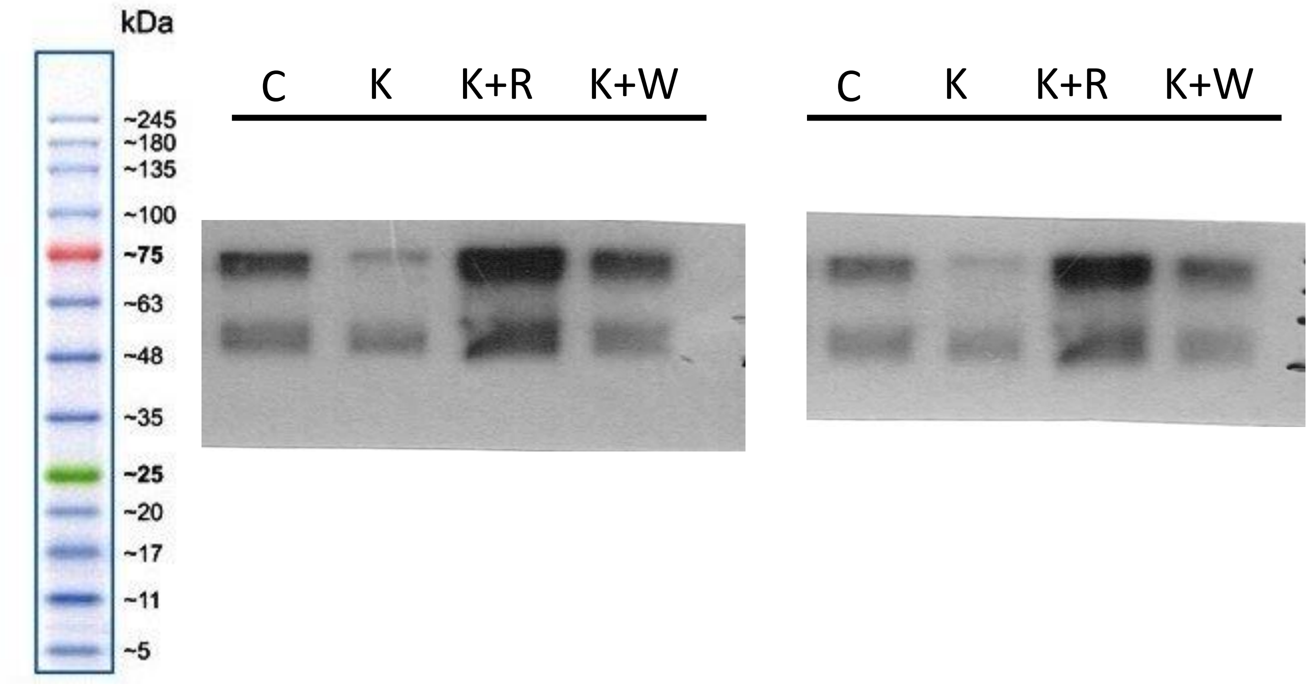

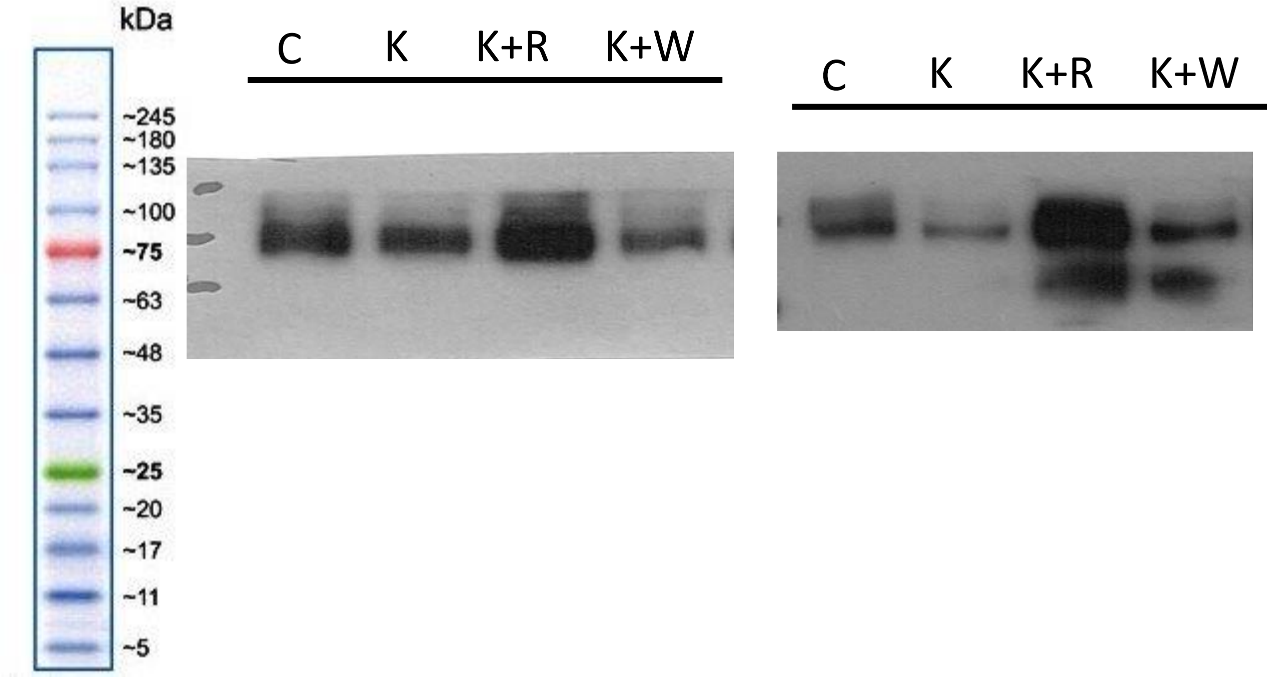

Western blot

Protein: Beclin 1 (Atg 6)

Catalog number: no.NBP1-76648

Working concentration: 1:1000

1^0 Ab: Novus, rabbit polyclonal IgG

Molecular weight (kDa):52-55 kDa

Gel (%): 8%

Control (C) Ketamine (K) Ketamine+ Rapamycin (K+R)

Ketamine+ Wortmannin (K+W)

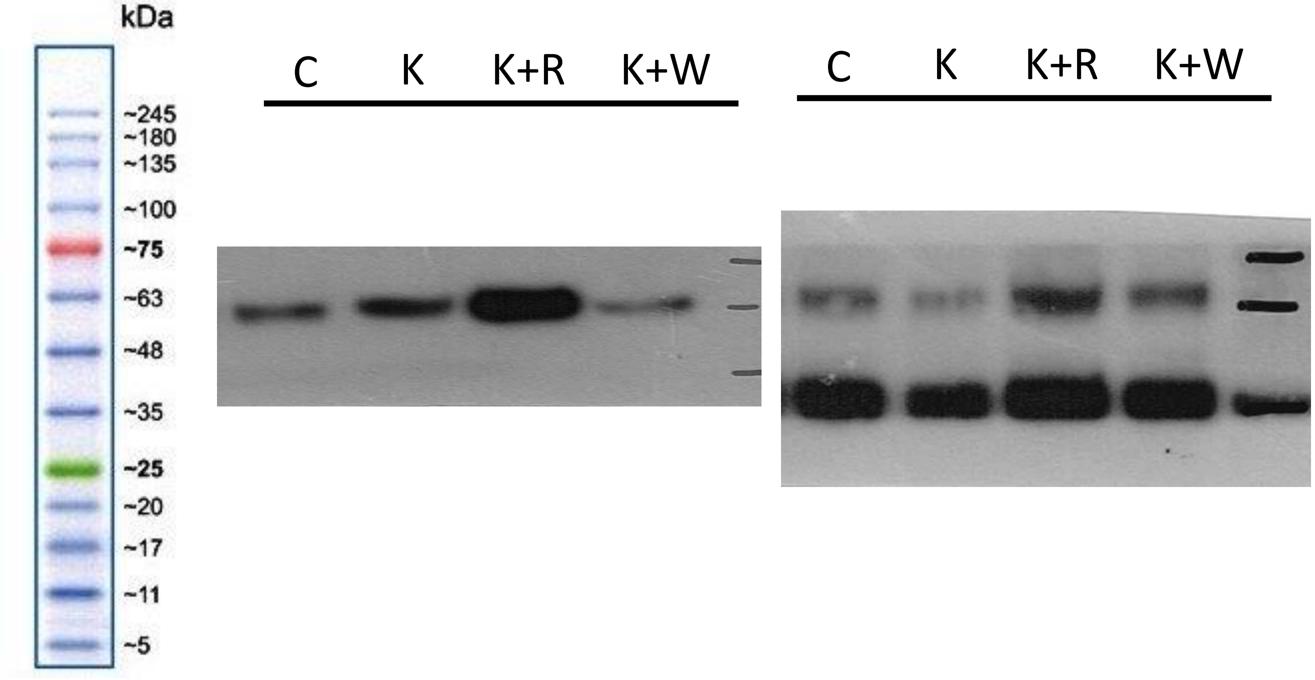

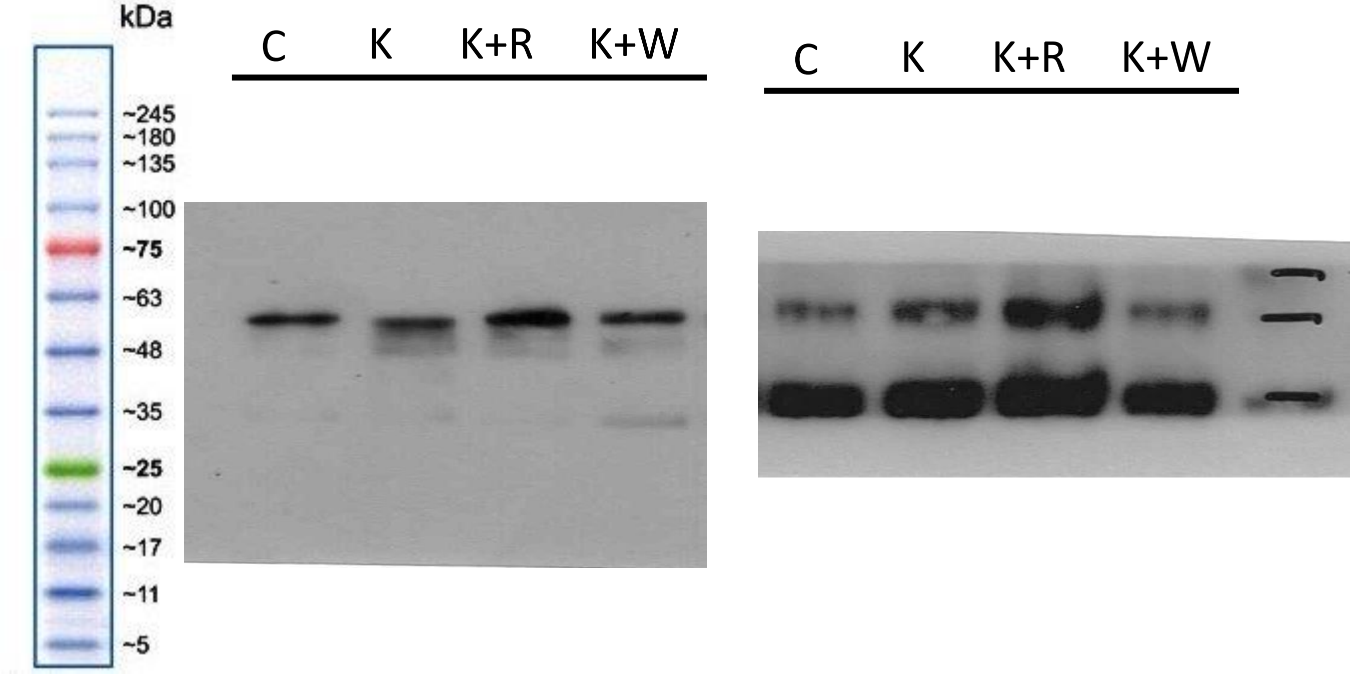

Western blot

Protein: LC3 (Atg 8) **1^0 Ab:** Cell Signaling, rabbit monoclonal IgG

Catalog number: no.4599 Molecular weight (kDa):14, 16 kDa

Working concentration: 1:1000 Gel (%): 8%

Control (C) Ketamine (K) Ketamine+ Rapamycin (K+R)

Ketamine+ Wortmannin (K+W)

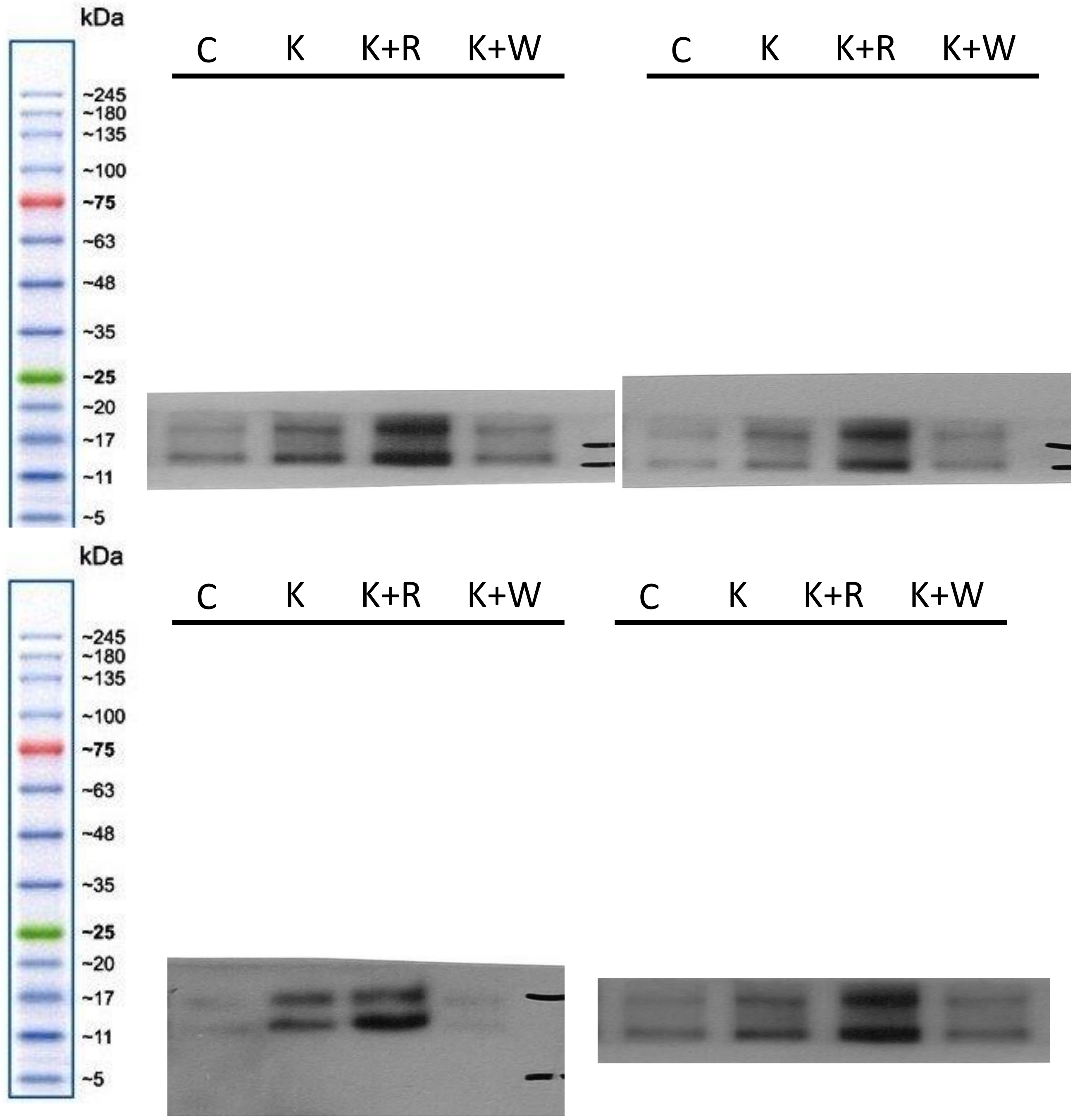

Western blot

Protein: VPS 34

Catalog number: no. 12452-1-AP

Working concentration: 1:1000

1^0 Ab: Cell Signaling, rabbit polyclonal IgG

Molecular weight (kDa):100 kDa

Gel (%): 8%

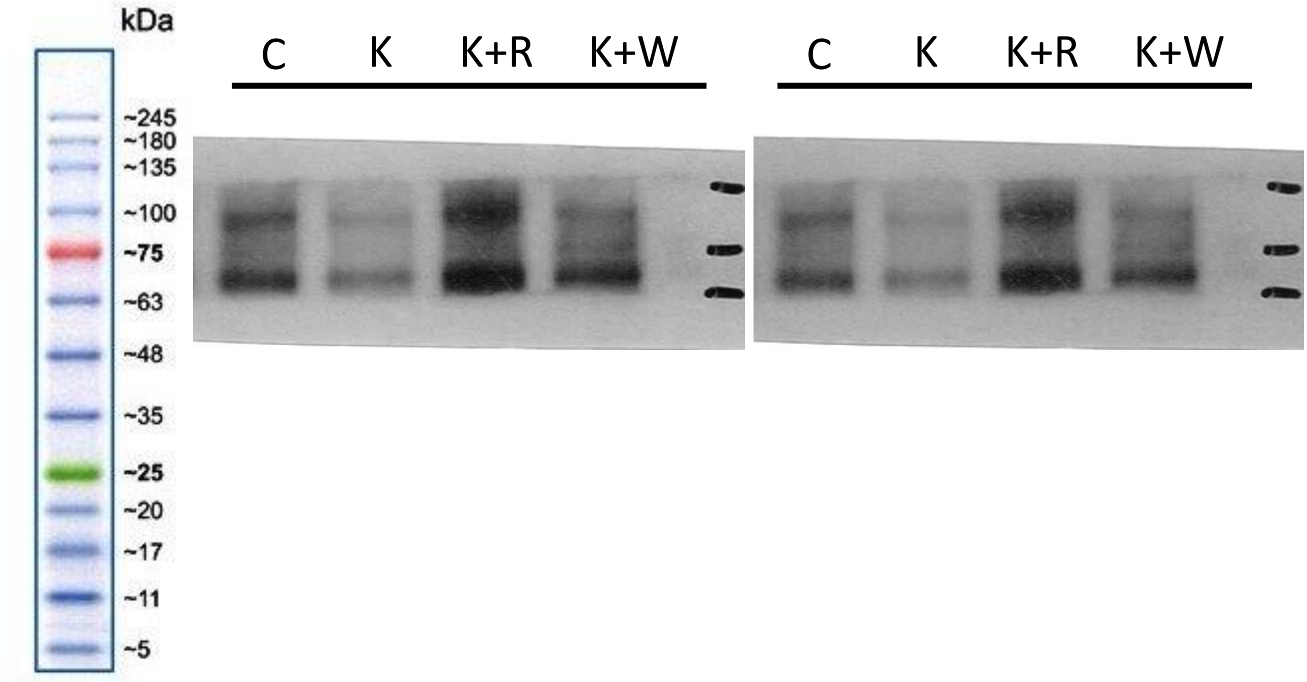

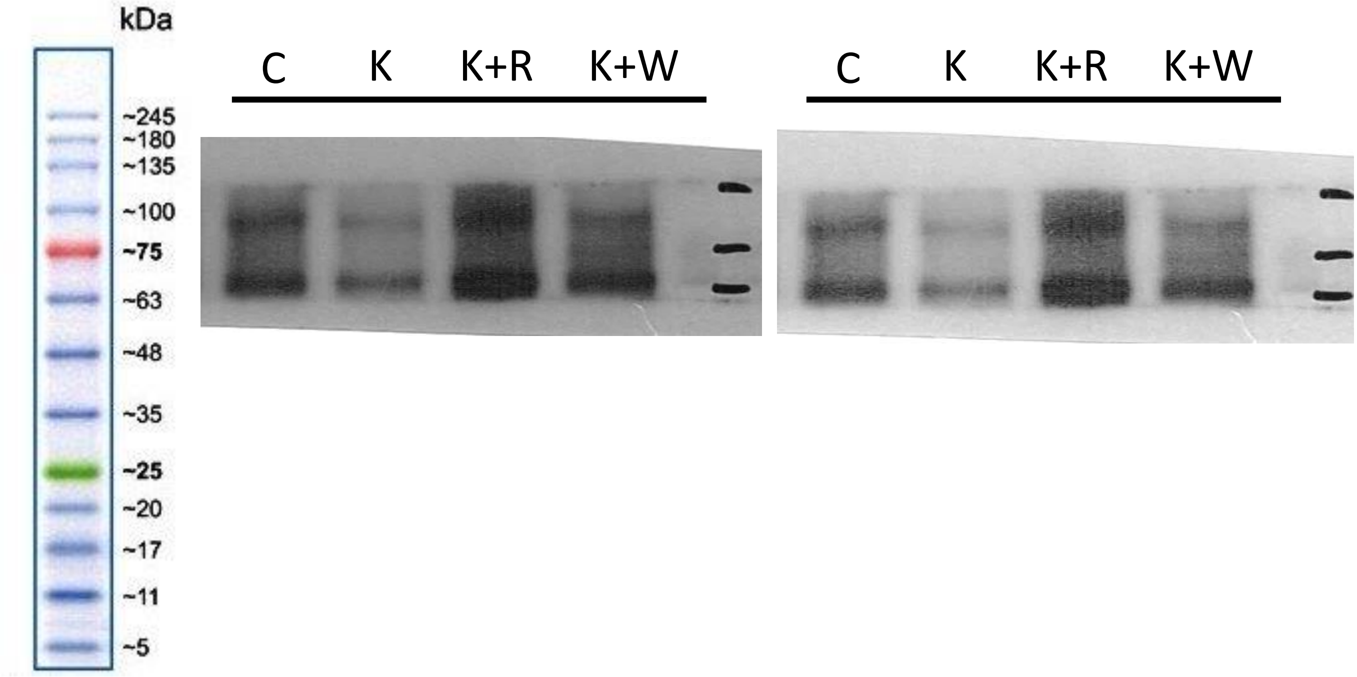

Western blot

Protein: ß-Actin

1^0 Ab: Cell Signaling, rabbit monoclonal IgG

Catalog number: no.4970S

Molecular weight (kDa):43 kDa

Working concentration: 1:5000

Gel (%): 8%

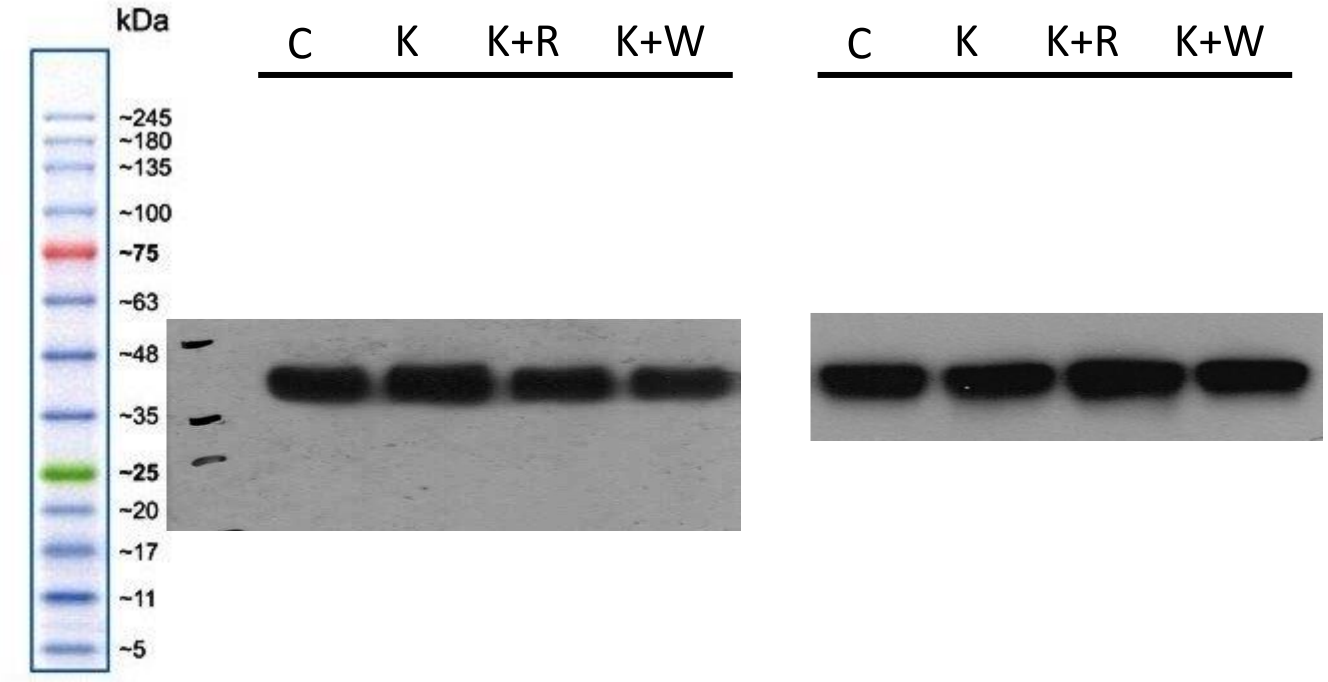

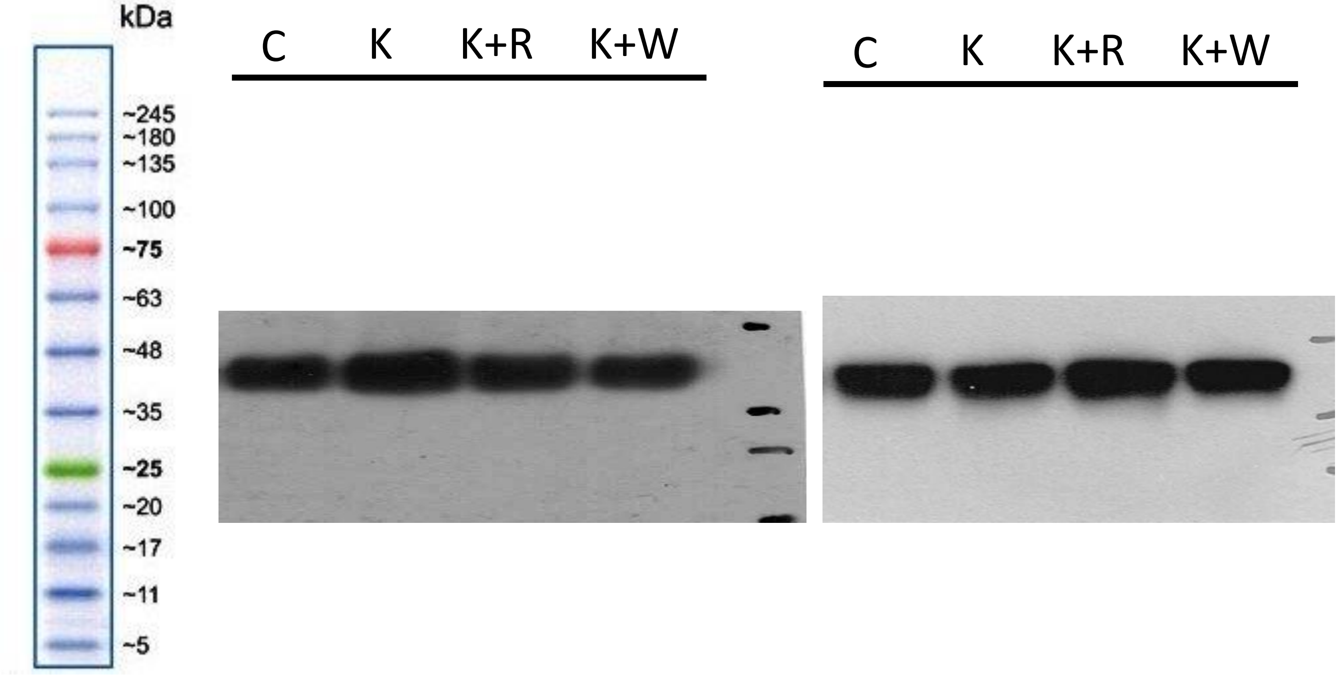

Western blot

Protein: ß-Actin

1^0 Ab: Cell Signaling, rabbit monoclonal IgG

Catalog number: no.4970S

Molecular weight (kDa):43 kDa

Working concentration: 1:5000

Gel (%): 8%

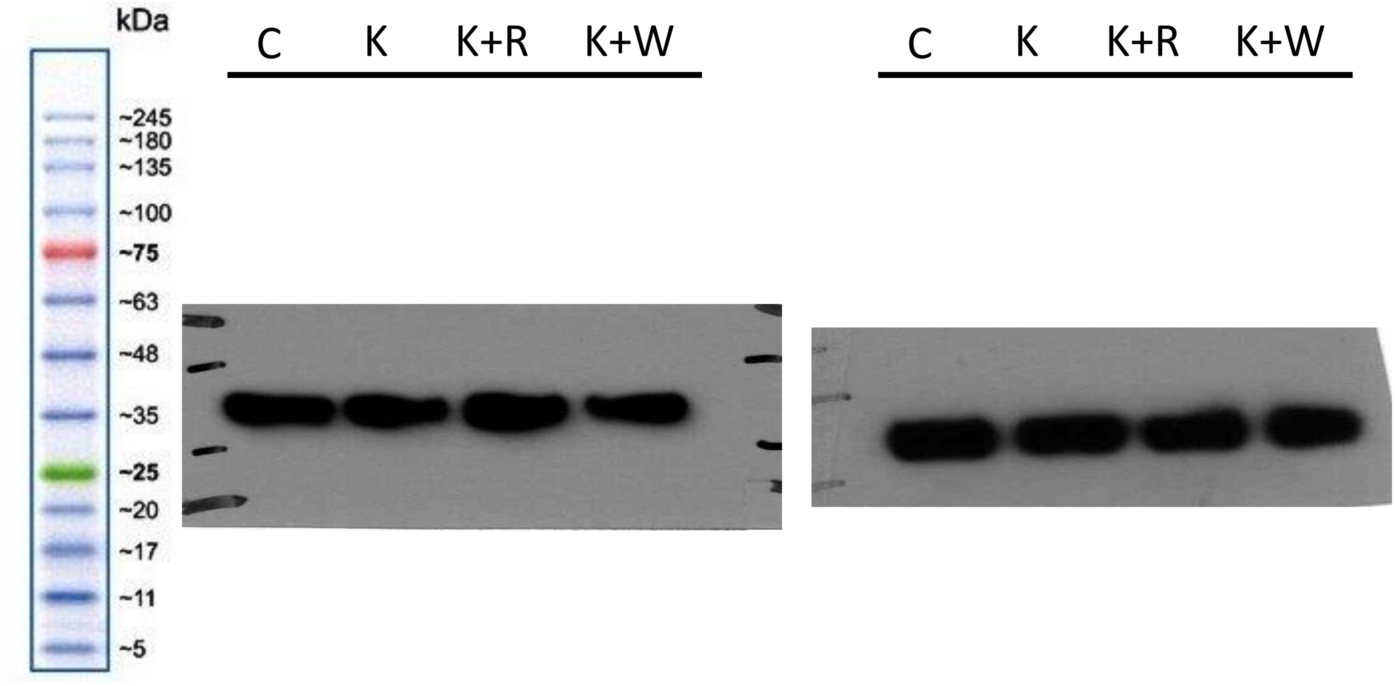

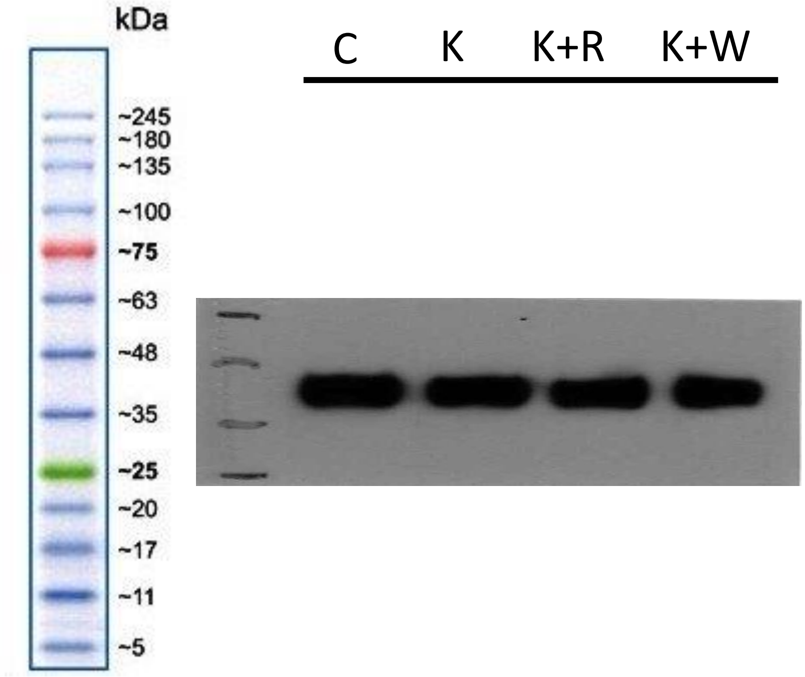

Western blot

Protein: α-SMA

1^{0} Ab: Abcam, rabbit monoclonal IgG

Catalog number: no.ab5694

Molecular weight (kDa):40 kDa

Working concentration: 1:5000

Gel (%): 8%

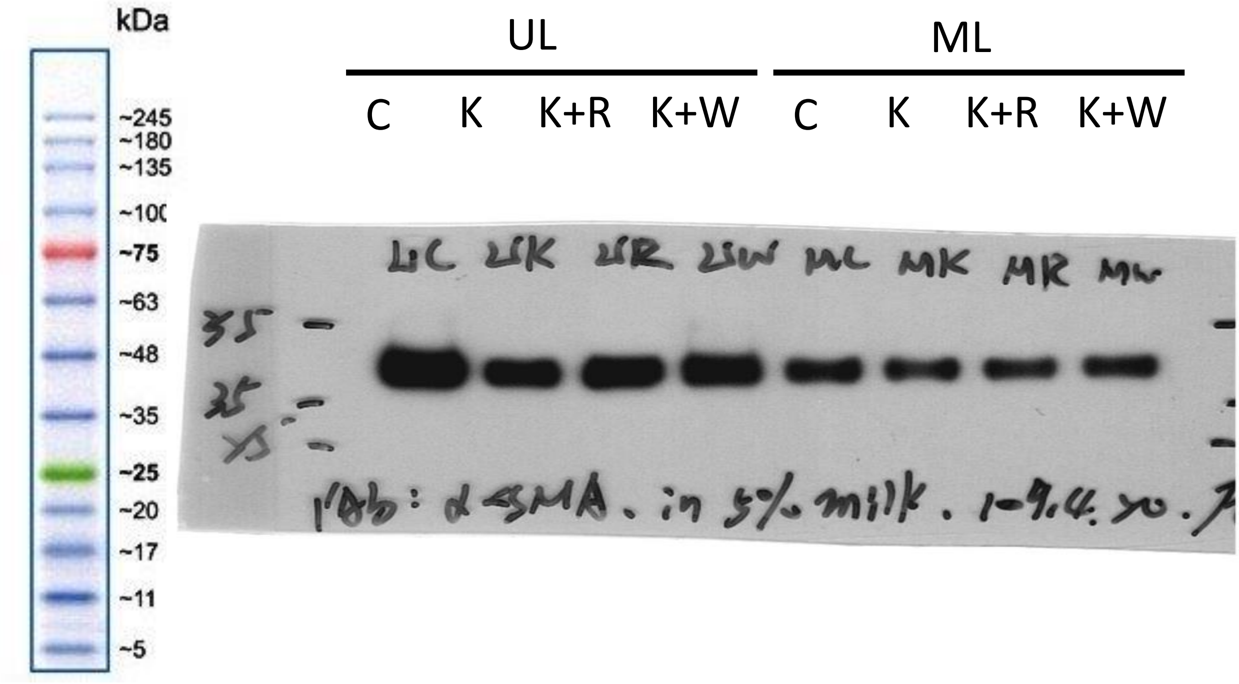

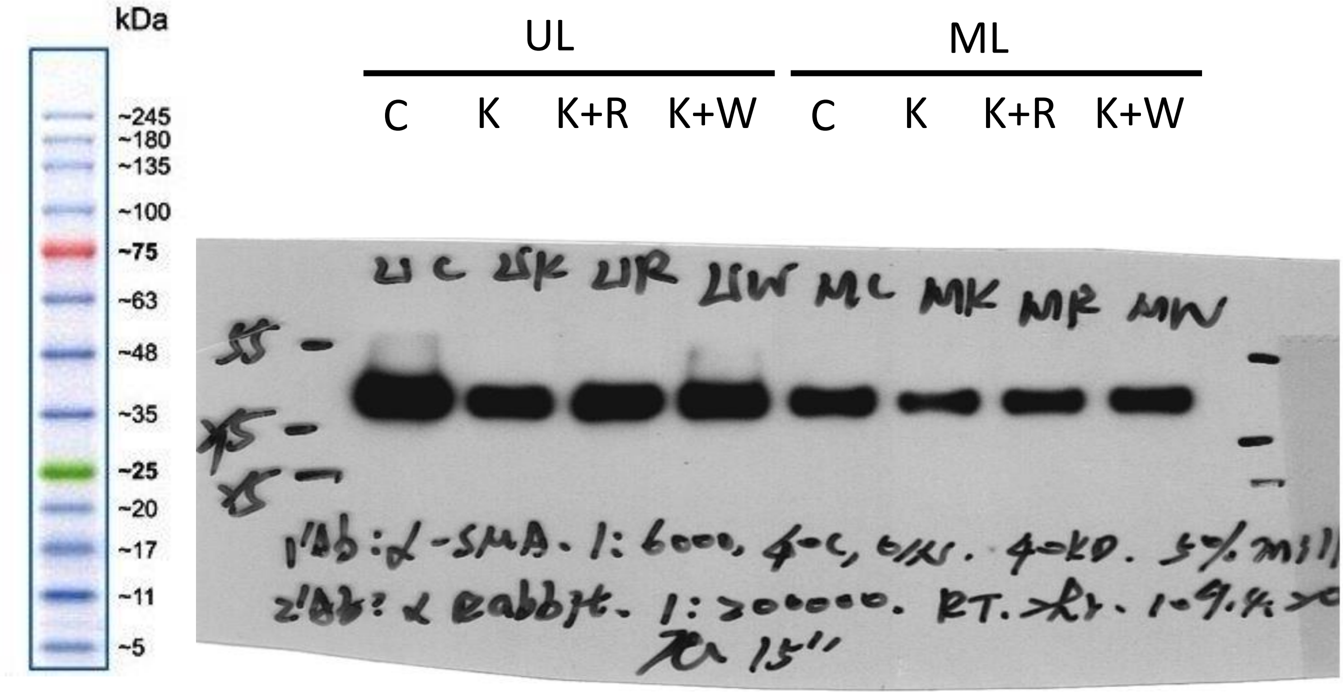

Western blot

Protein: α-SMA

1⁰ Ab: Abcam, rabbit monoclonal IgG

Catalog number: no.ab5694

Molecular weight (kDa):40 kDa

Working concentration: 1:5000

Gel (%): 8%

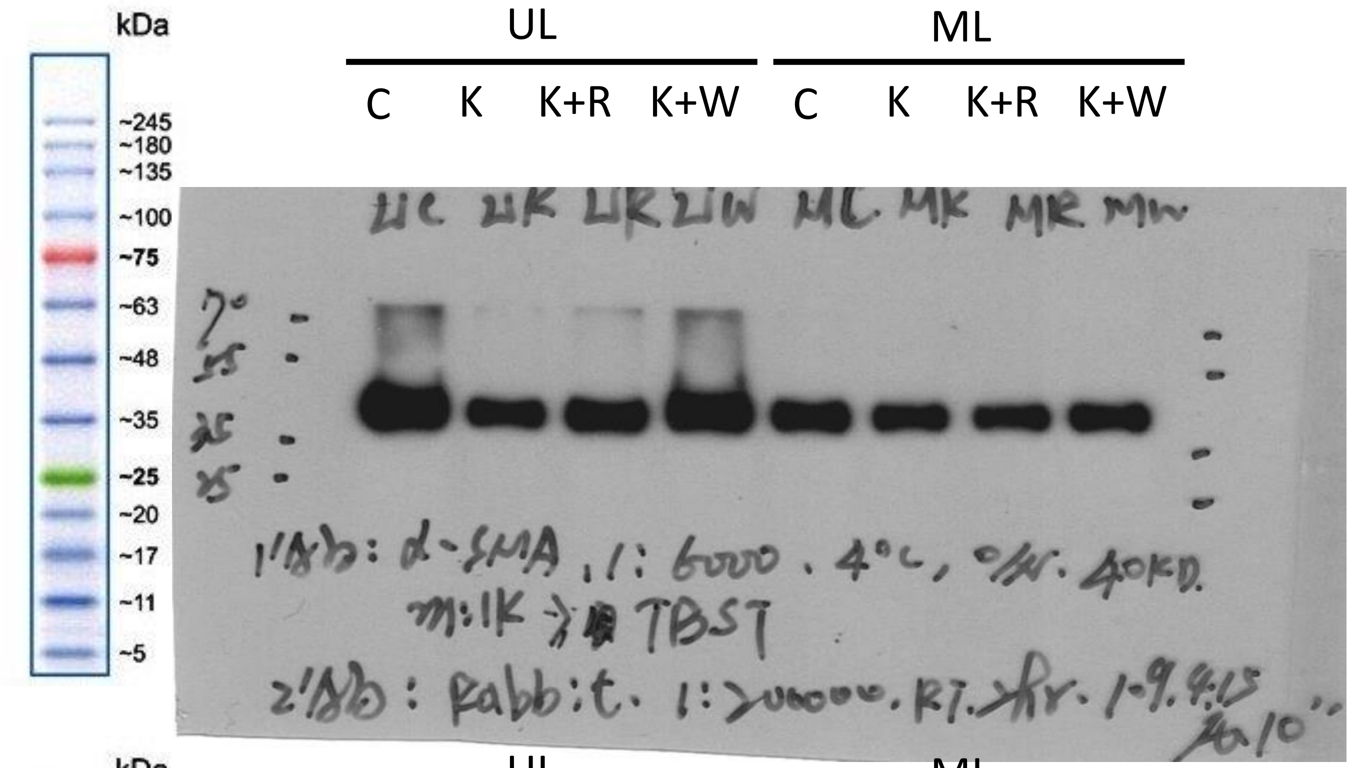

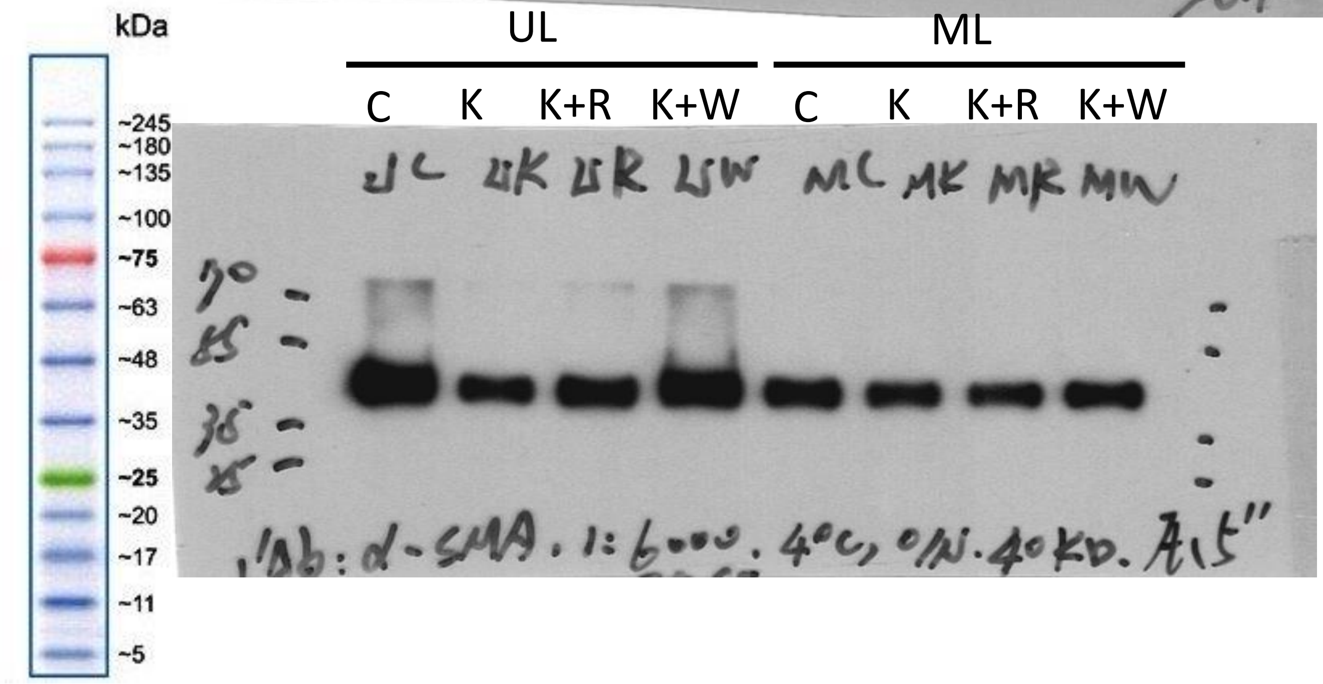

Western blot

Protein: α-SMA

1^0 Ab: Abcam, rabbit monoclonal IgG

Catalog number: no.ab5694

Molecular weight (kDa):40 kDa

Working concentration: 1:5000

Gel (%): 8%

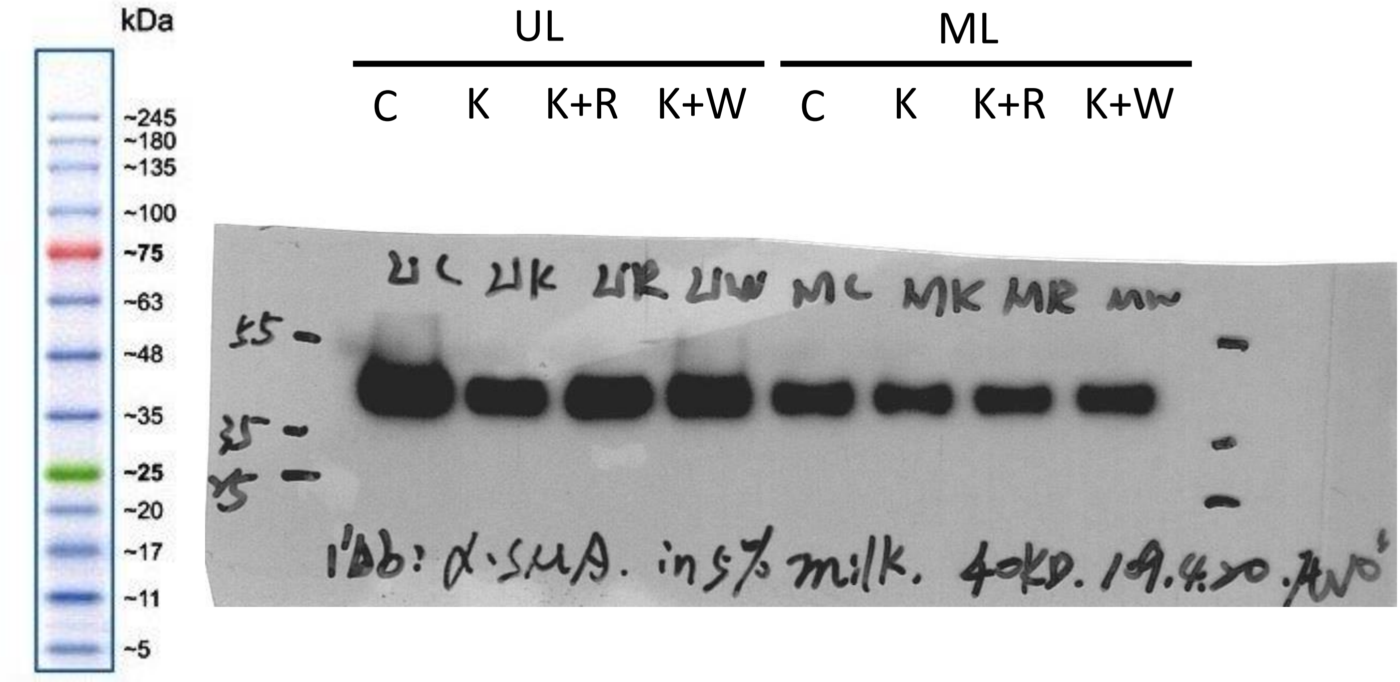

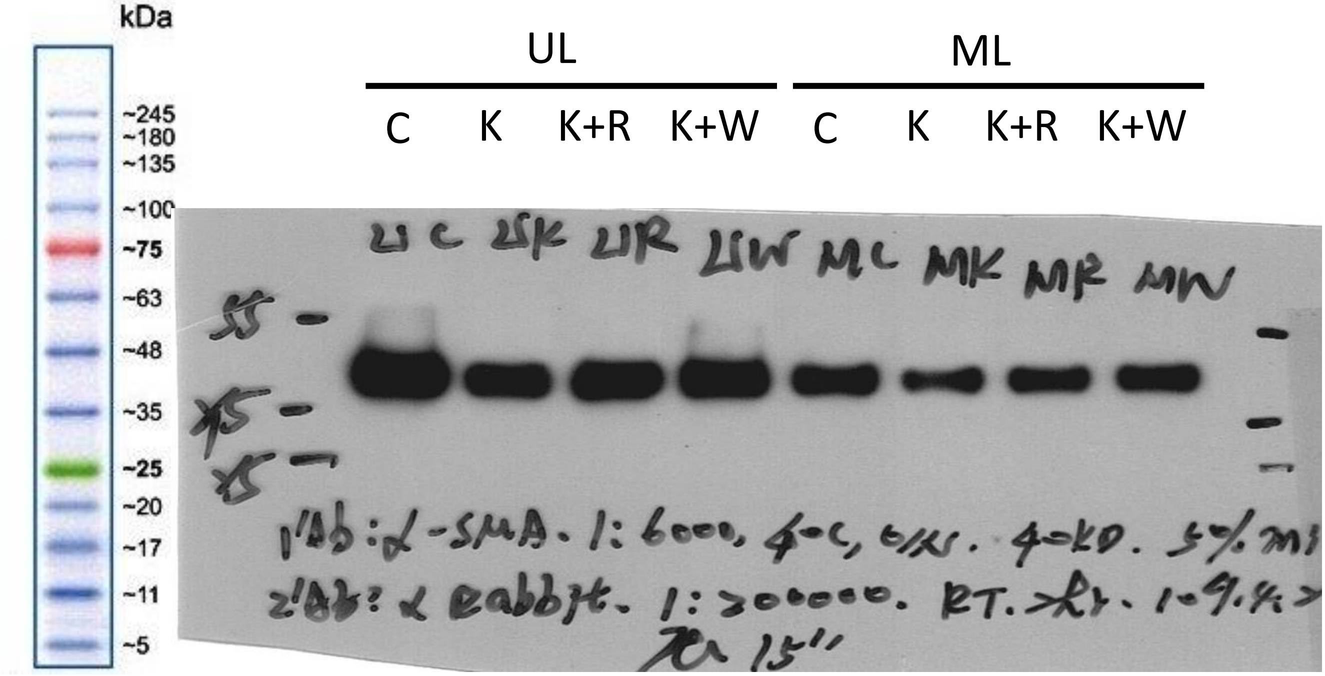

Western blot

Protein: α-SMA

1^0 Ab: Abcam, rabbit monoclonal IgG

Catalog number: no.ab5694

Molecular weight (kDa):40 kDa

Working concentration: 1:5000

Gel (%): 8%

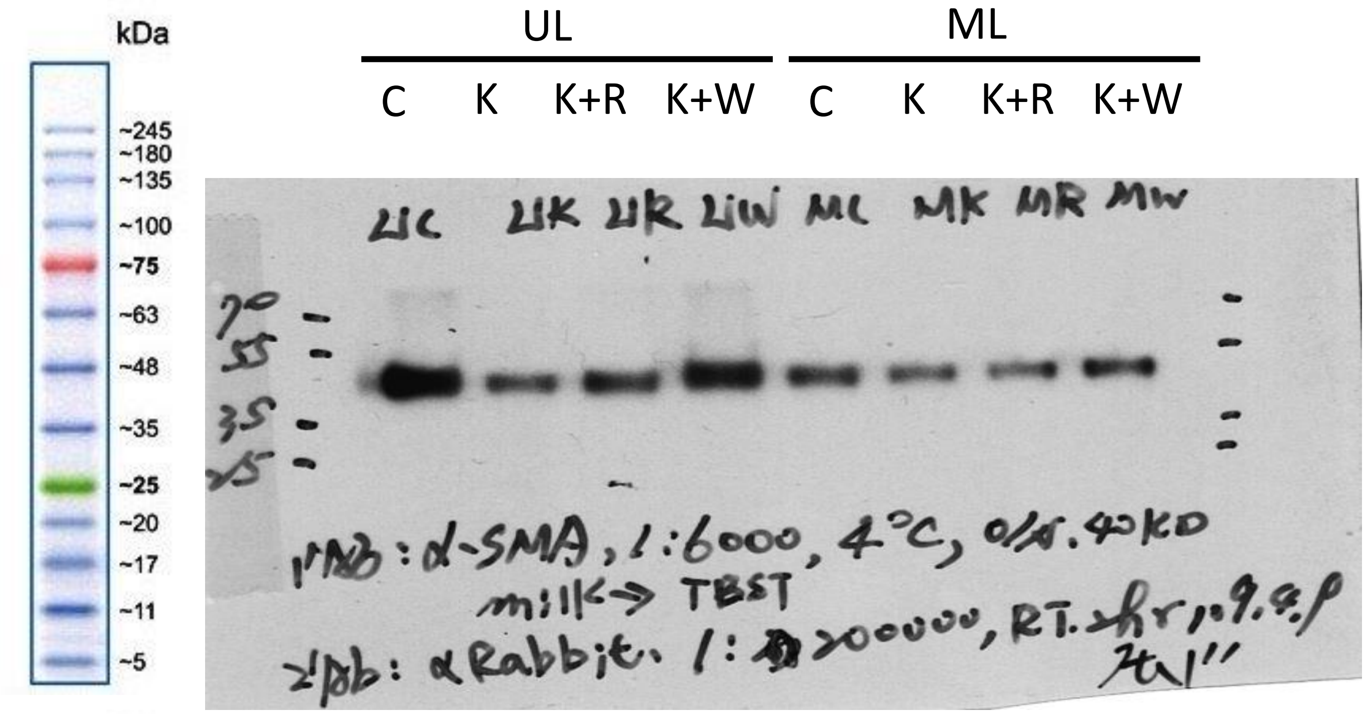

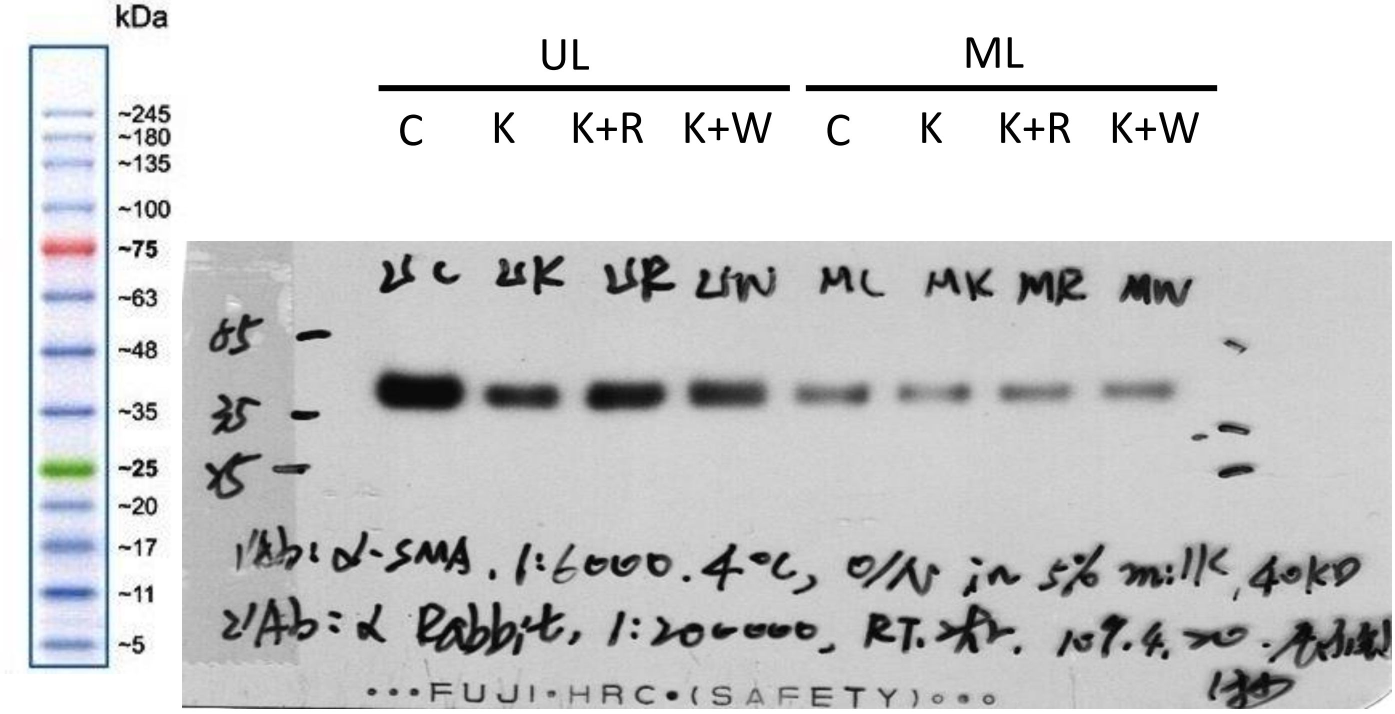

Western blot

Protein: CD31

Catalog number: no.9498

Working concentration: 1:3000

1^0 Ab: Abcam, mouse monoclonal IgG

Molecular weight (kDa):83 kDa

Gel (%): 8%

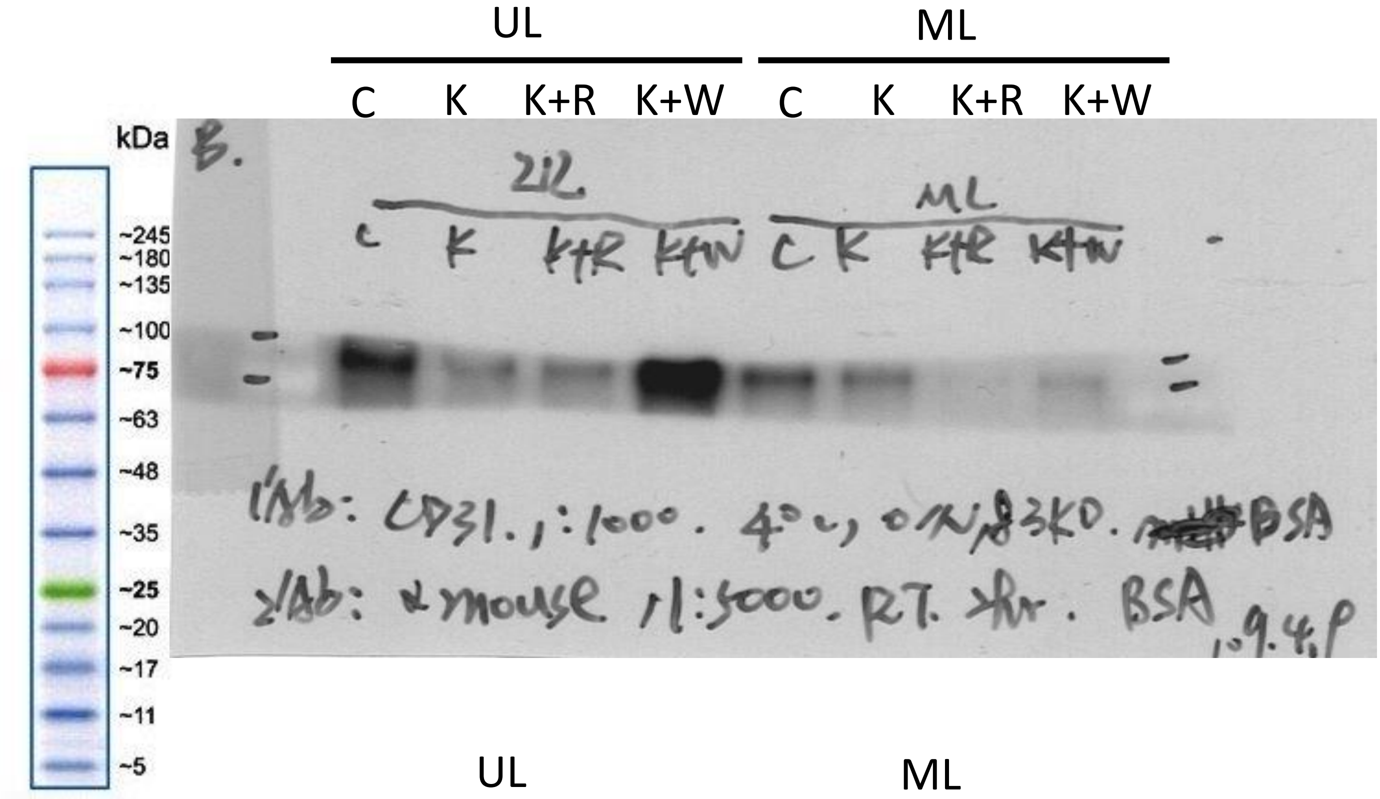

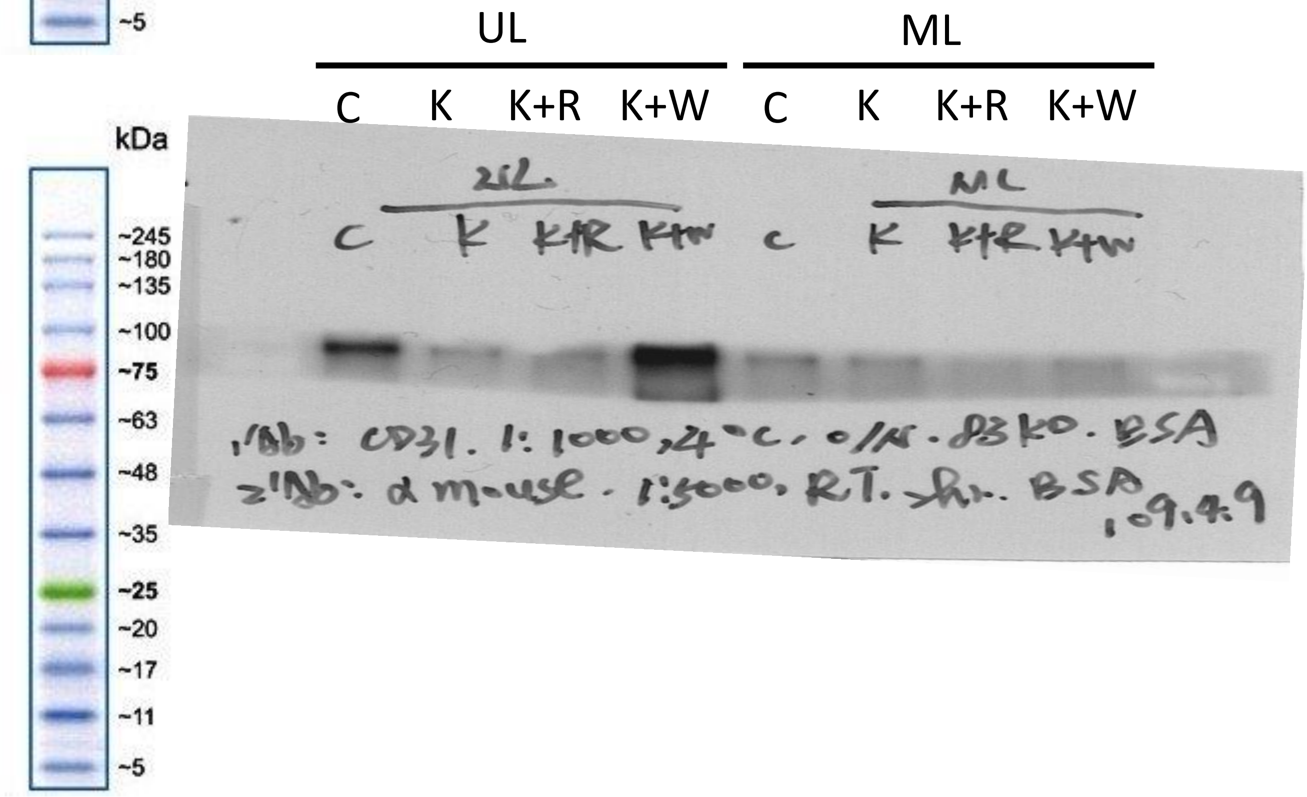

Western blot

Protein: CD31

1^0 Ab: Abcam, mouse monoclonal IgG

Catalog number: no.9498

Molecular weight (kDa):83 kDa

Working concentration: 1:3000

Gel (%): 8%

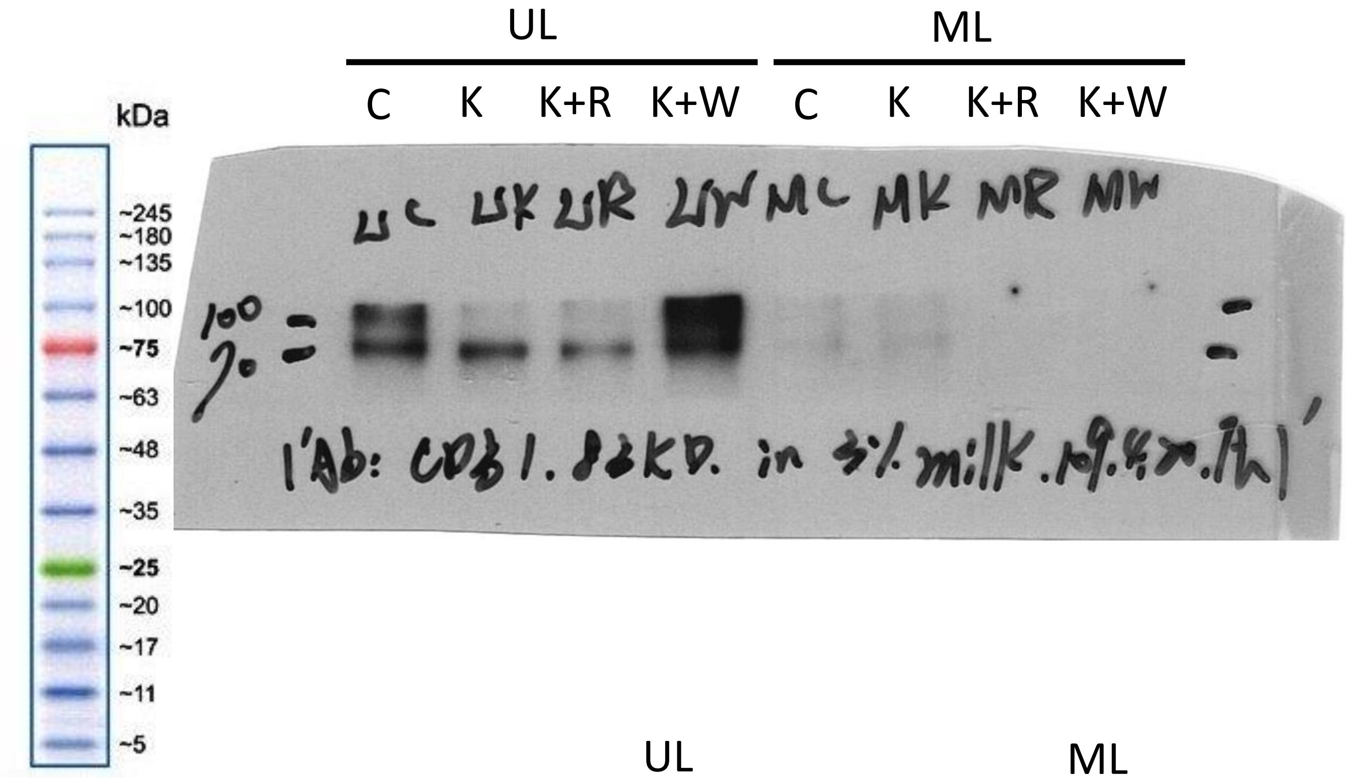

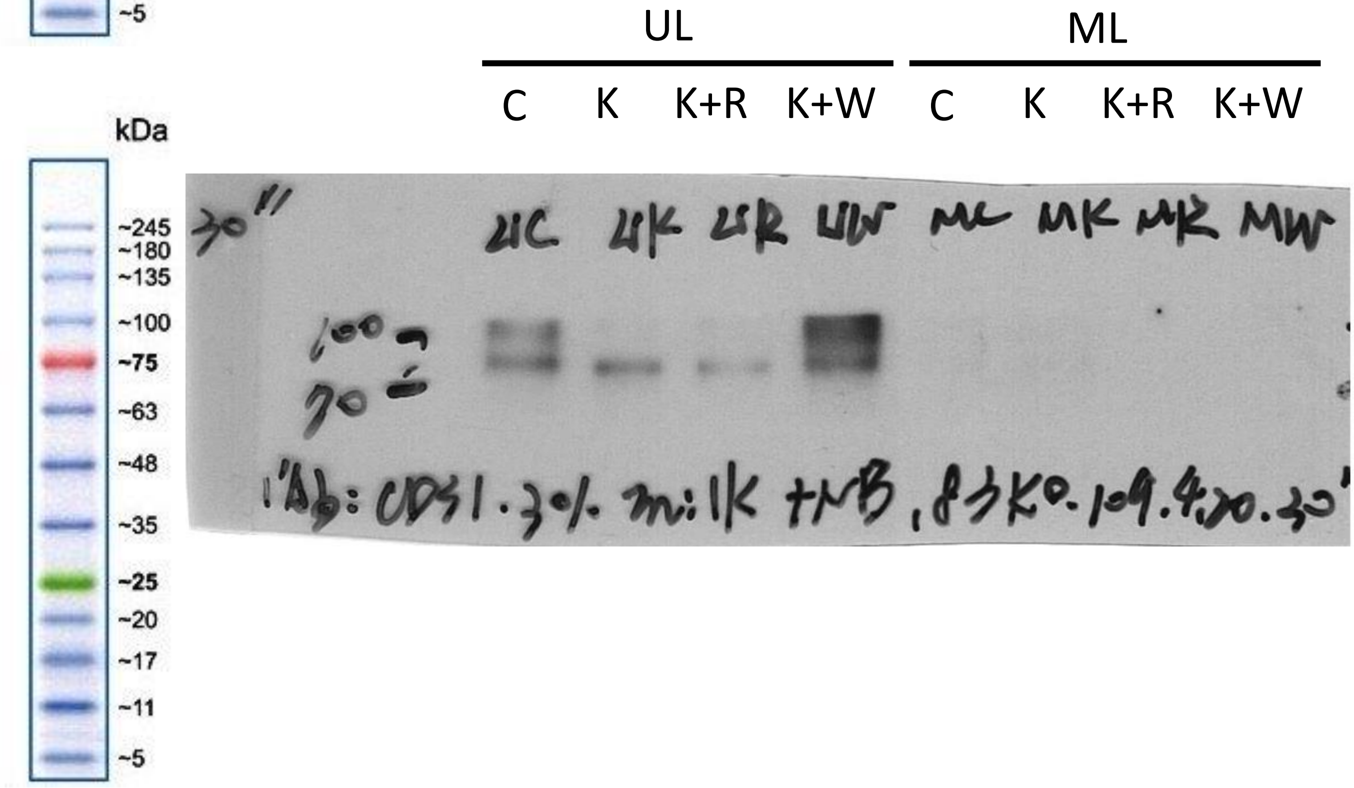

Western blot

Protein: VEGF

1^0 Ab: Millipore, mouse monoclonal IgG

Catalog number: no.05443

Molecular weight (kDa):40 kDa

Working concentration: 1:2000

Gel (%): 8%

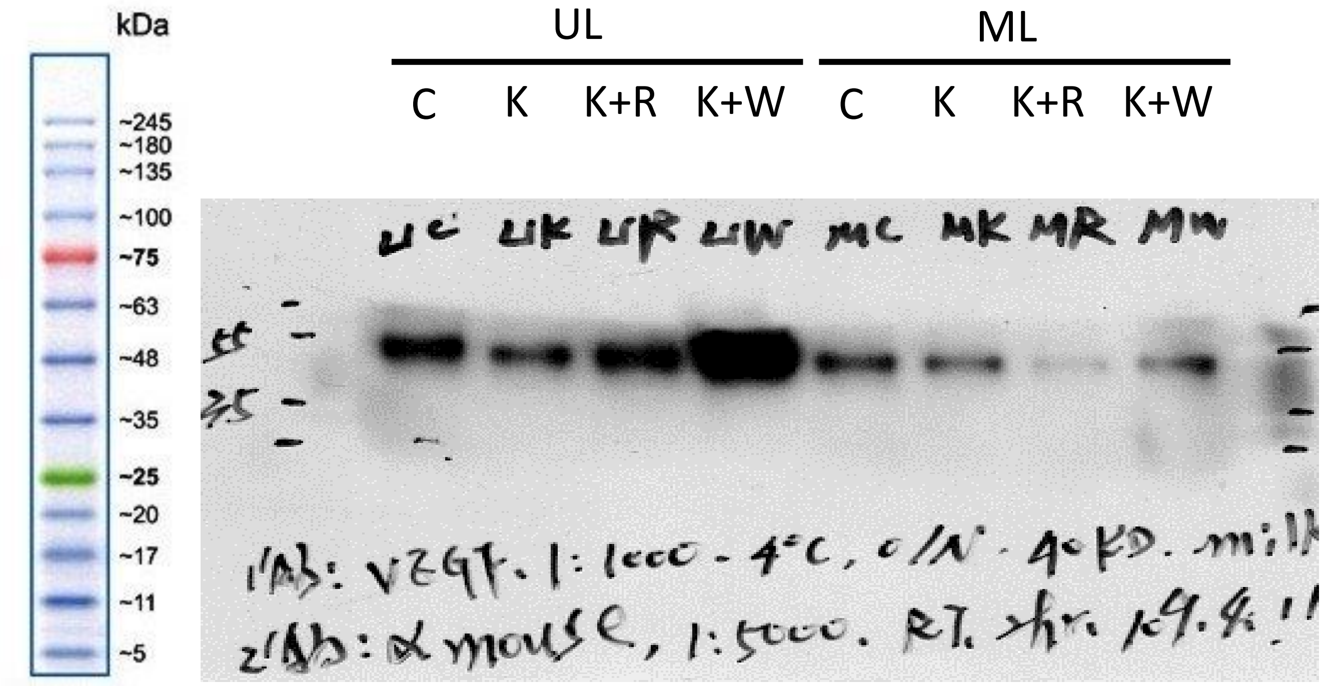

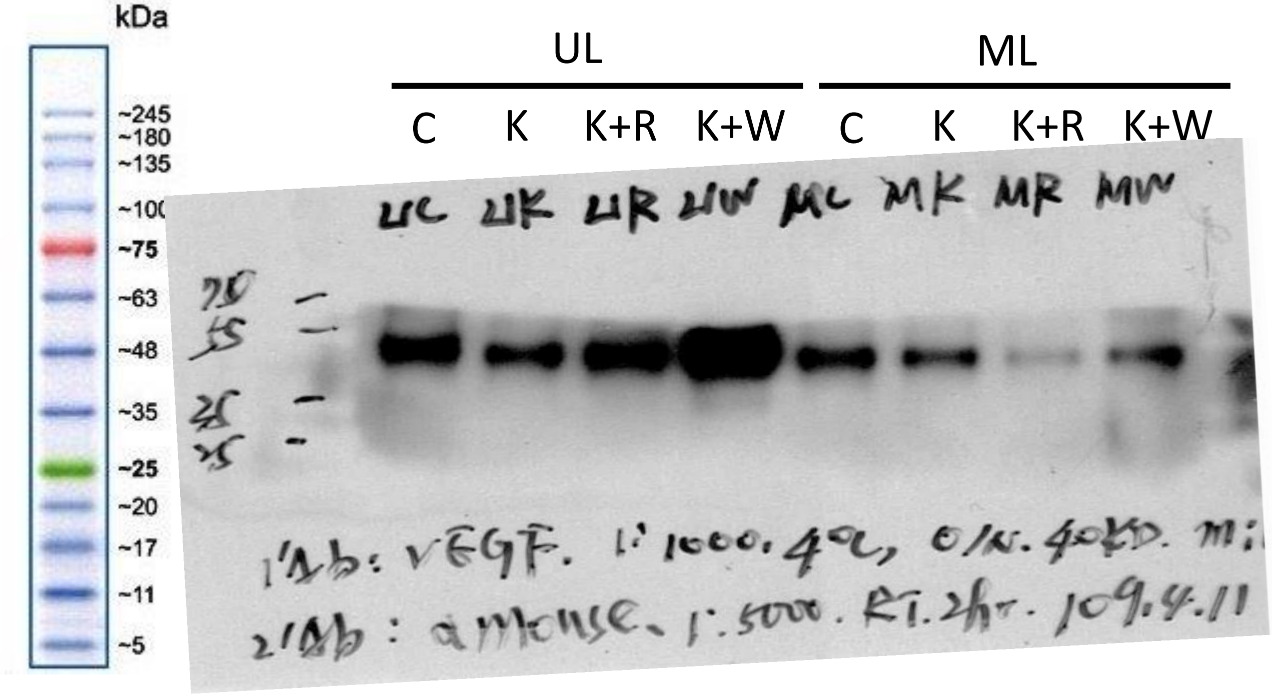

Western blot

Protein: VEGF

Catalog number: no.05443

Working concentration: 1:2000

1[0] Ab: Millipore, mouse monoclonal IgG

Molecular weight (kDa):40 kDa

Gel (%): 8%

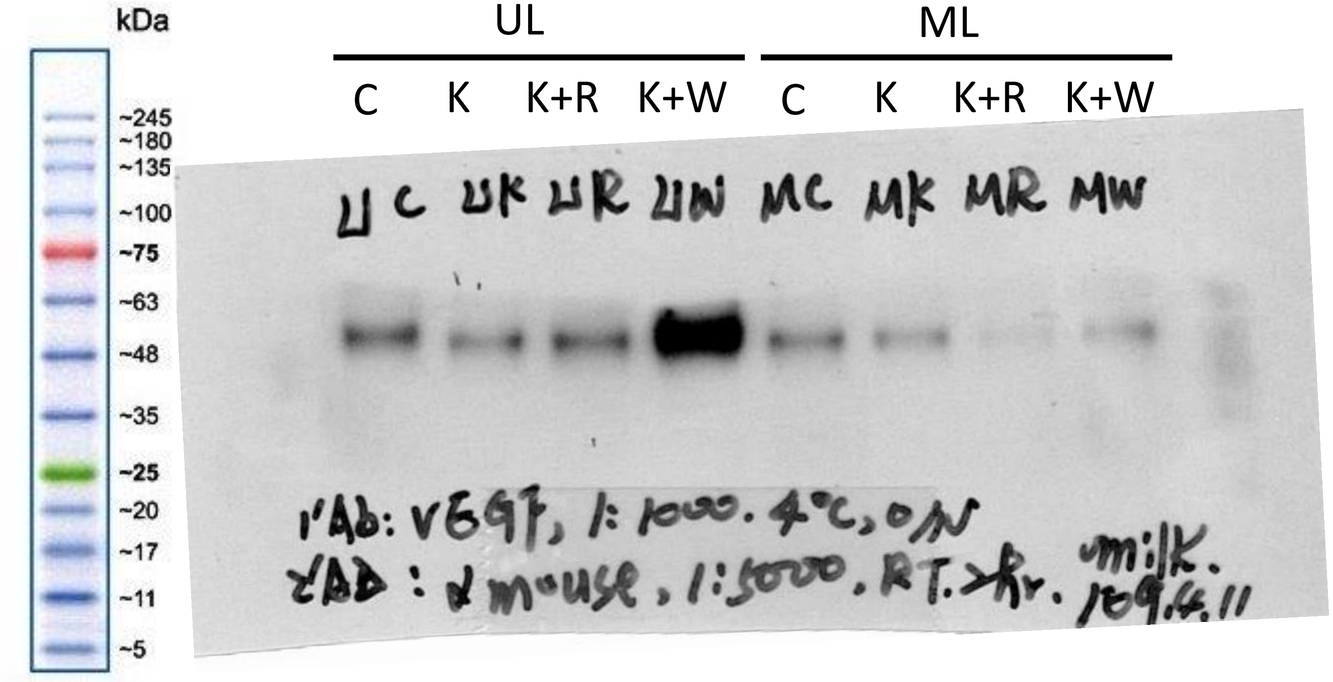

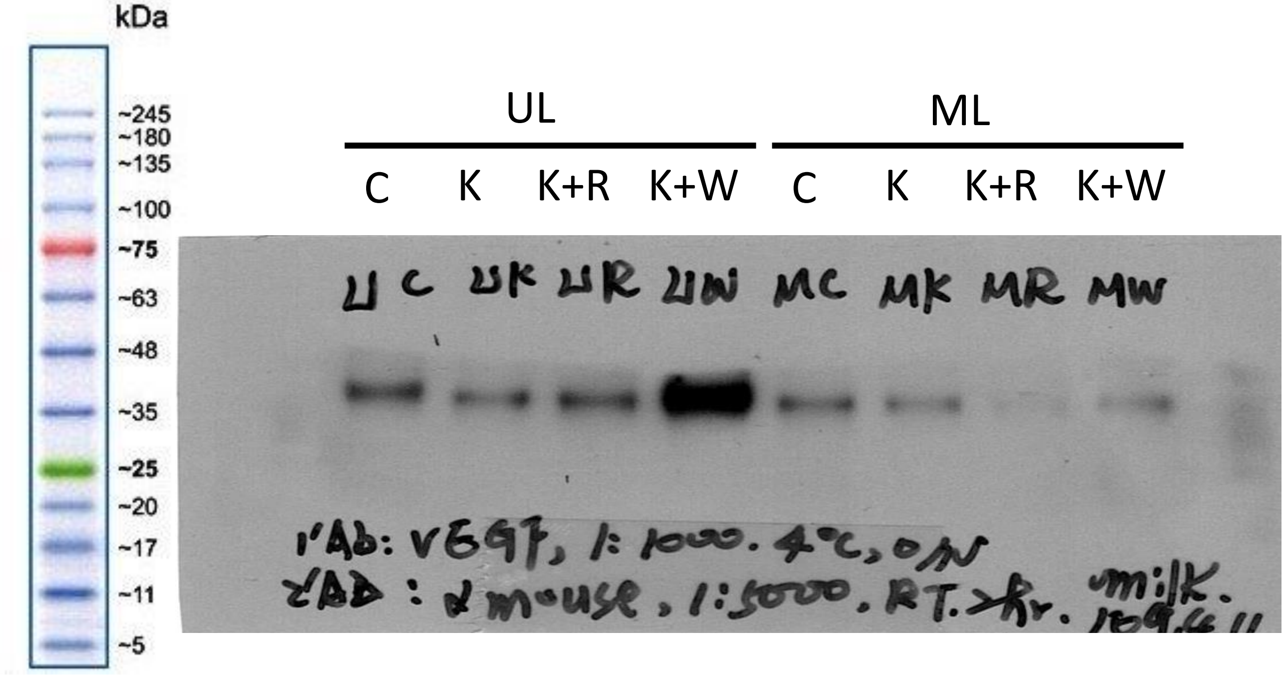

Western blot

Protein: VEGF-R1

Catalog number: no.ab32152

Working concentration: 1:1000

1^0 Ab: Abcam, rabbit monoclonal IgG

Molecular weight (kDa):150 kDa

Gel (%): 8%

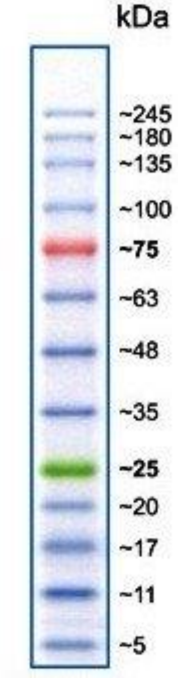

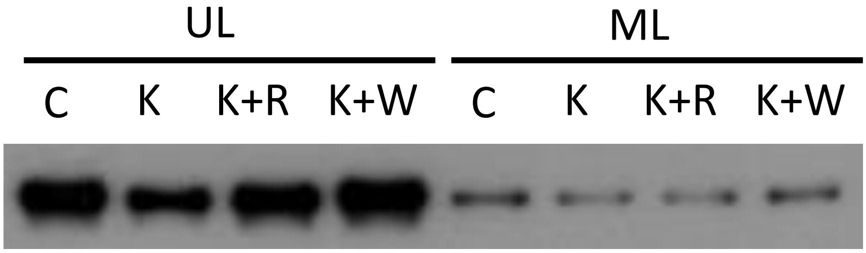

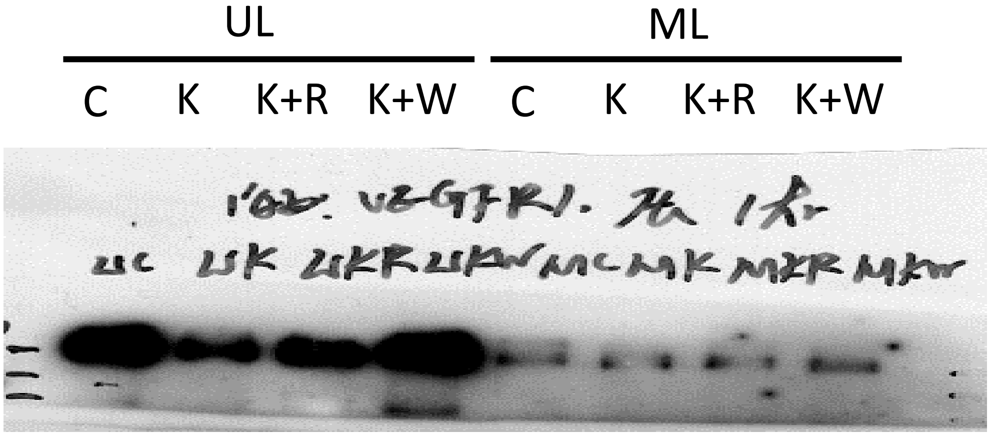

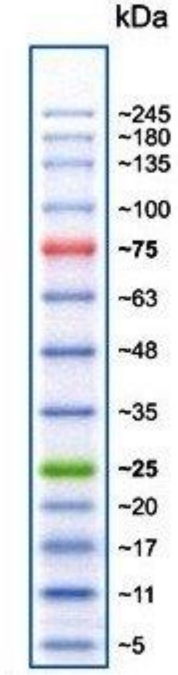

Western blot

Protein: VEGF-R1

1^0 Ab: Abcam, rabbit monoclonal IgG

Catalog number: no.ab32152

Molecular weight (kDa):150 kDa

Working concentration: 1:1000

Gel (%): 8%

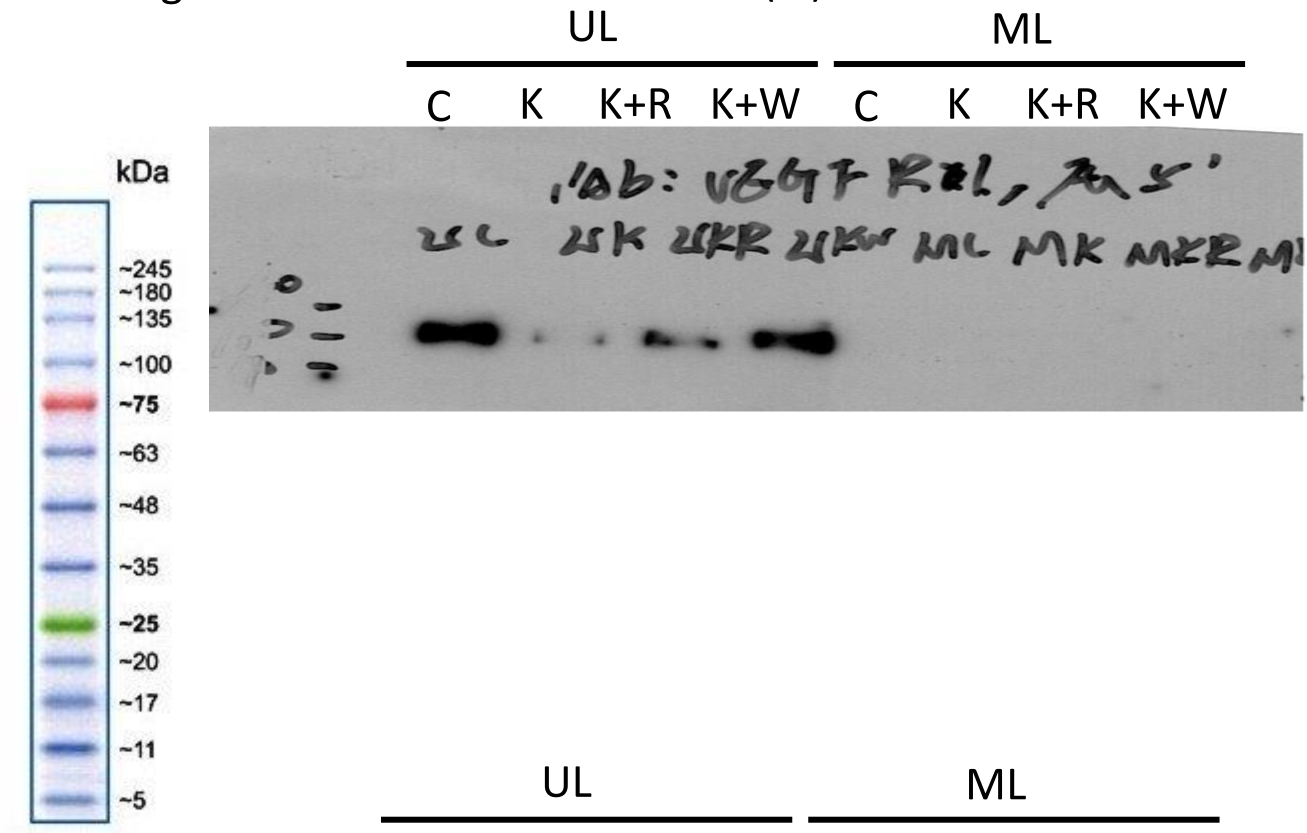

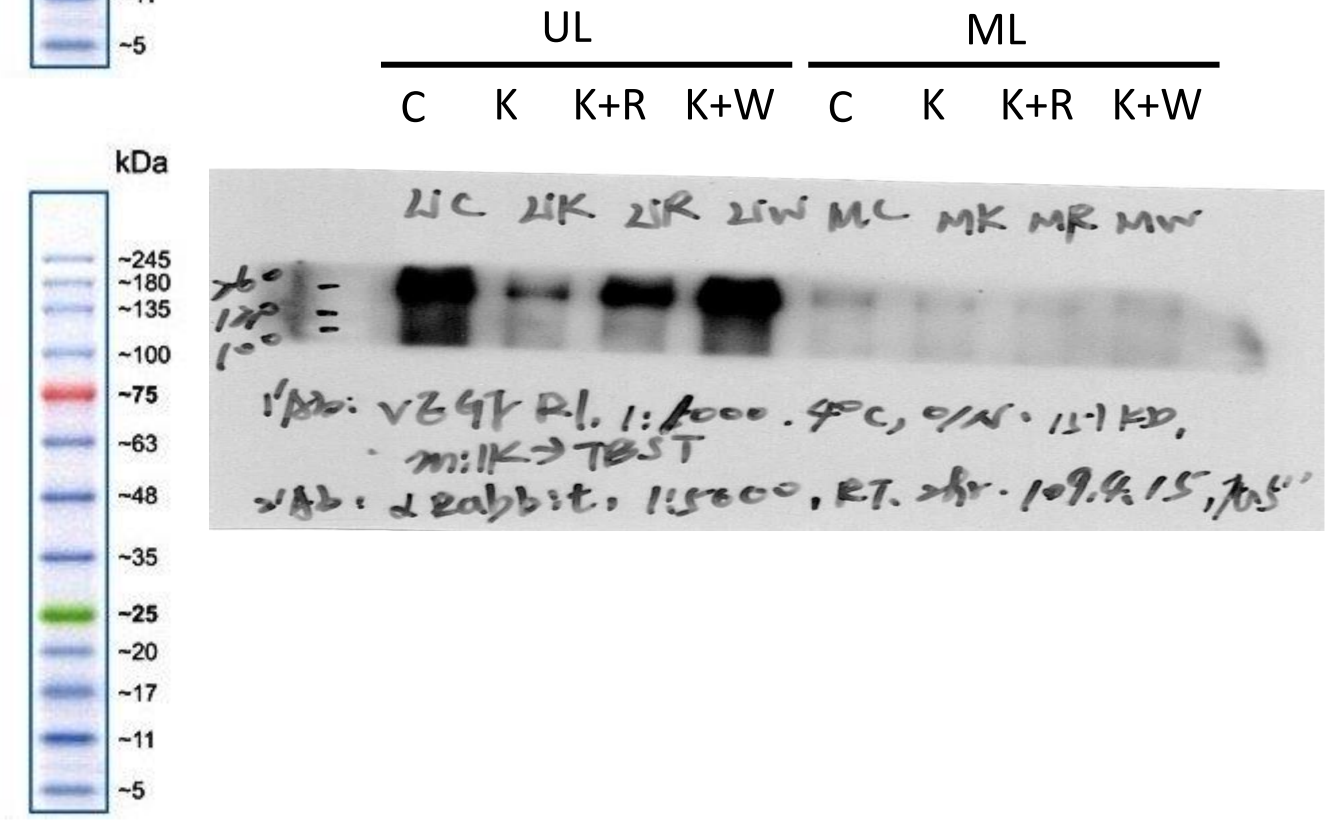

Western blot

Protein: VEGF-R2

1^0 Ab: Cell Signaling, rabbit monoclonal IgG

Catalog number: no.#9698

Molecular weight (kDa):220 kDa

Working concentration: 1:1000

Gel (%): 8%

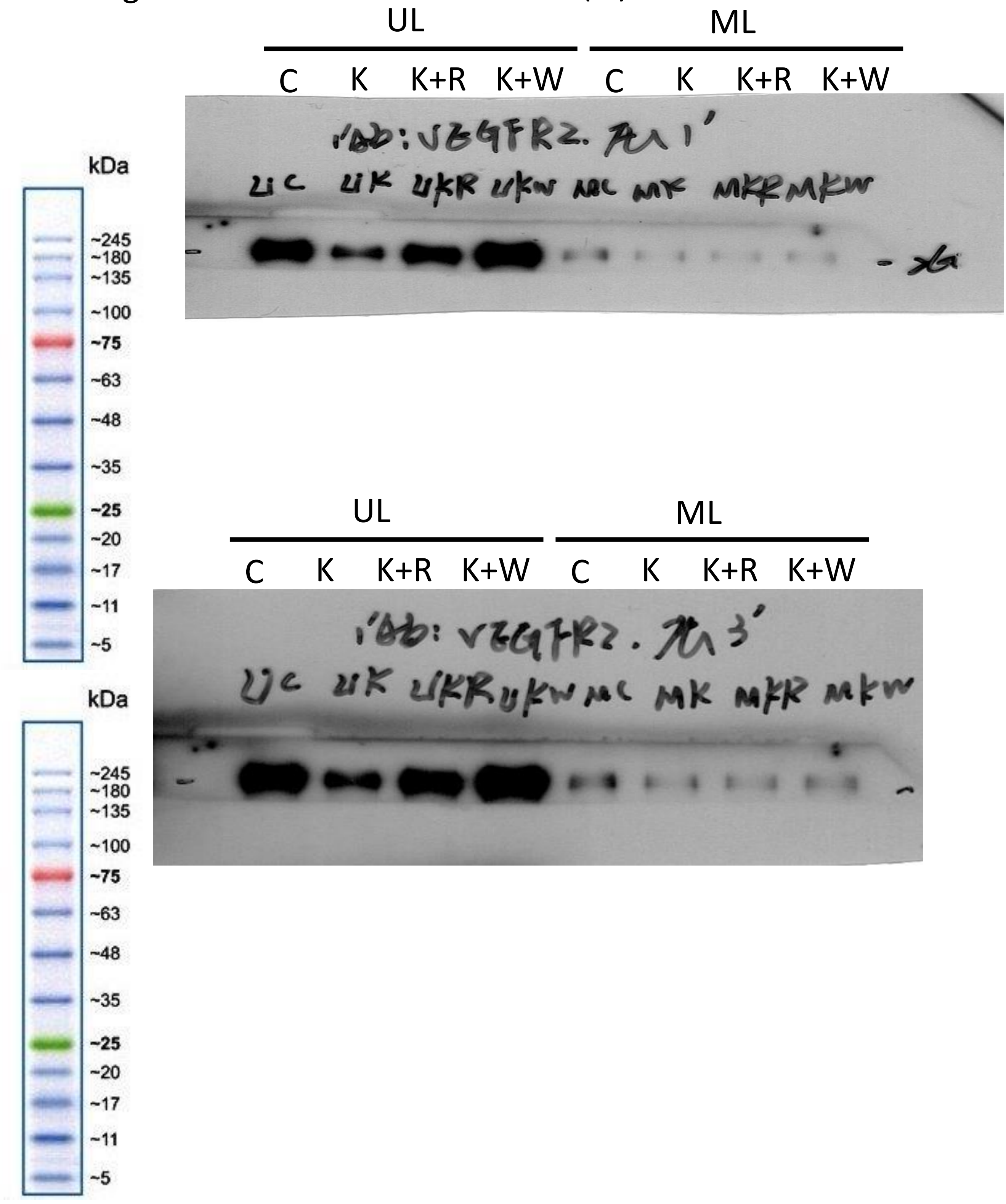

Western blot

Protein: VEGF-R2

1^0 Ab: Cell Signaling, rabbit monoclonal IgG

Catalog number: no.#9698

Molecular weight (kDa):220 kDa

Working concentration: 1:1000

Gel (%): 8%

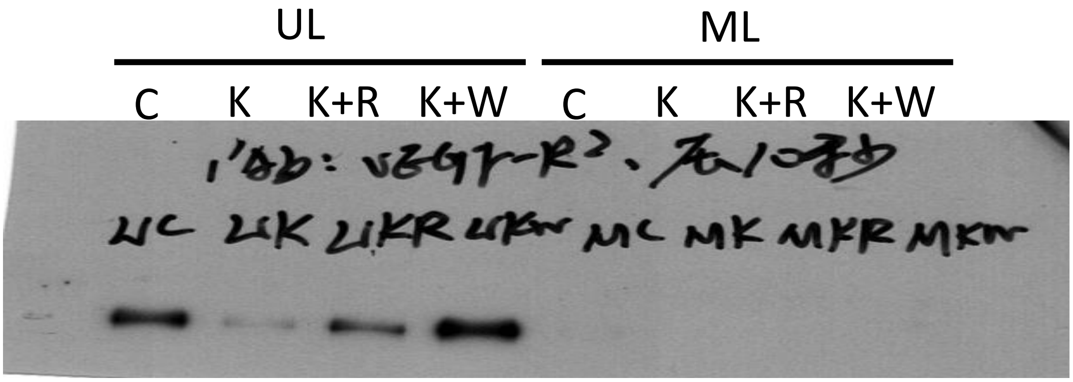

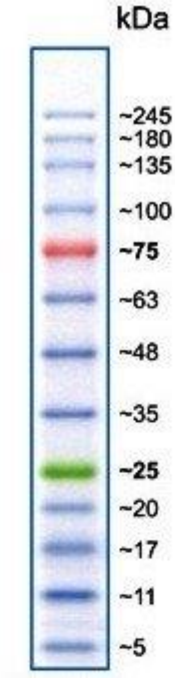

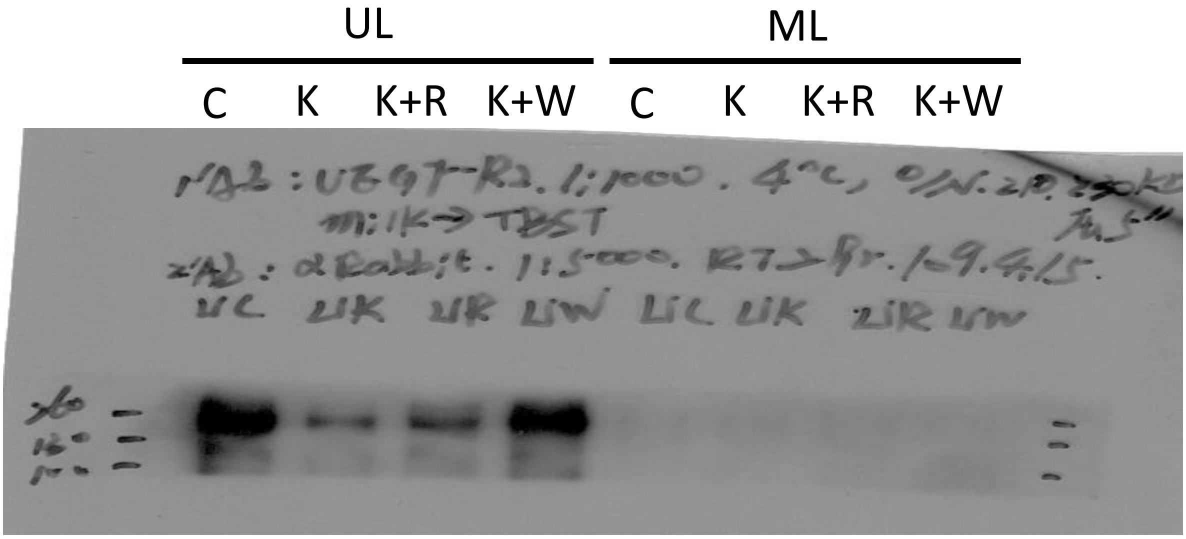

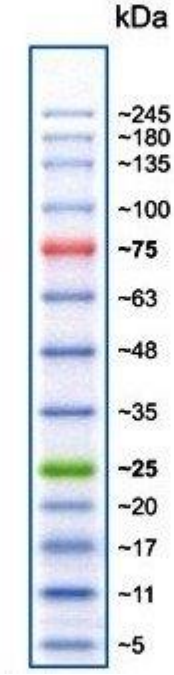

Western blot

Protein: Laminin

Catalog number: no.ab11575

Working concentration: 1:1000

1^0 Ab: Abcam, rabbit monoclonal IgG

Molecular weight (kDa): 200~400 kDa

Gel (%): 8%

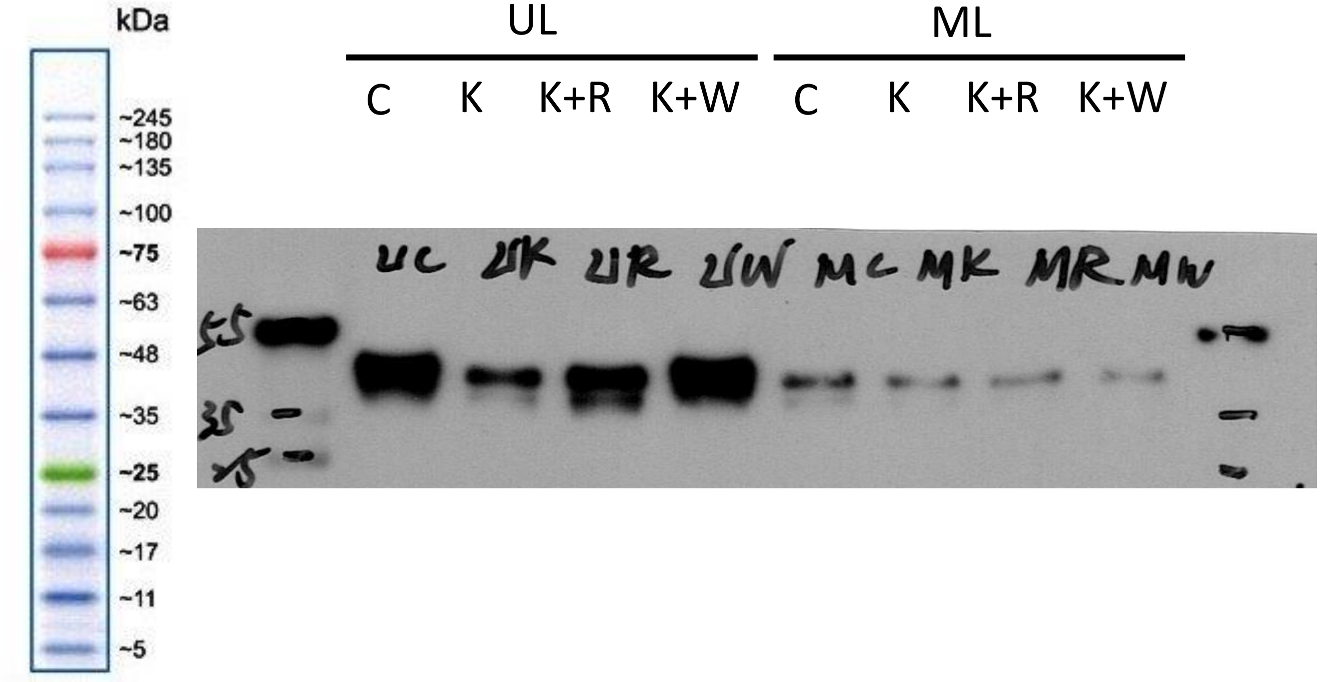

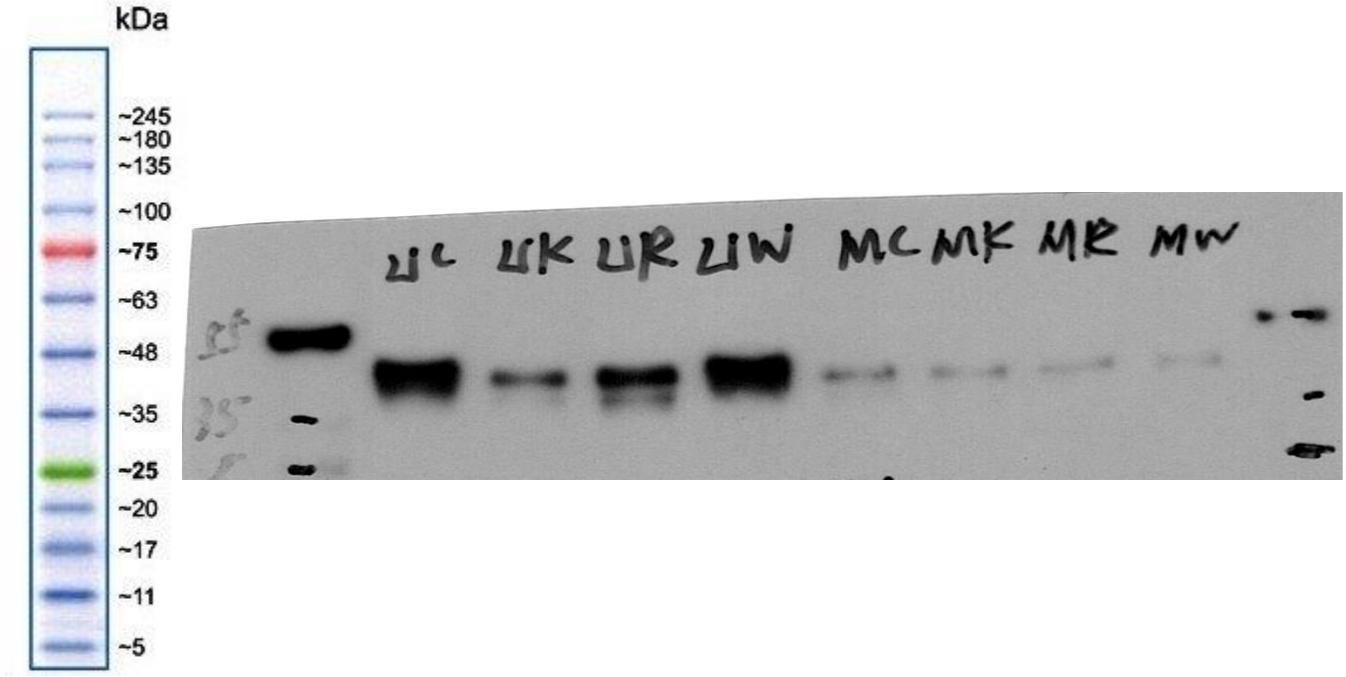

Western blot

Protein: Laminin

Catalog number: no.ab11575

Working concentration: 1:1000

1^{0} Ab: Abcam, rabbit monoclonal IgG

Molecular weight (kDa): 200~400 kDa

Gel (%): 8%

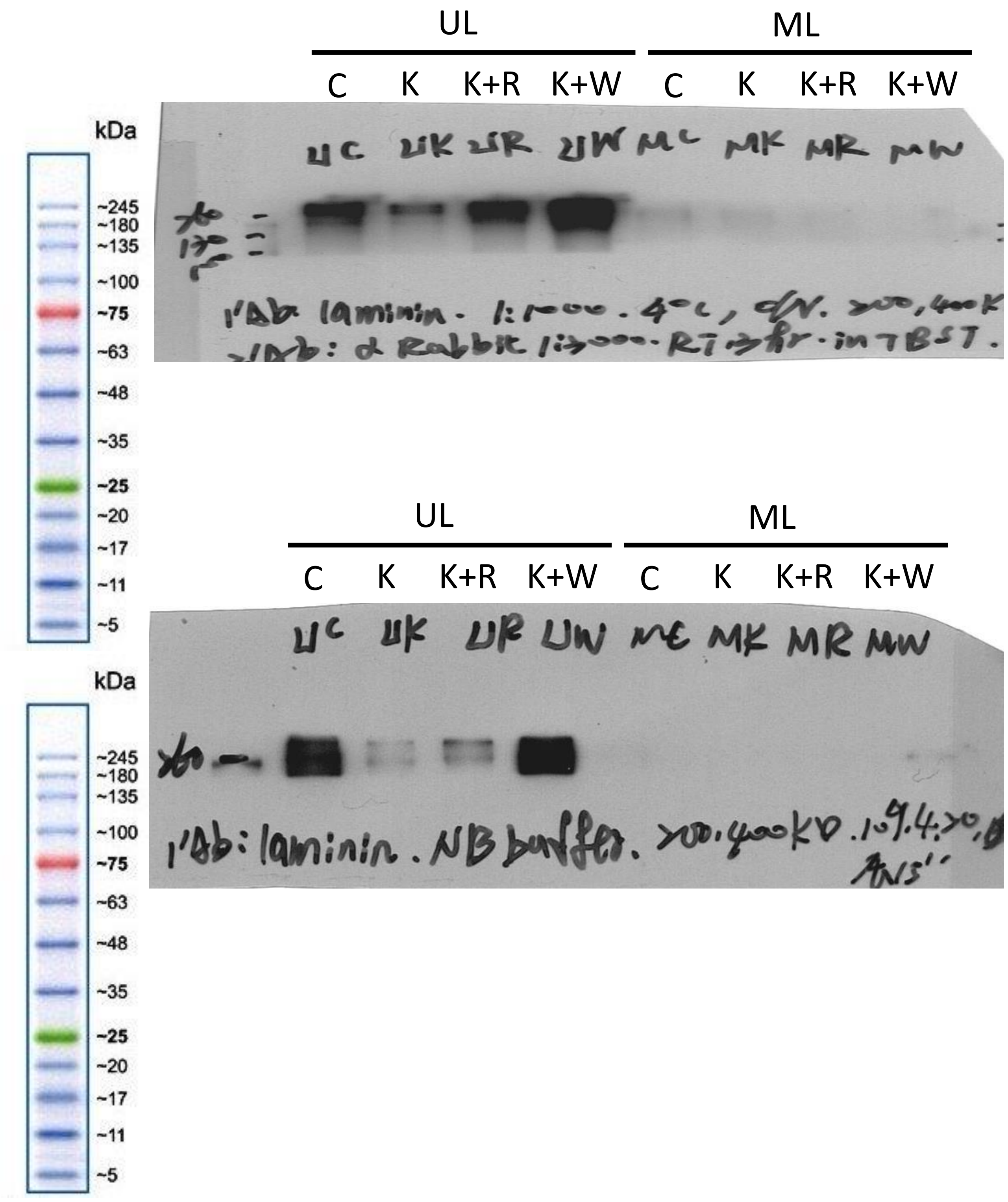

Western blot

Protein: Laminin

Catalog number: no.ab11575

Working concentration: 1:1000

1^0 Ab: Abcam, rabbit monoclonal IgG

Molecular weight (kDa): 200~400 kDa

Gel (%): 8%

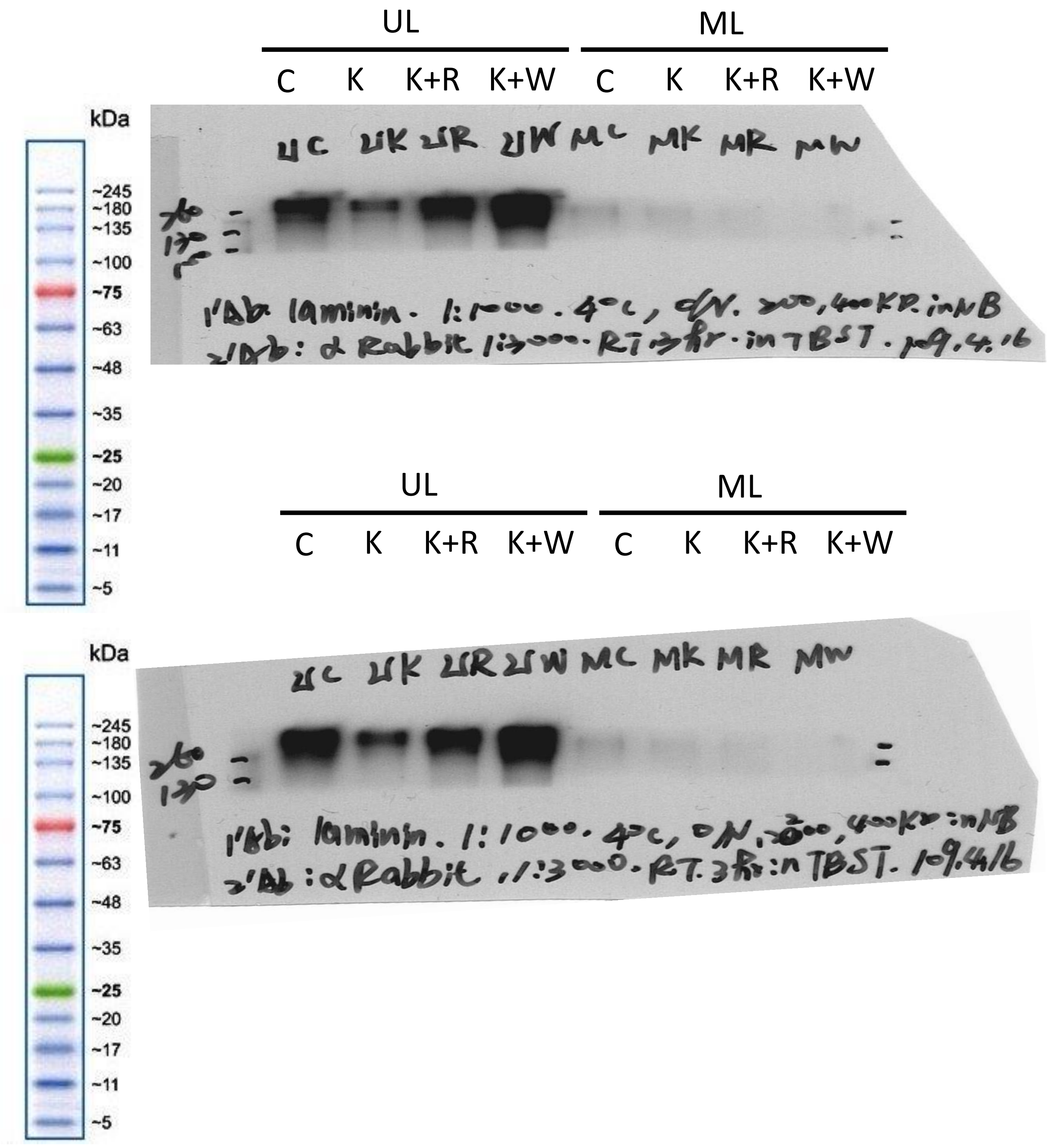

Western blot

Protein: Integrin-α6

1^0 Ab: Abcam, rabbit monoclonal IgG

Catalog number: no.ab181551

Molecular weight (kDa):127 kDa

Working concentration: 1:5000

Gel (%): 8%

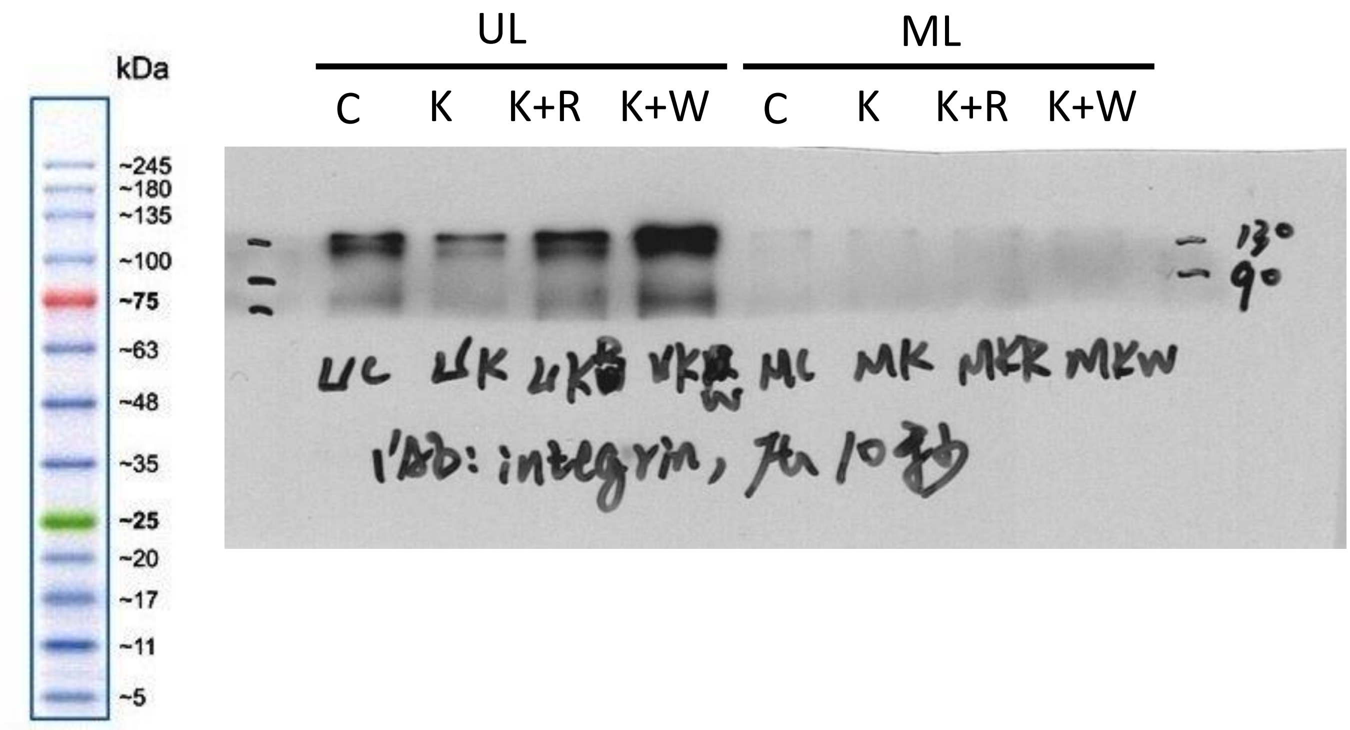

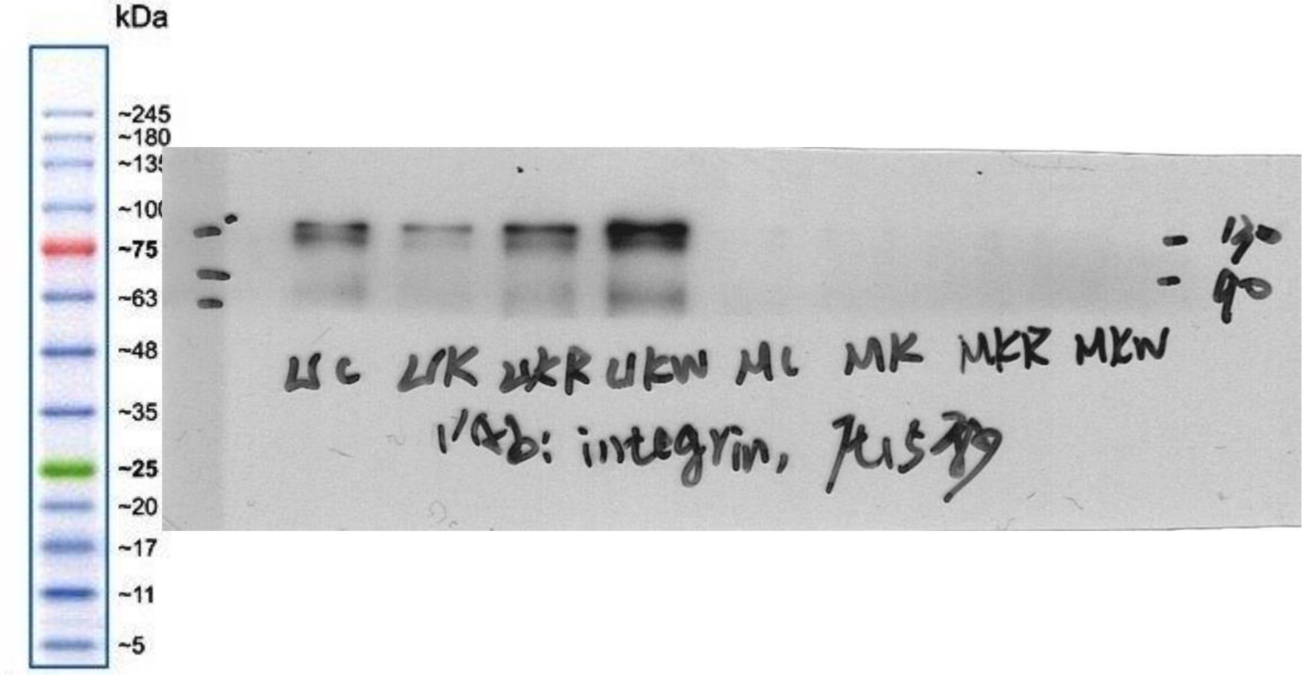

Western blot

Protein: Integrin-α6

Catalog number: no.ab181551

Working concentration: 1:5000

1^0 **Ab:** Abcam, rabbit monoclonal IgG

Molecular weight (kDa):127 kDa

Gel (%): 8%

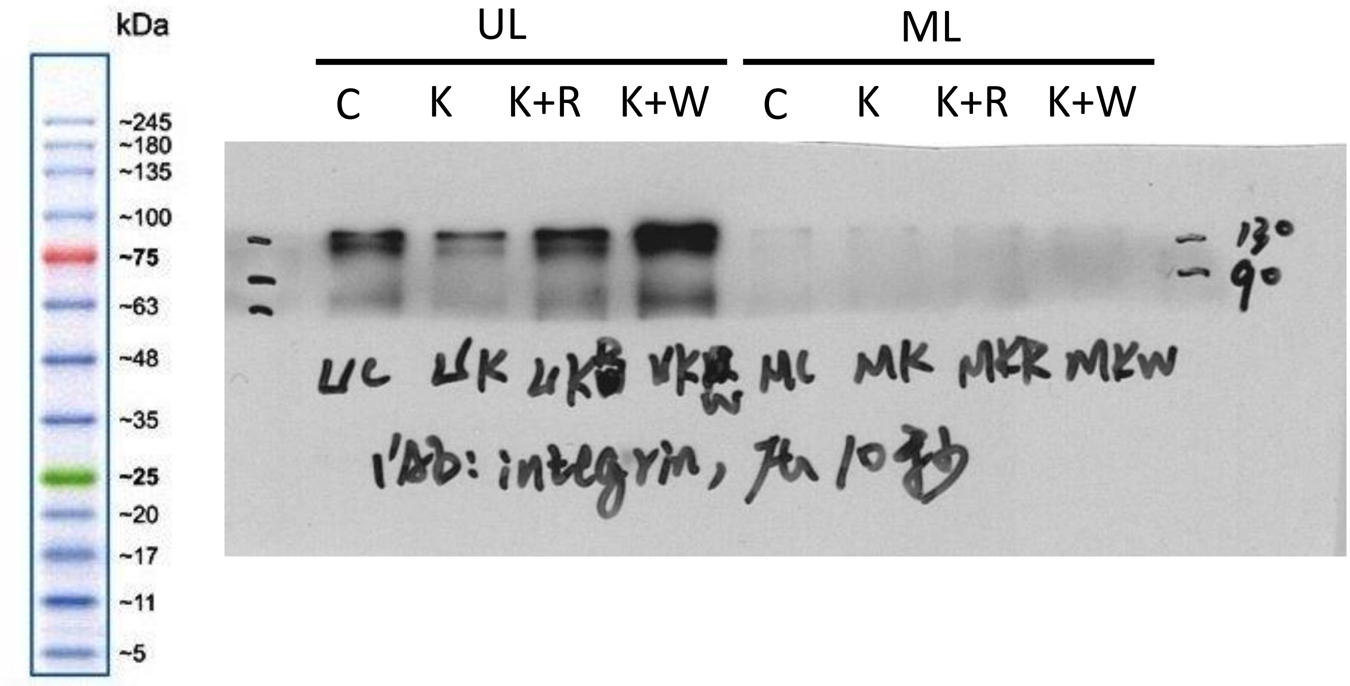

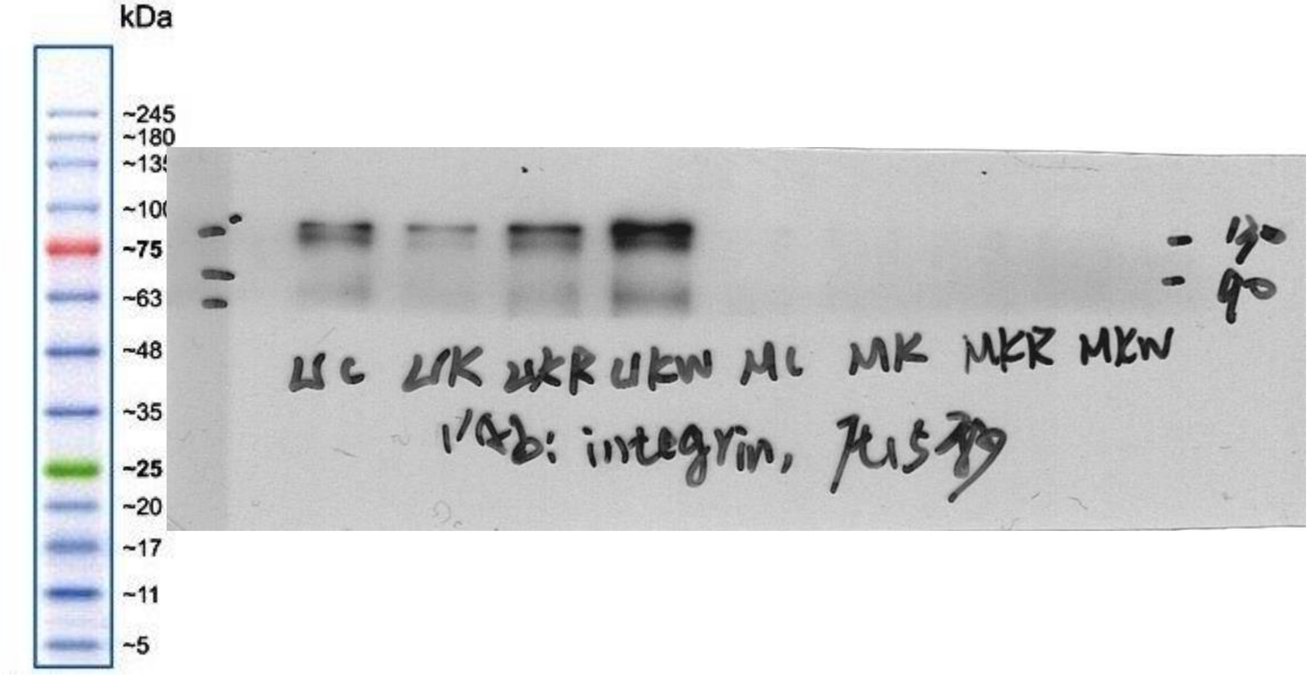

Western blot

Protein: Integrin-α6

Catalog number: no.ab181551

Working concentration: 1:5000

1⁰ Ab: Abcam, rabbit monoclonal IgG

Molecular weight (kDa):127 kDa

Gel (%): 8%

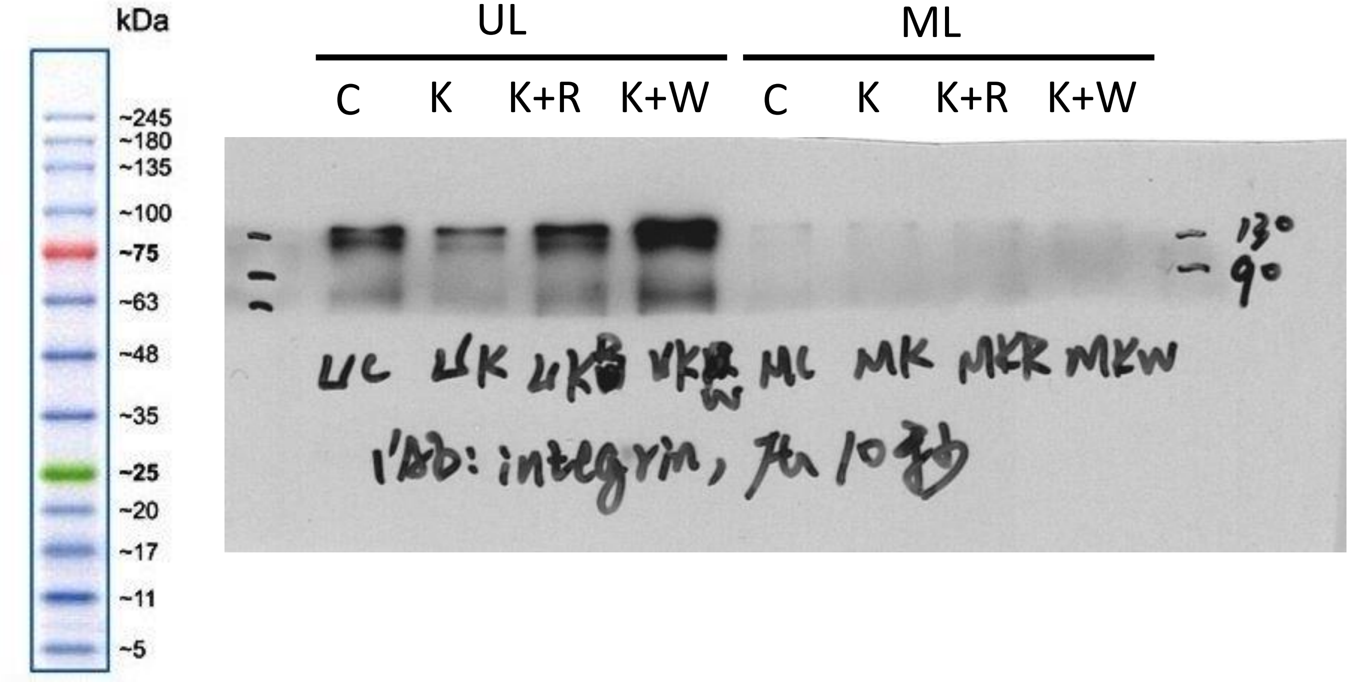

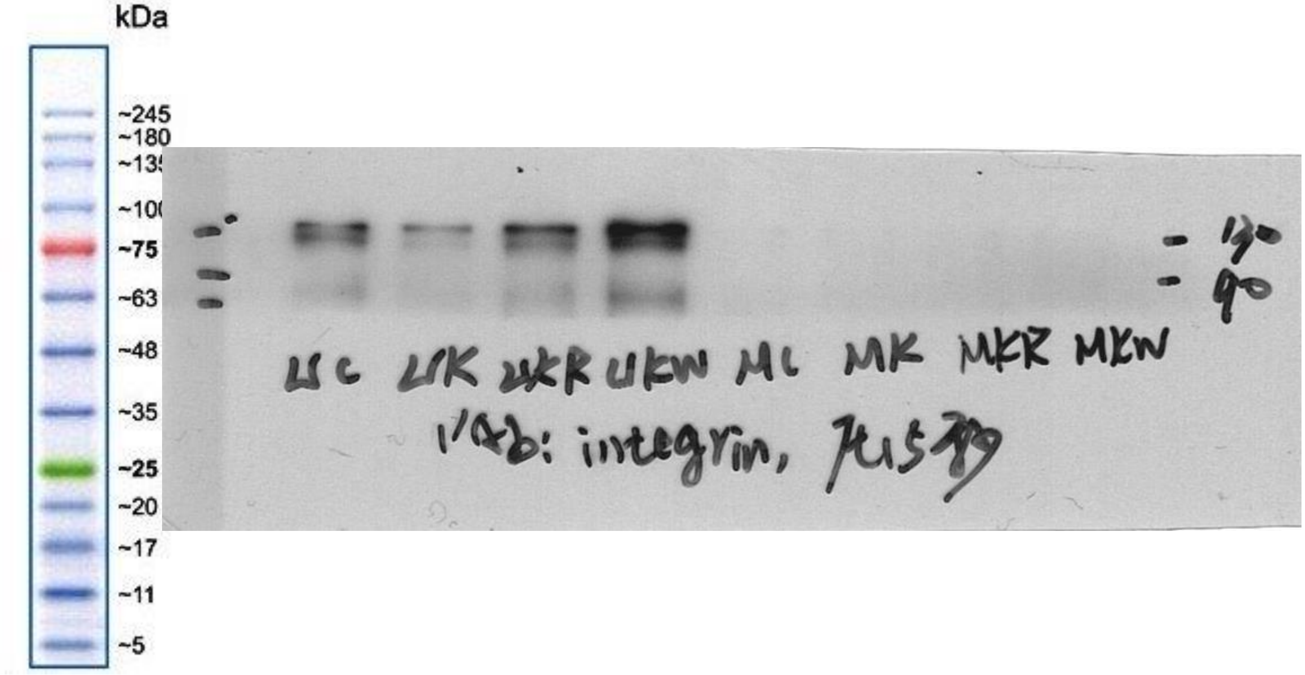

Western blot

Protein: GAPDH

1^0 Ab: Millipore, mouse monoclonal IgG1

Catalog number: no. MAB374

Molecular weight (kDa):36 kDa

Working concentration: 1:1000

Gel (%): 8%

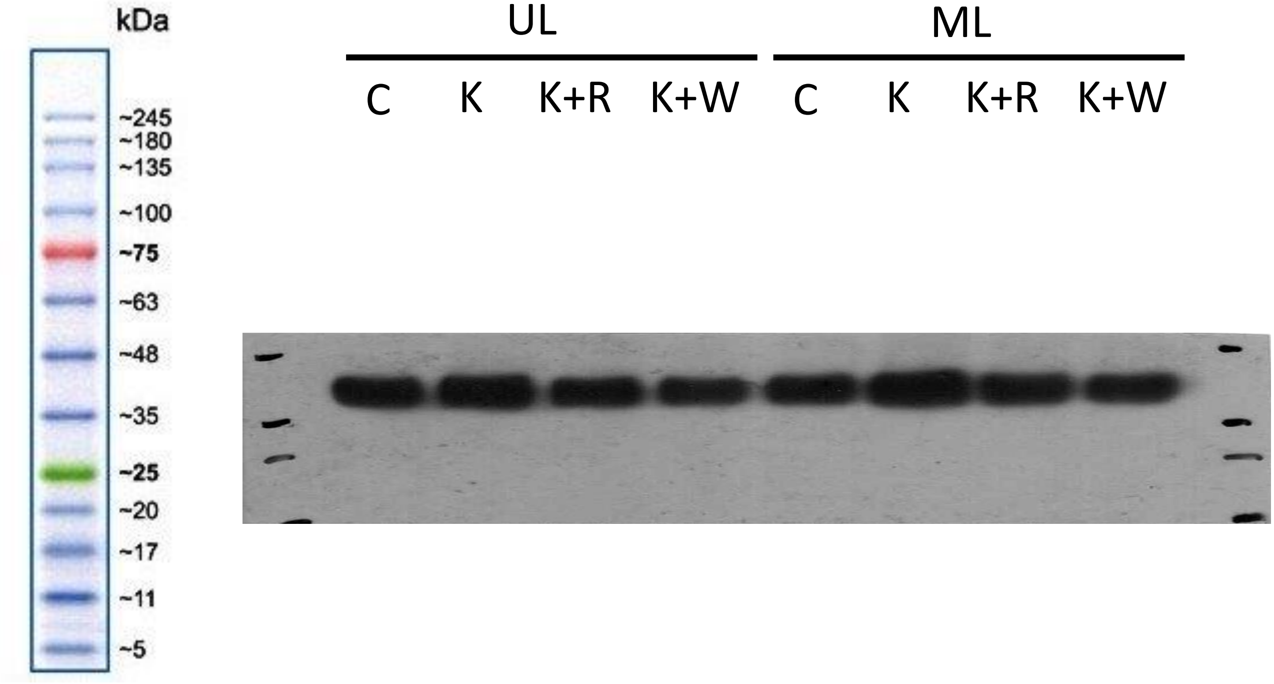

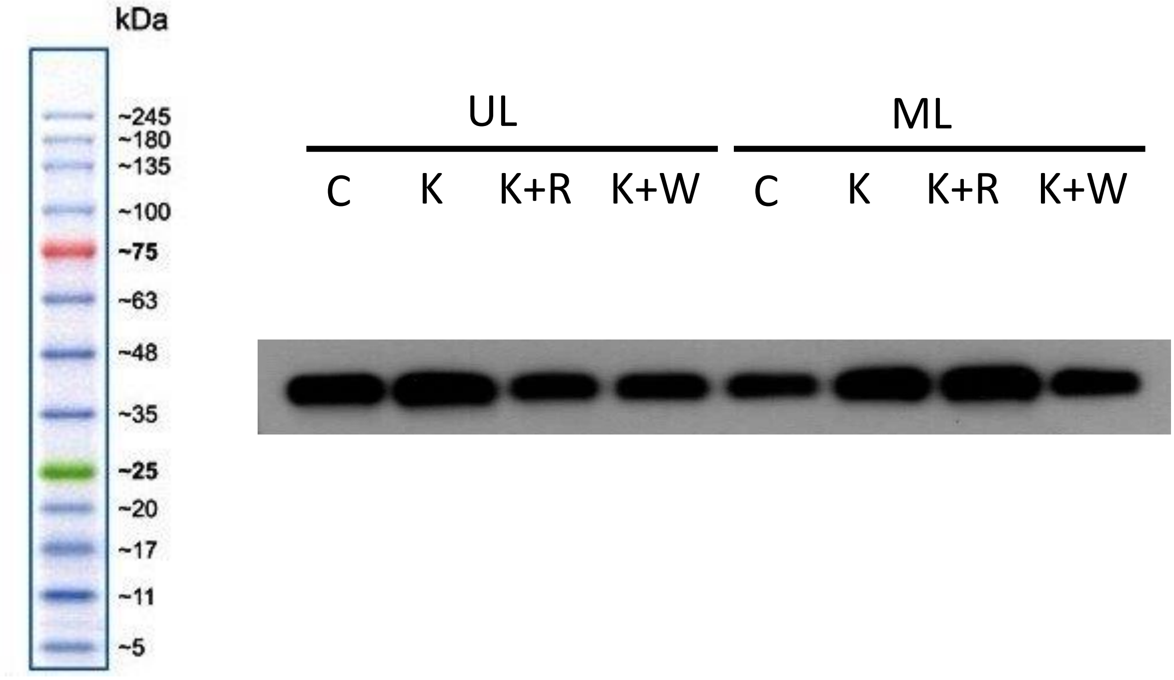

Western blot

Protein: Erk1/2 (p44/42) **1^0 Ab:** Cell Signaling, rabbit monoclonal IgG

Catalog number: no.#9102

Molecular weight (kDa):42~44 kDa

Working concentration: 1:1000

Gel (%): 8%

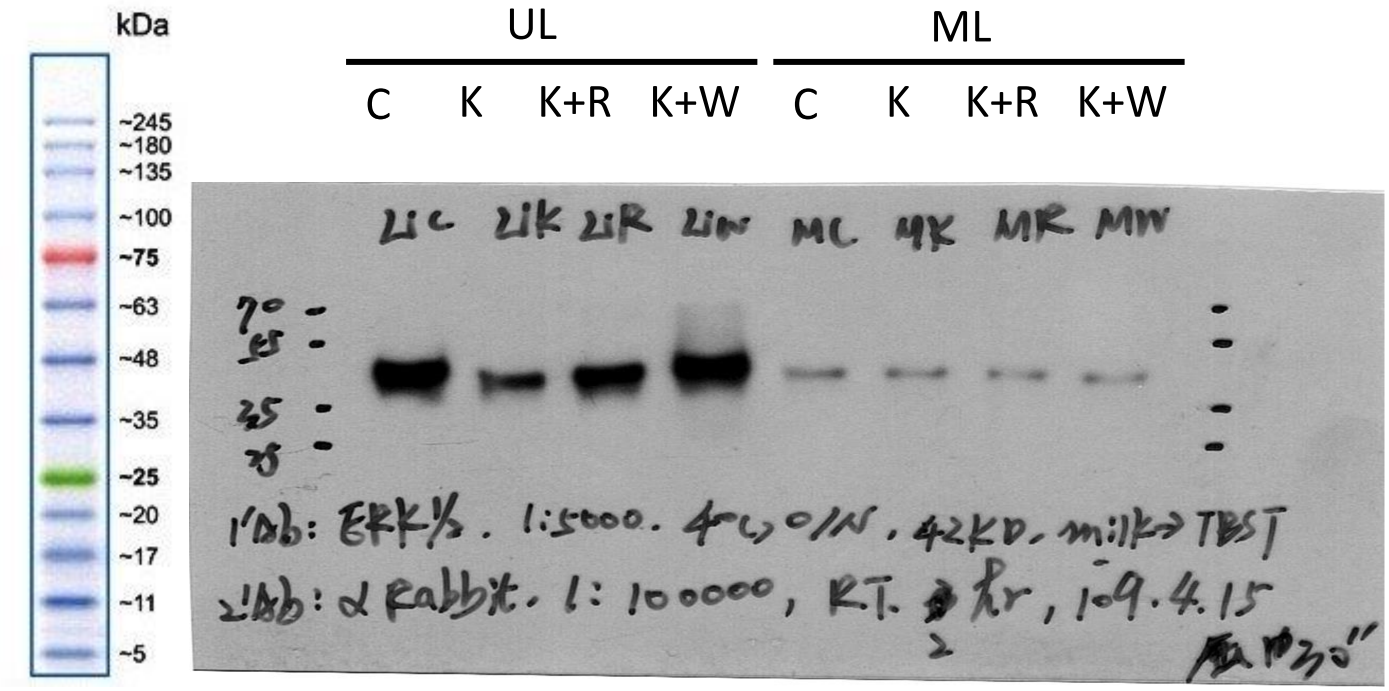

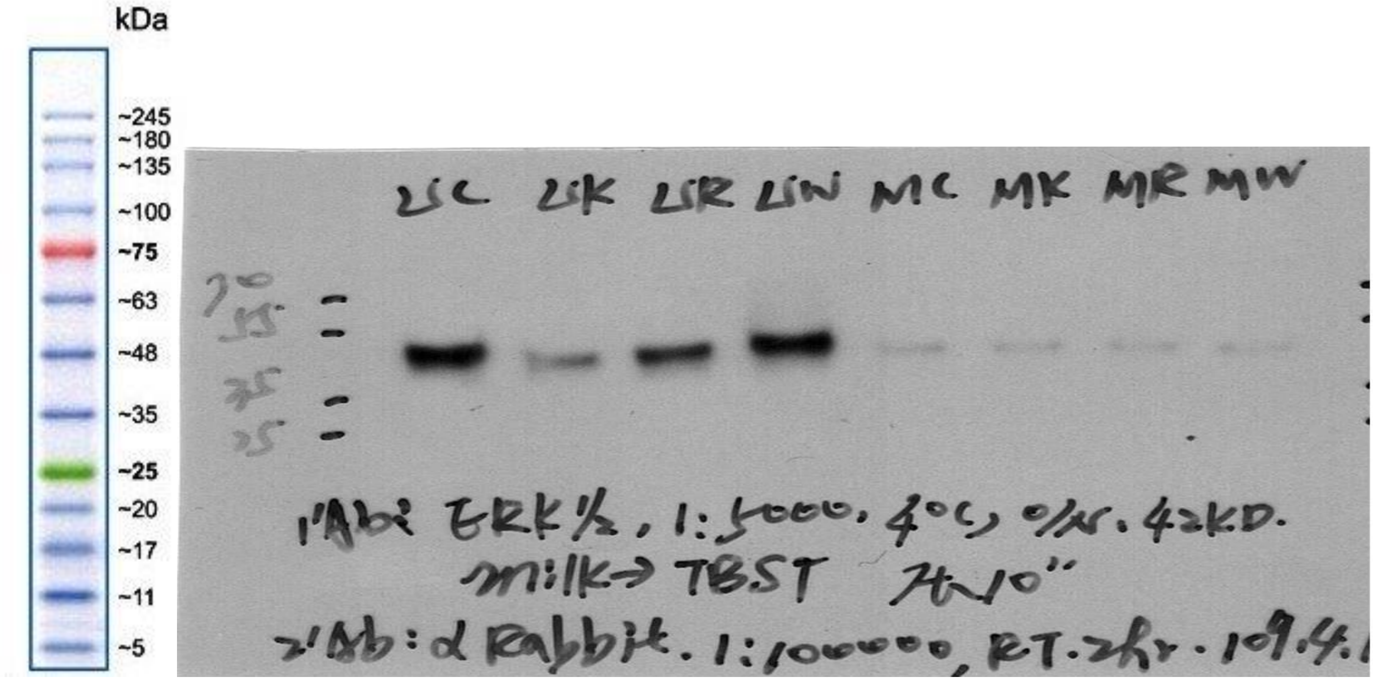

Western blot

Protein: Erk1/2 (p44/42) **1^0 Ab:** Cell Signaling, rabbit monoclonal IgG

Catalog number: no.#9102

Molecular weight (kDa):42~44 kDa

Working concentration: 1:1000

Gel (%): 8%

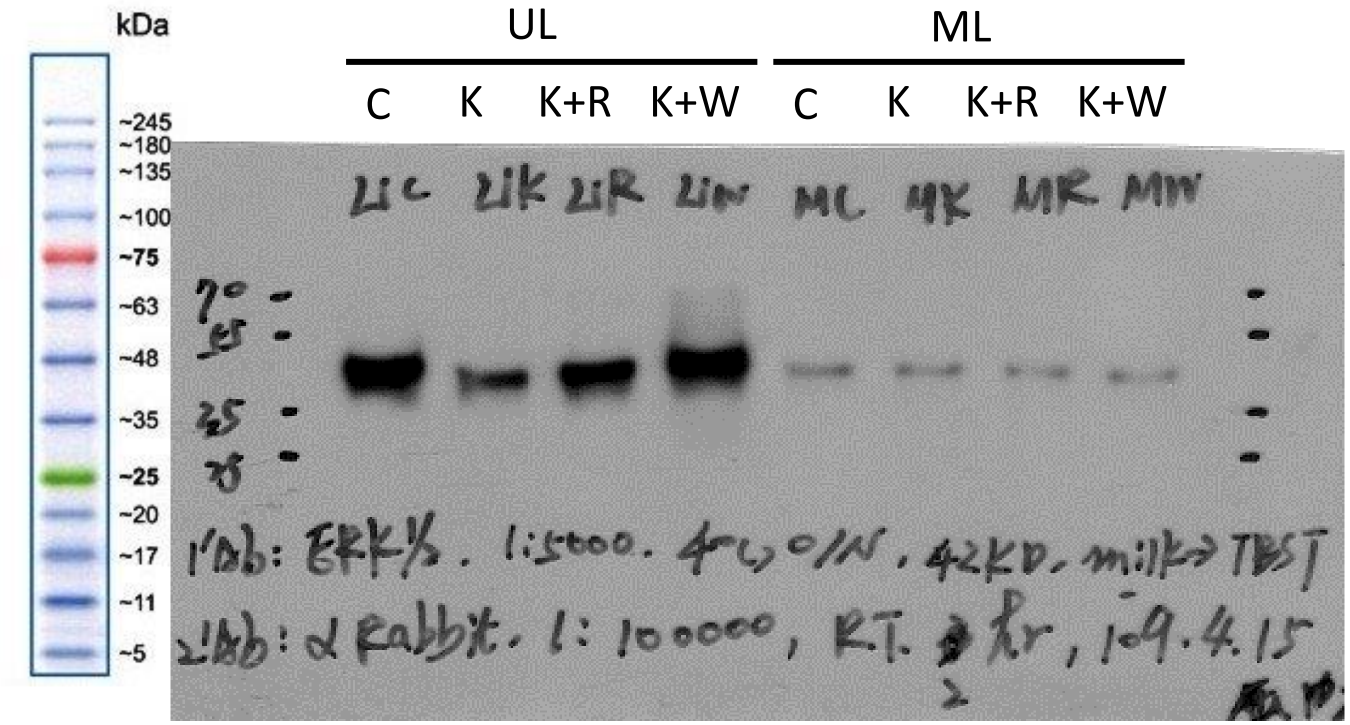

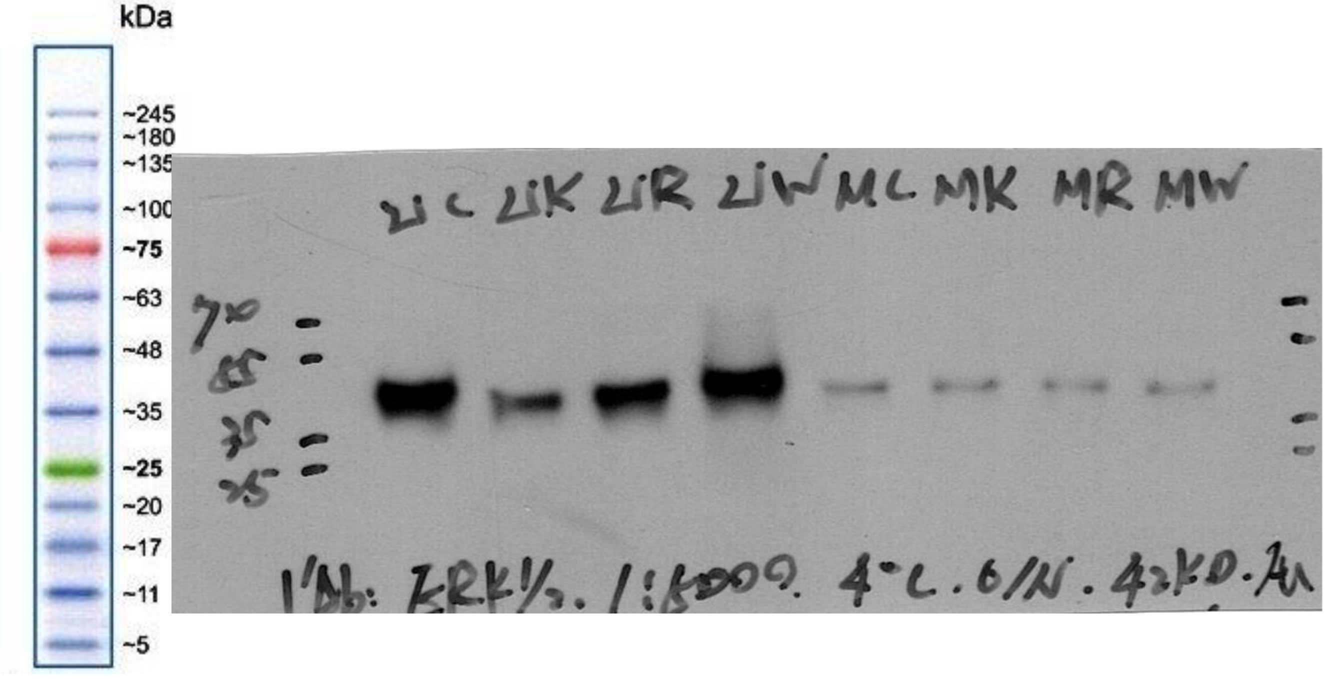

Western blot

Protein: Erk1/2 (p44/42) **1^0 Ab:** Cell Signaling, rabbit monoclonal IgG

Catalog number: no.#9102 Molecular weight (kDa):42~44 kDa

Working concentration: 1:1000 Gel (%): 8%

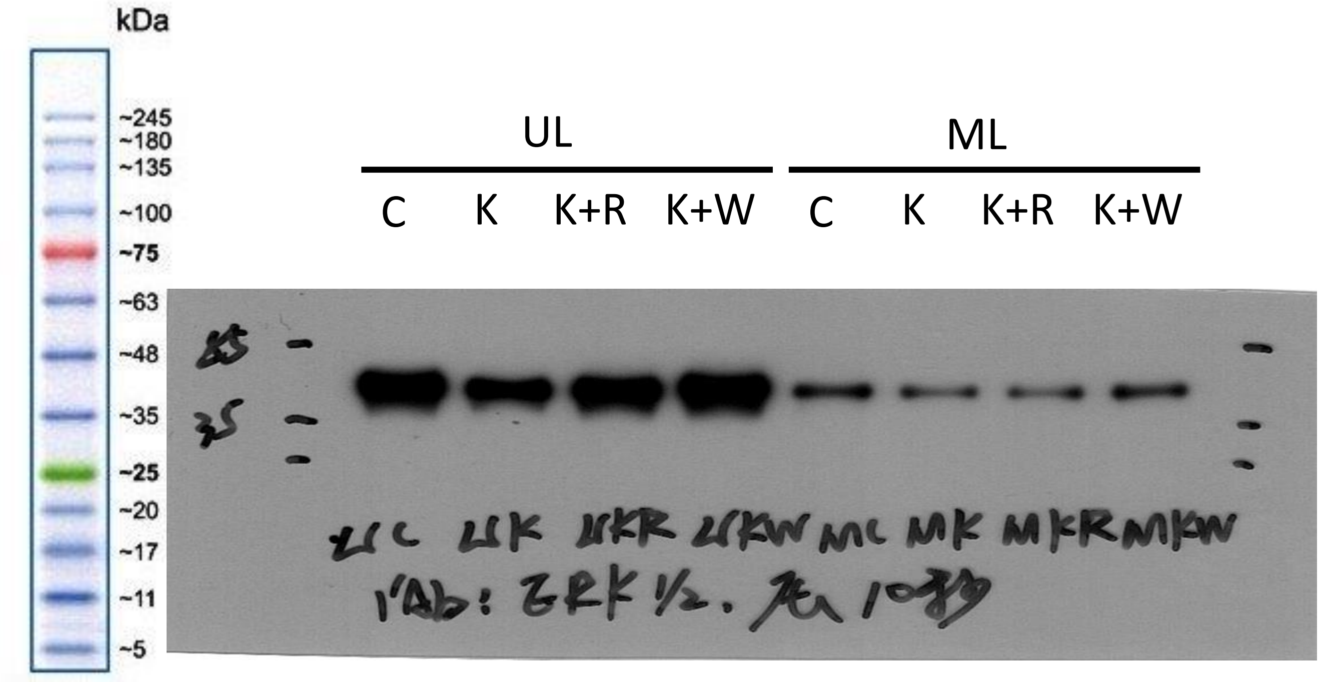

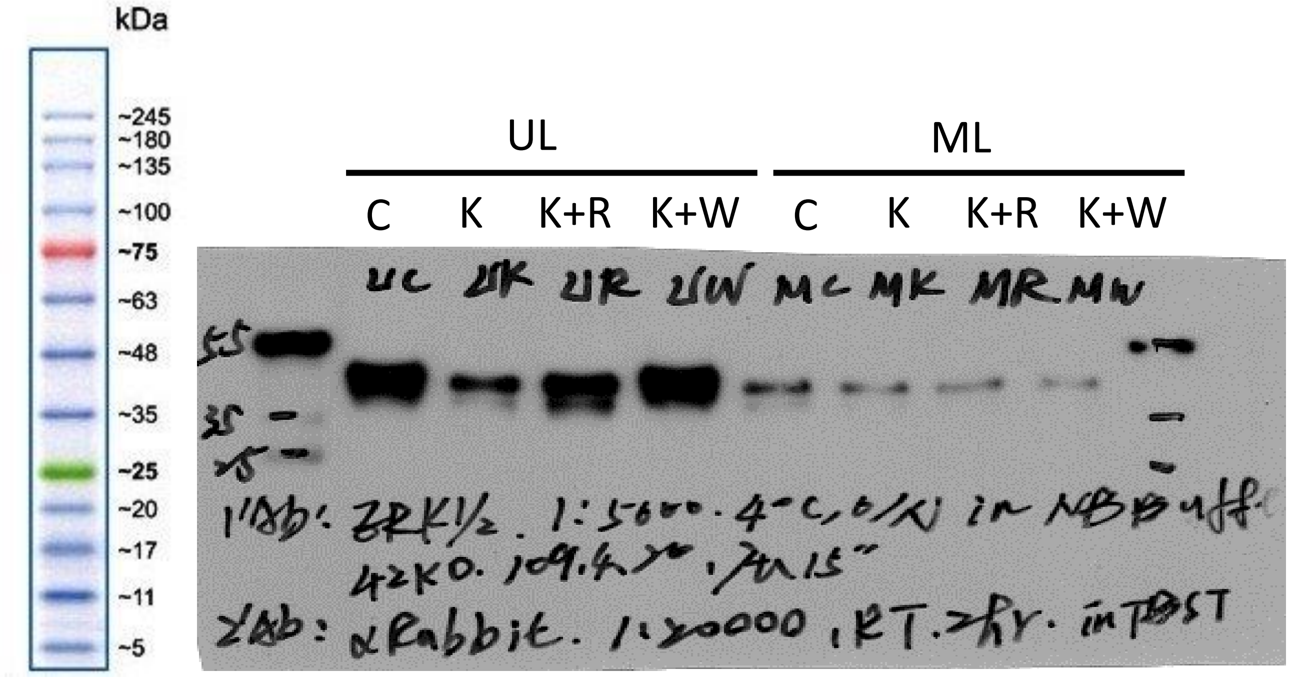

Western blot

Protein: Erk1/2 (p44/42) **1^0 Ab:** Cell Signaling, rabbit monoclonal IgG

Catalog number: no.#9102 Molecular weight (kDa):42~44 kDa

Working concentration: 1:1000 Gel (%): 8%

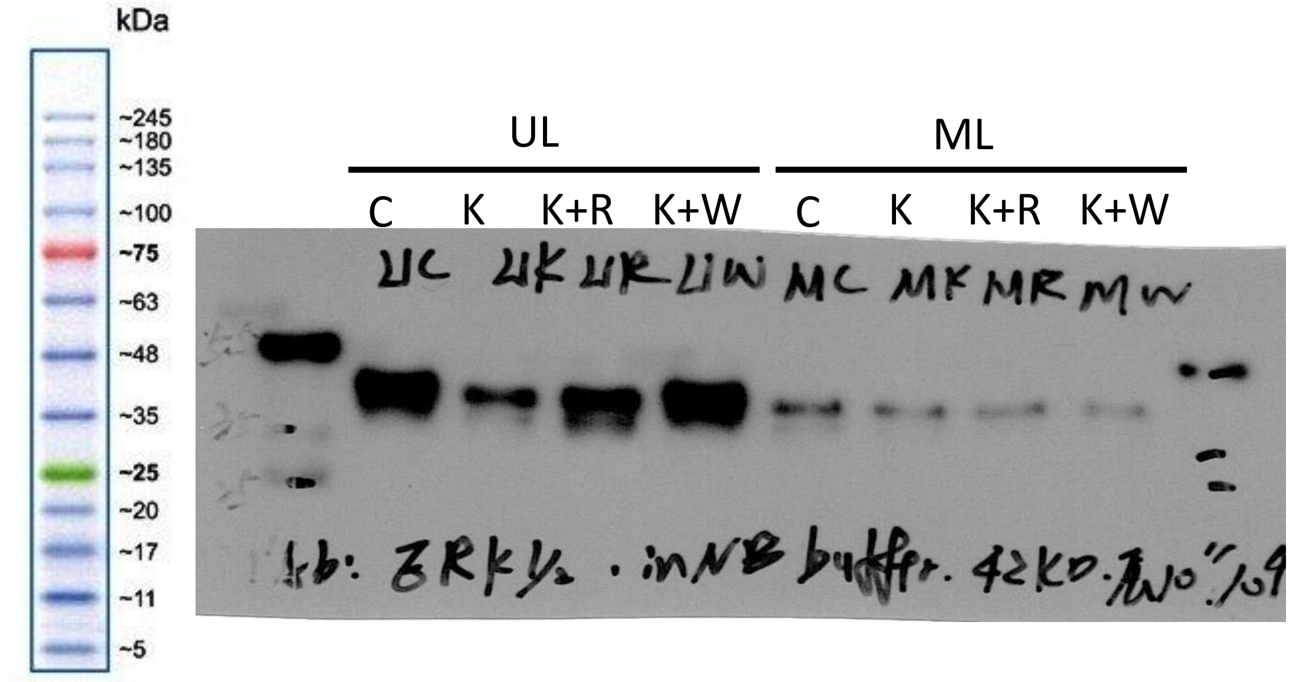

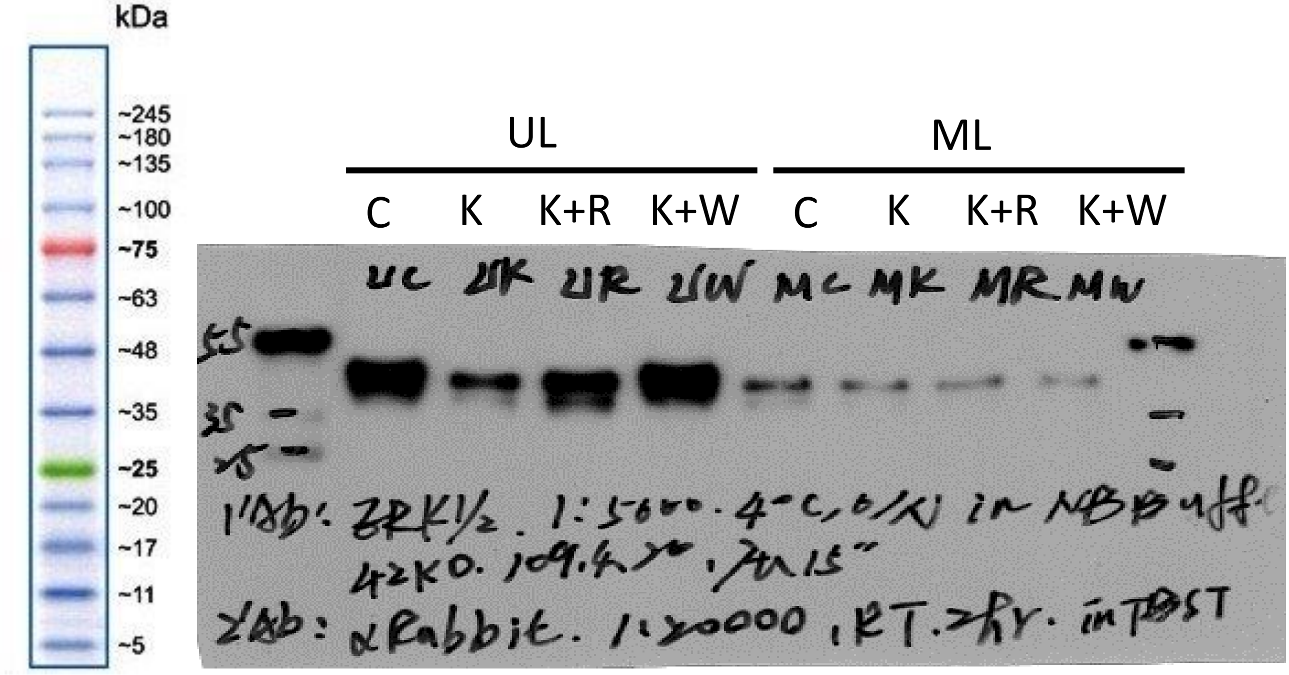

Western blot

Protein: p-Erk1/2

1^0 Ab: Cell Signaling, rabbit monoclonal IgG

Catalog number: no.#4370

Molecular weight (kDa): 42~44 kDa

Working concentration: 1:2000

Gel (%): 8%

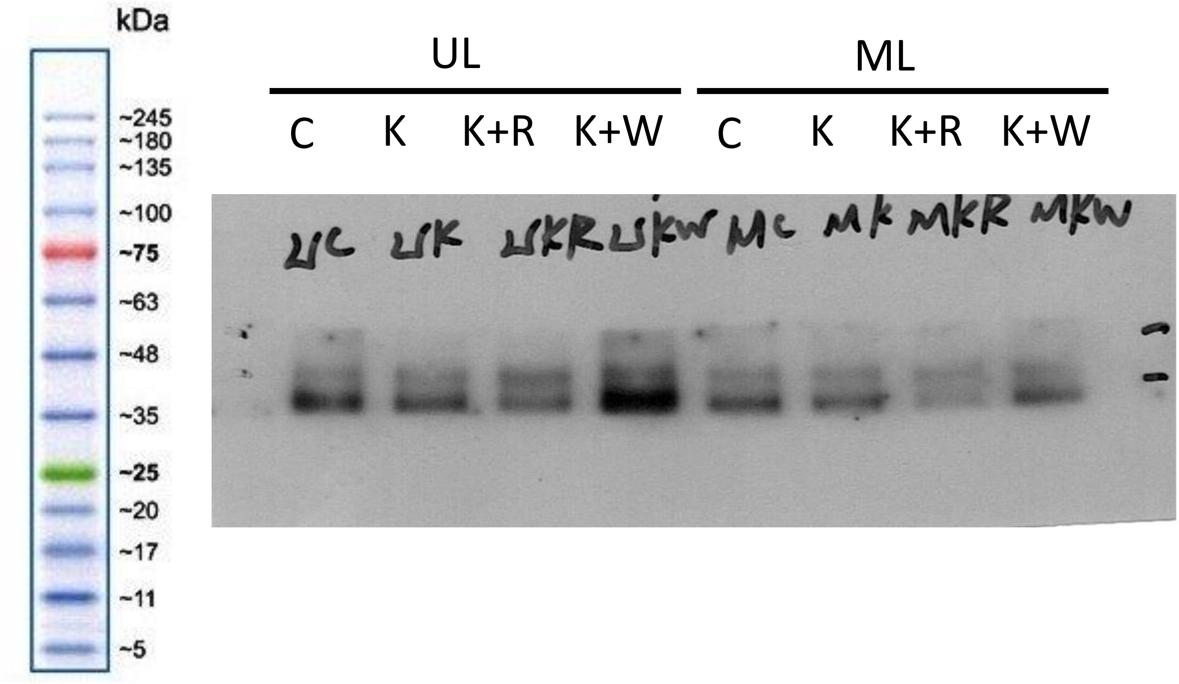

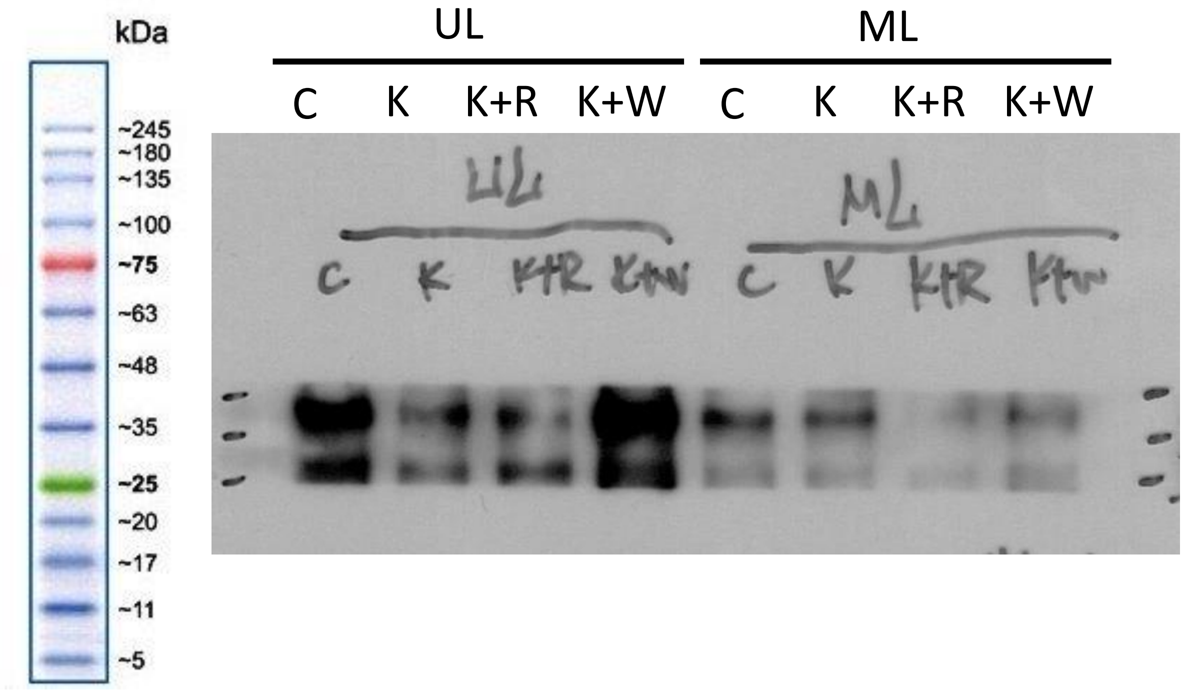

Western blot

Protein: P38

1^0 Ab: Cell Signaling, rabbit monoclonal IgG

Catalog number: no.#8690

Molecular weight (kDa): 38~40 kDa

Working concentration: 1:1000

Gel (%): 8%

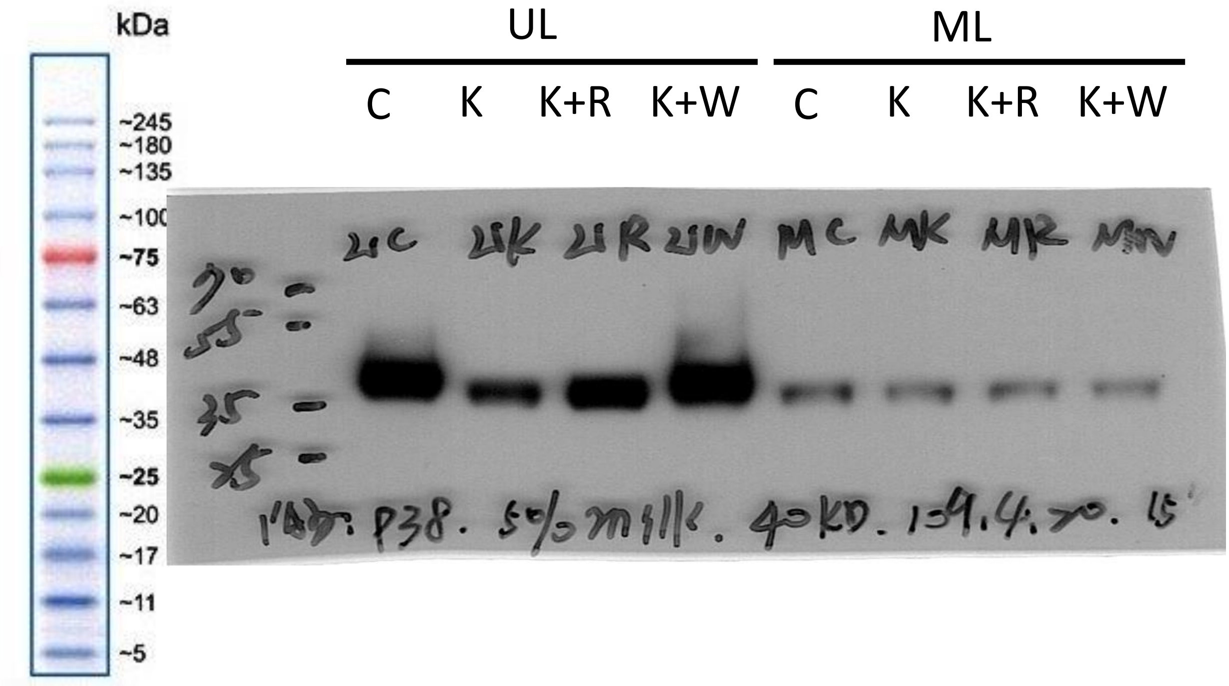

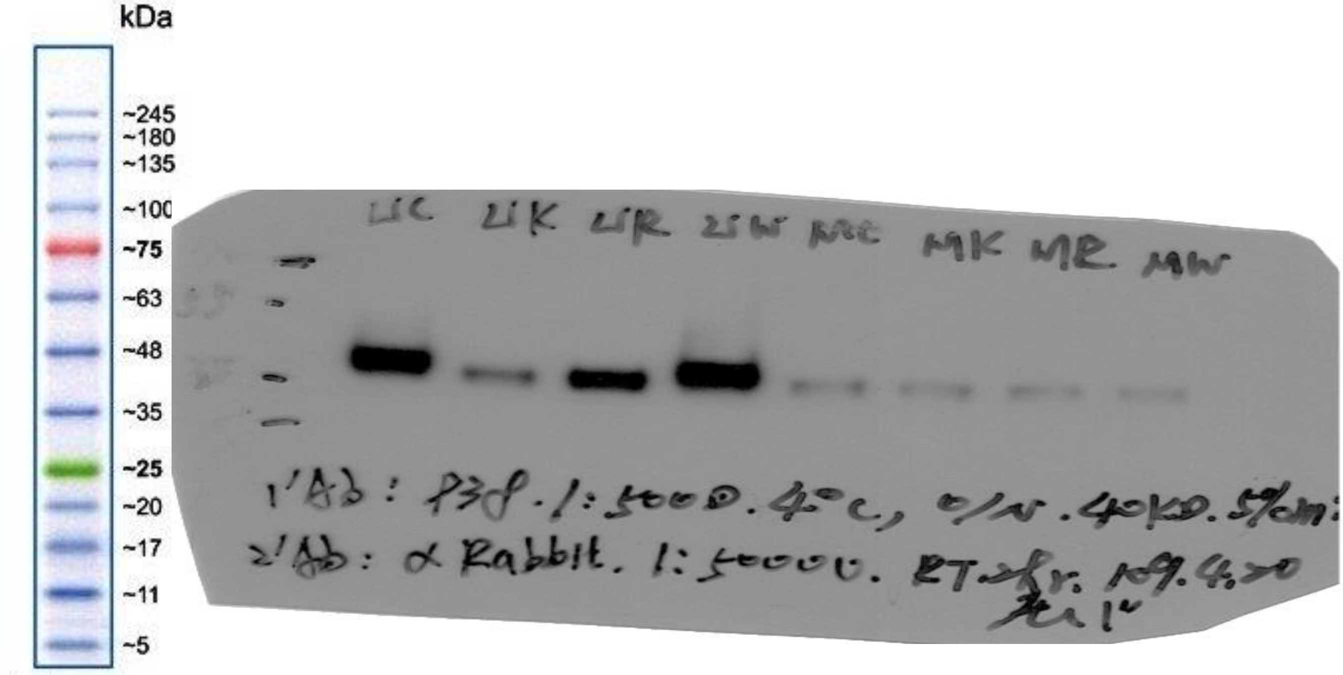

Western blot

Protein: P38

1^0 Ab: Cell Signaling, rabbit monoclonal IgG

Catalog number: no.#8690

Molecular weight (kDa): 38~40 kDa

Working concentration: 1:1000

Gel (%): 8%

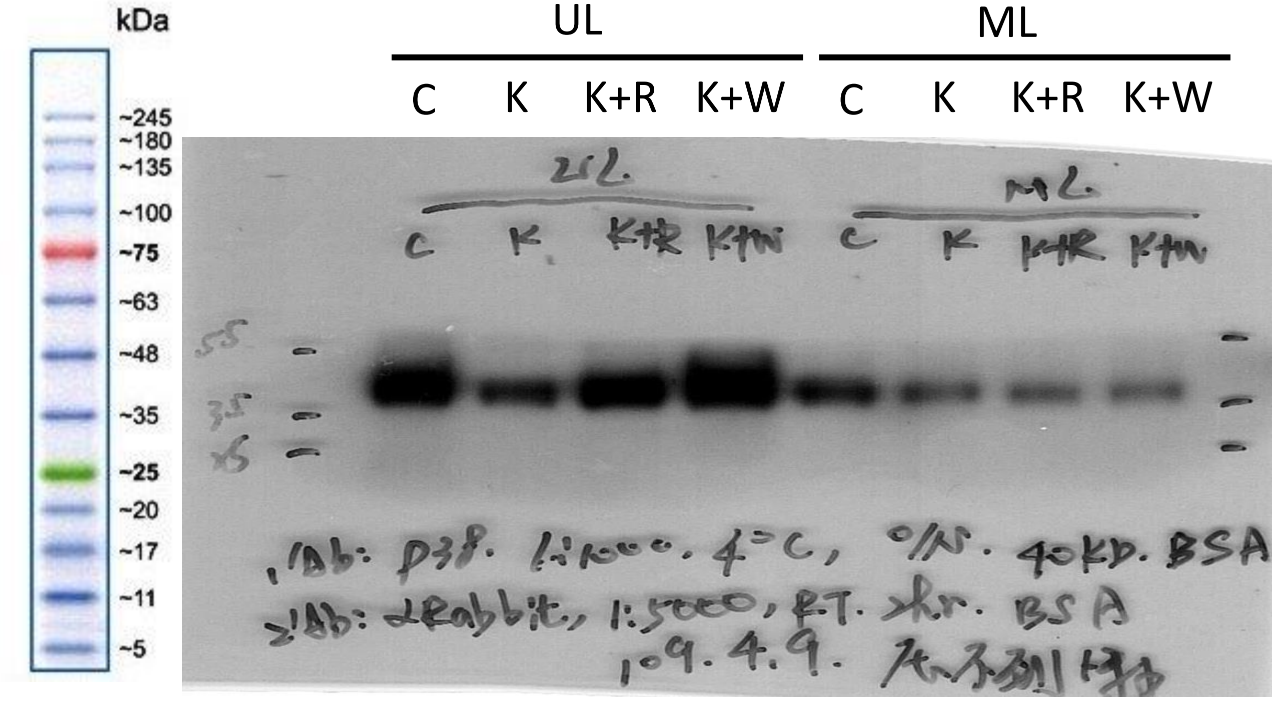

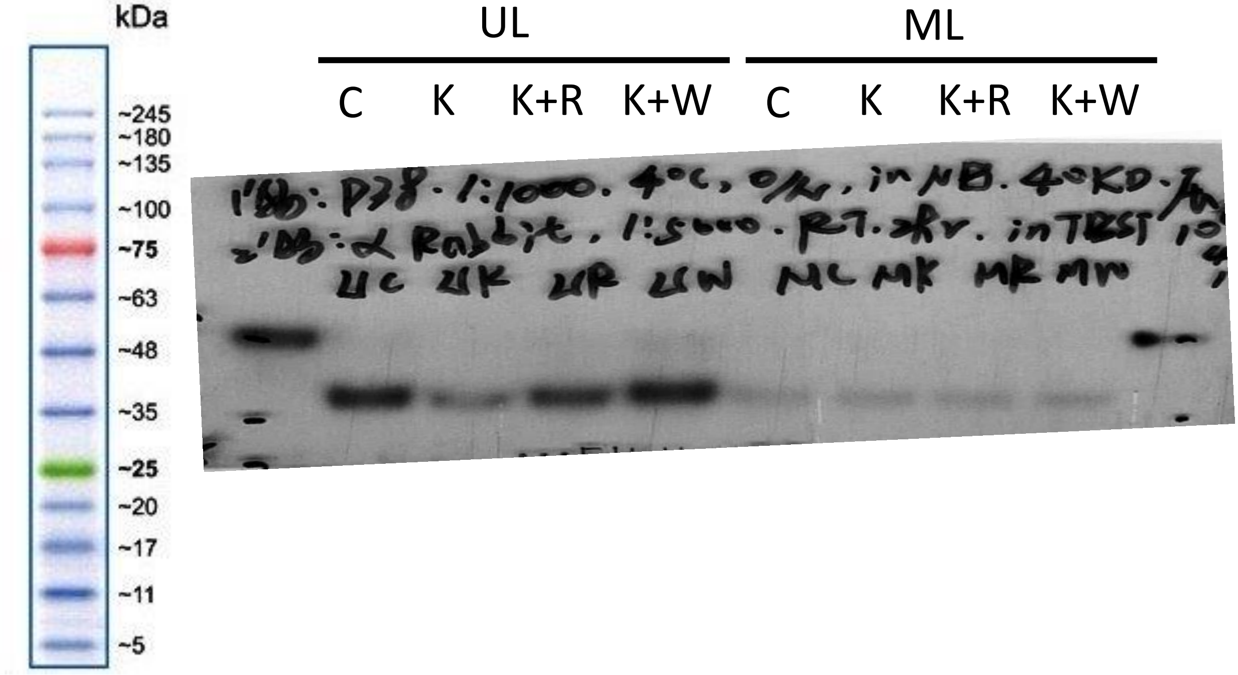

Western blot

Protein: p-P38

1^0 Ab: Cell Signaling, rabbit monoclonal IgG

Catalog number: no.#4511

Molecular weight (kDa): 38~40 kDa

Working concentration: 1:1000

Gel (%): 8%

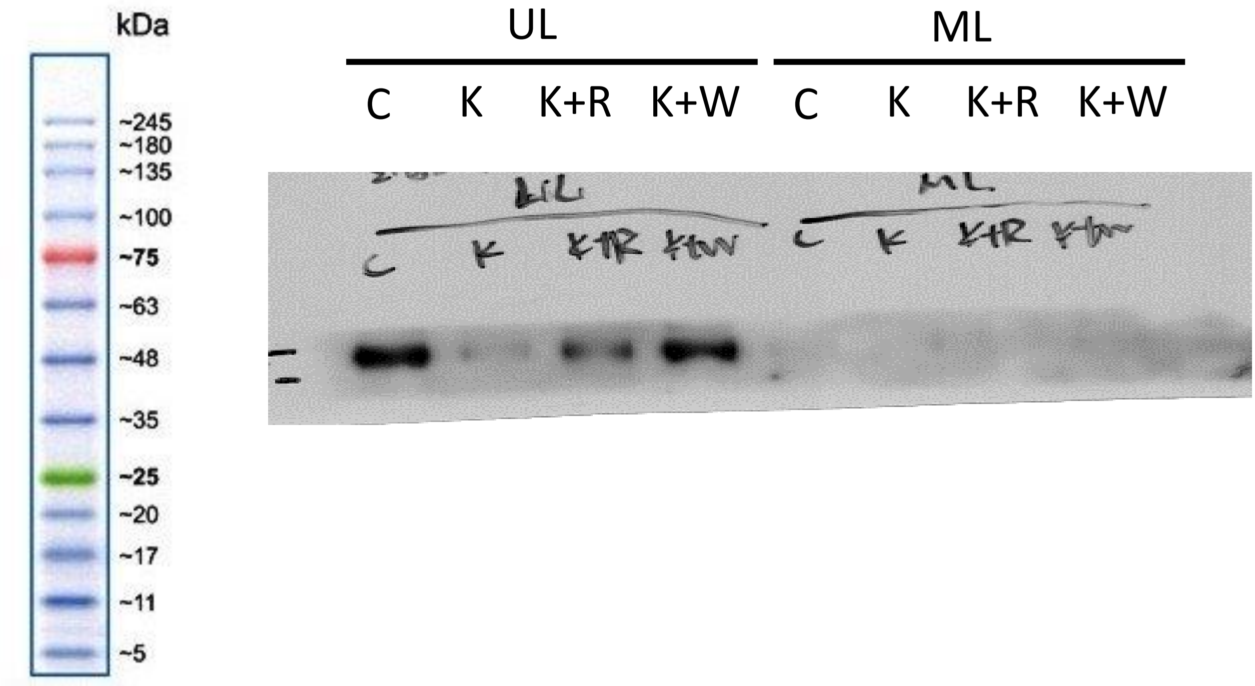

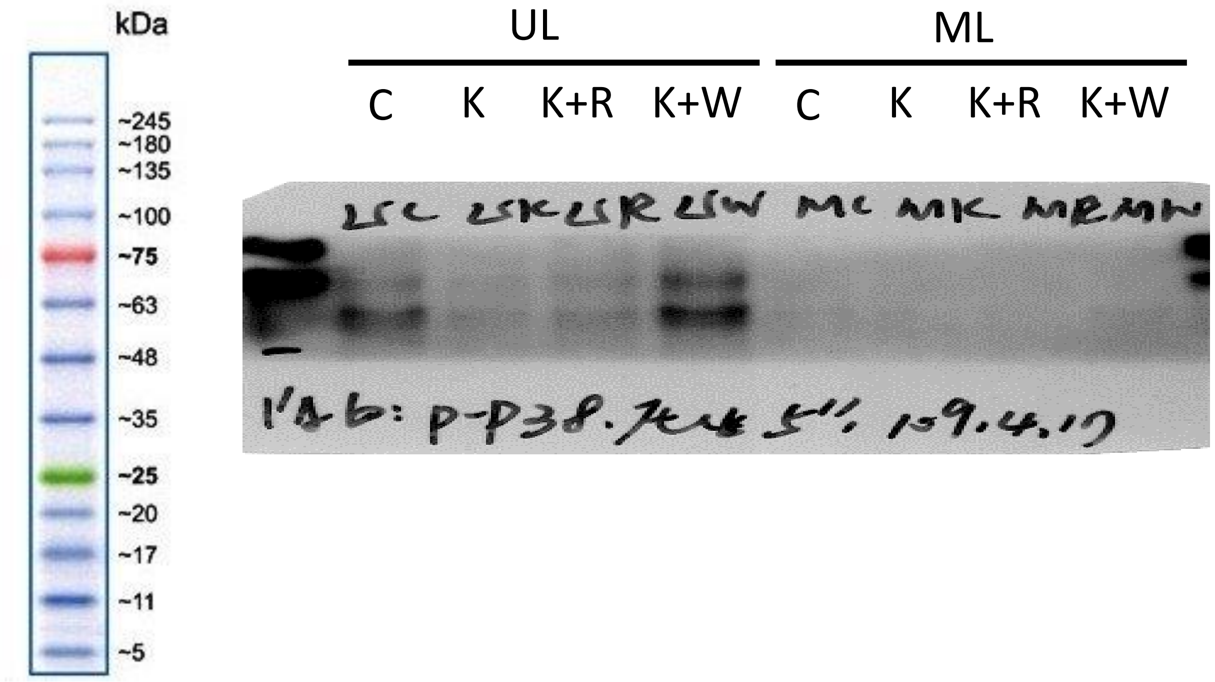

Western blot

Protein: p-P38

1^0 Ab: Cell Signaling, rabbit monoclonal IgG

Catalog number: no.#4511

Molecular weight (kDa): 38~40 kDa

Working concentration: 1:1000

Gel (%): 8%

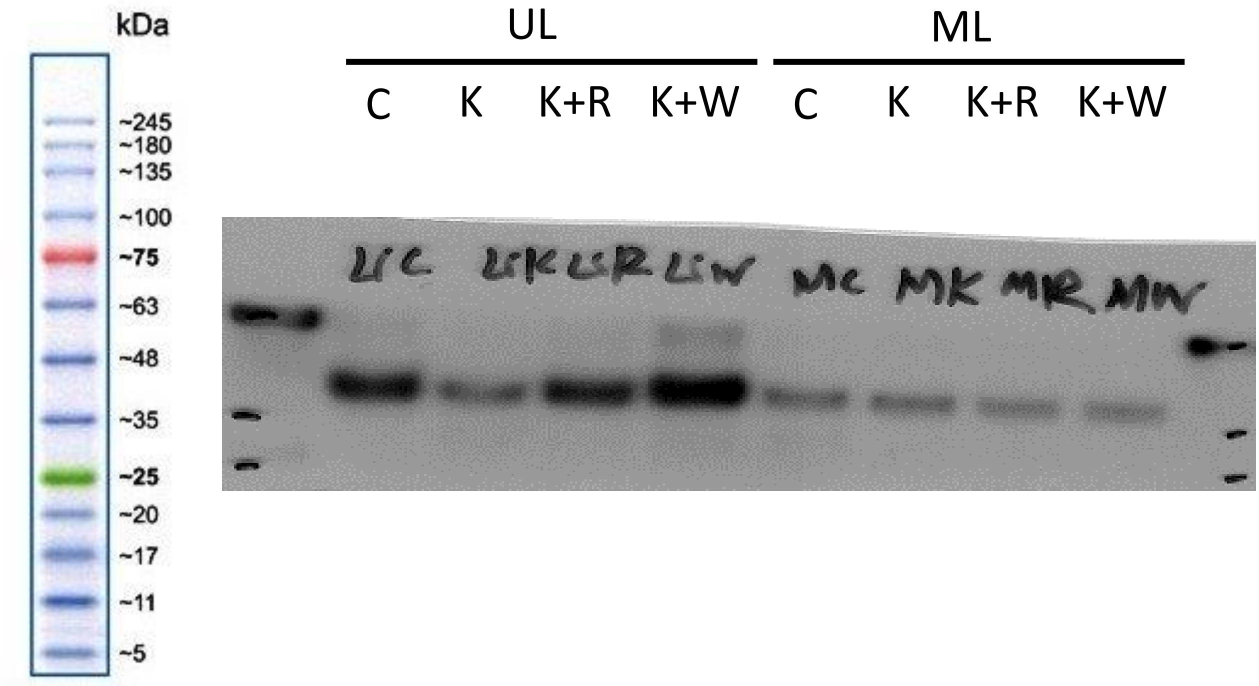

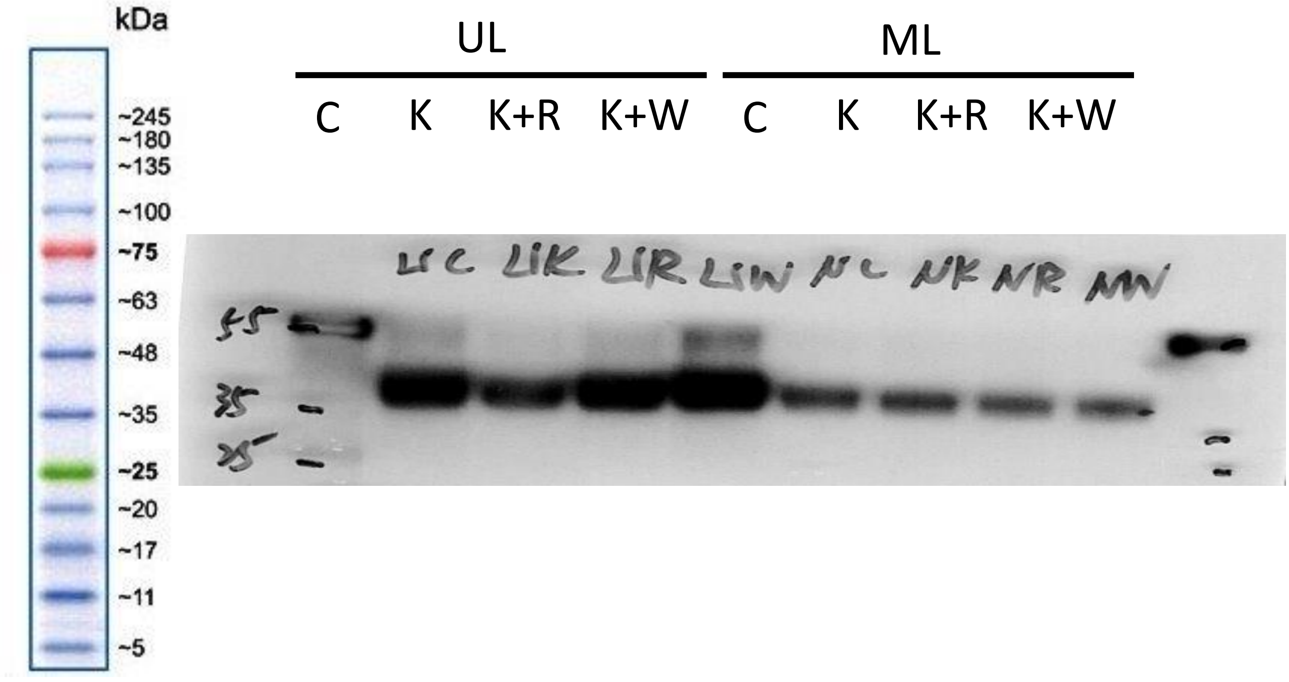

Western blot

Protein: Akt

1^0 Ab: Cell Signaling, mouse monoclonal IgG

Catalog number: no.#2920

Molecular weight (kDa):60 kDa

Working concentration: 1:2000

Gel (%): 8%

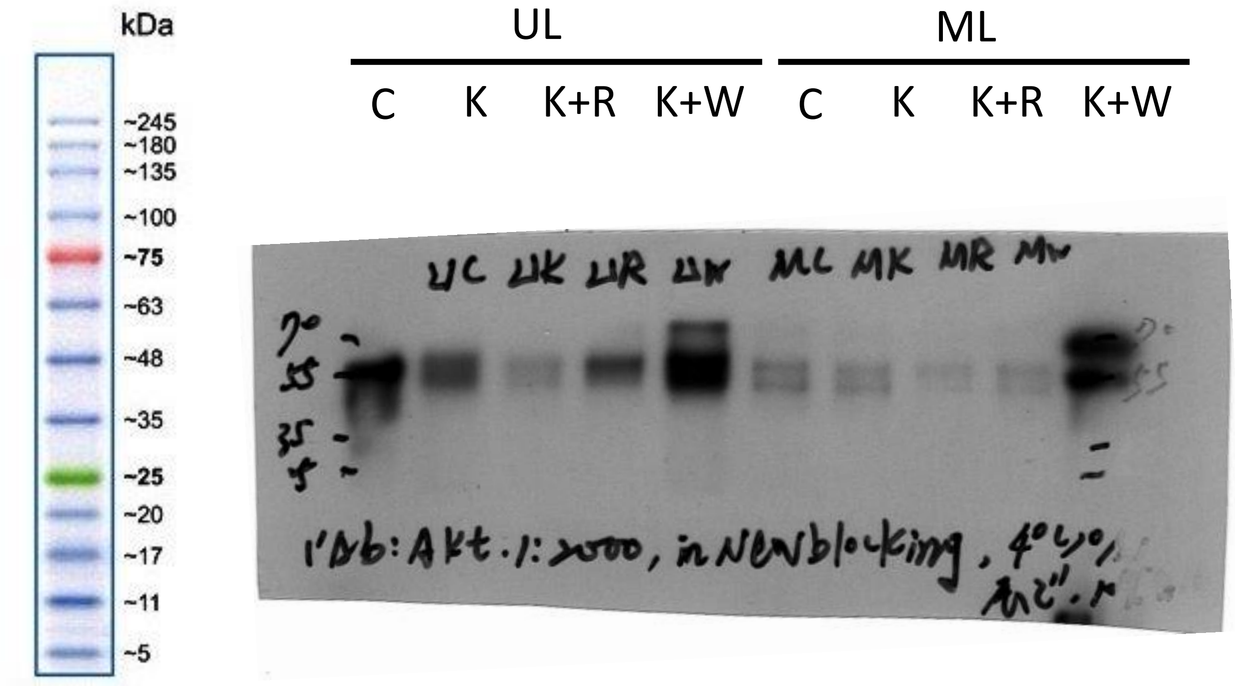

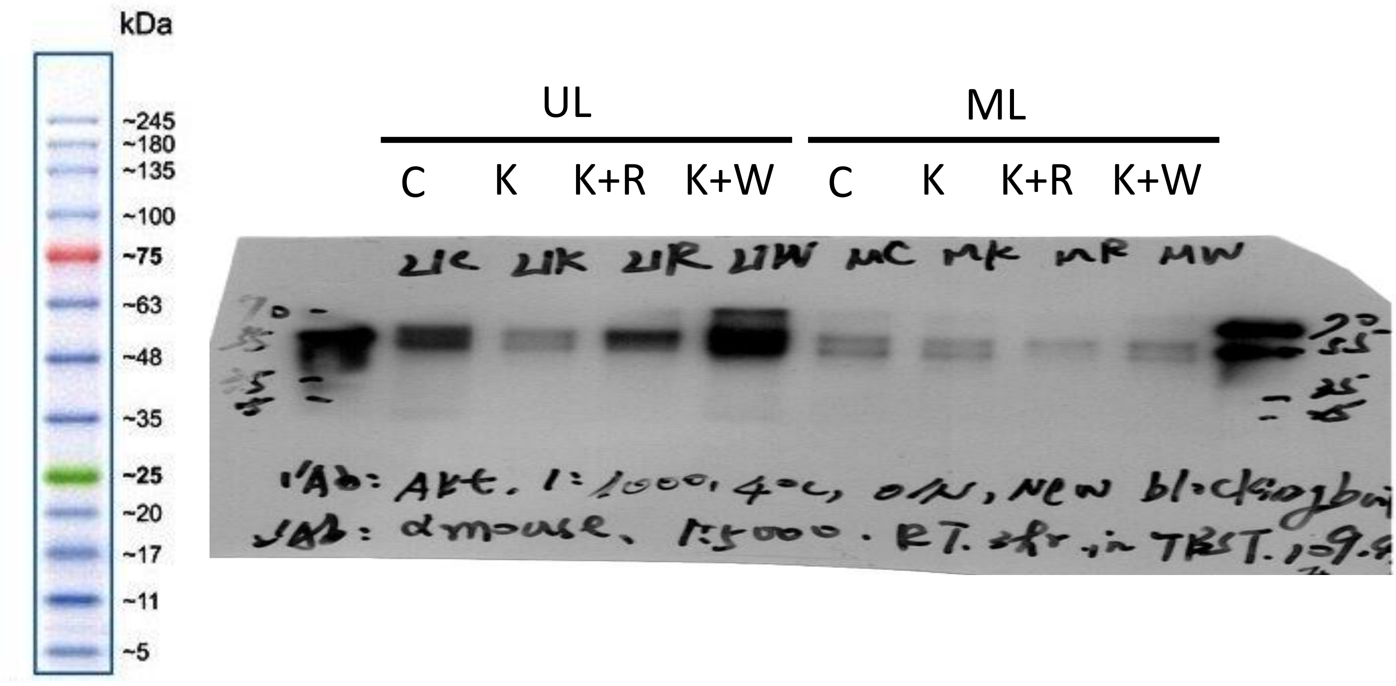

Western blot

Protein: Akt

1^0 Ab: Cell Signaling, mouse monoclonal IgG

Catalog number: no.#2920

Molecular weight (kDa):60 kDa

Working concentration: 1:2000

Gel (%): 8%

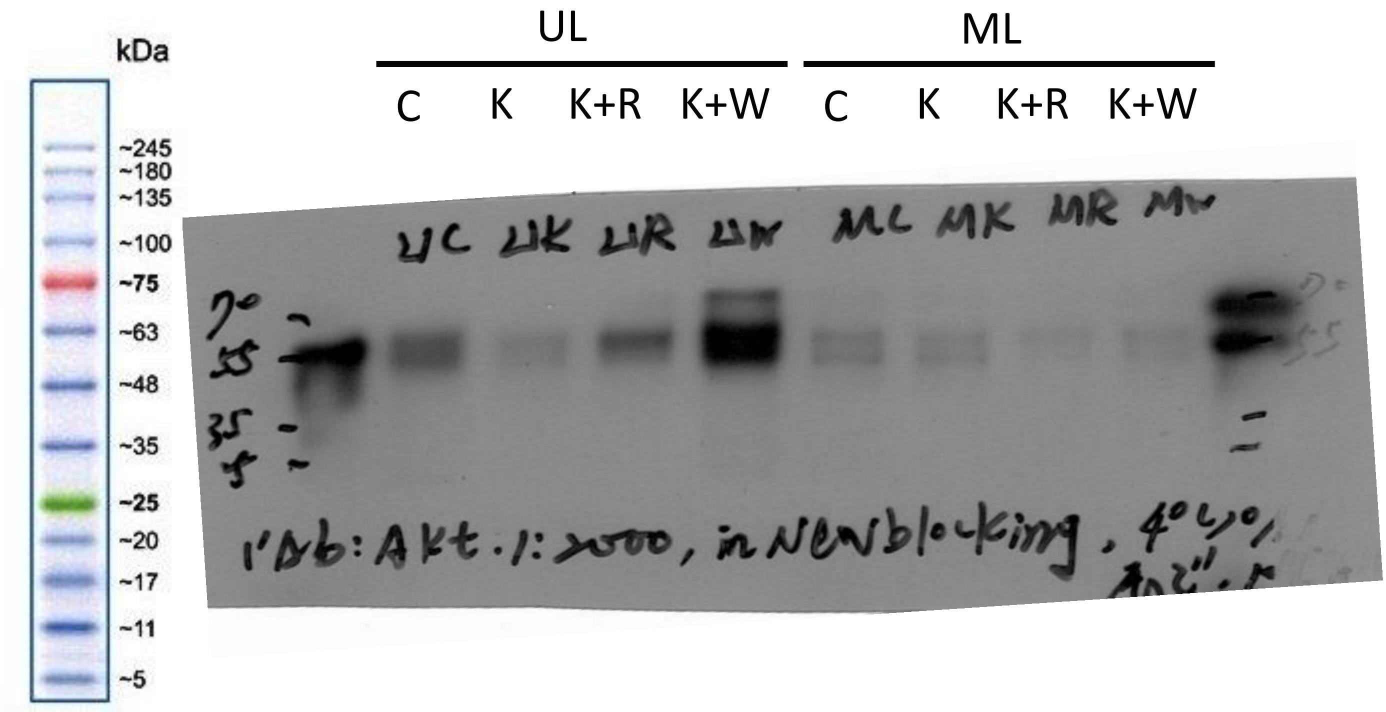

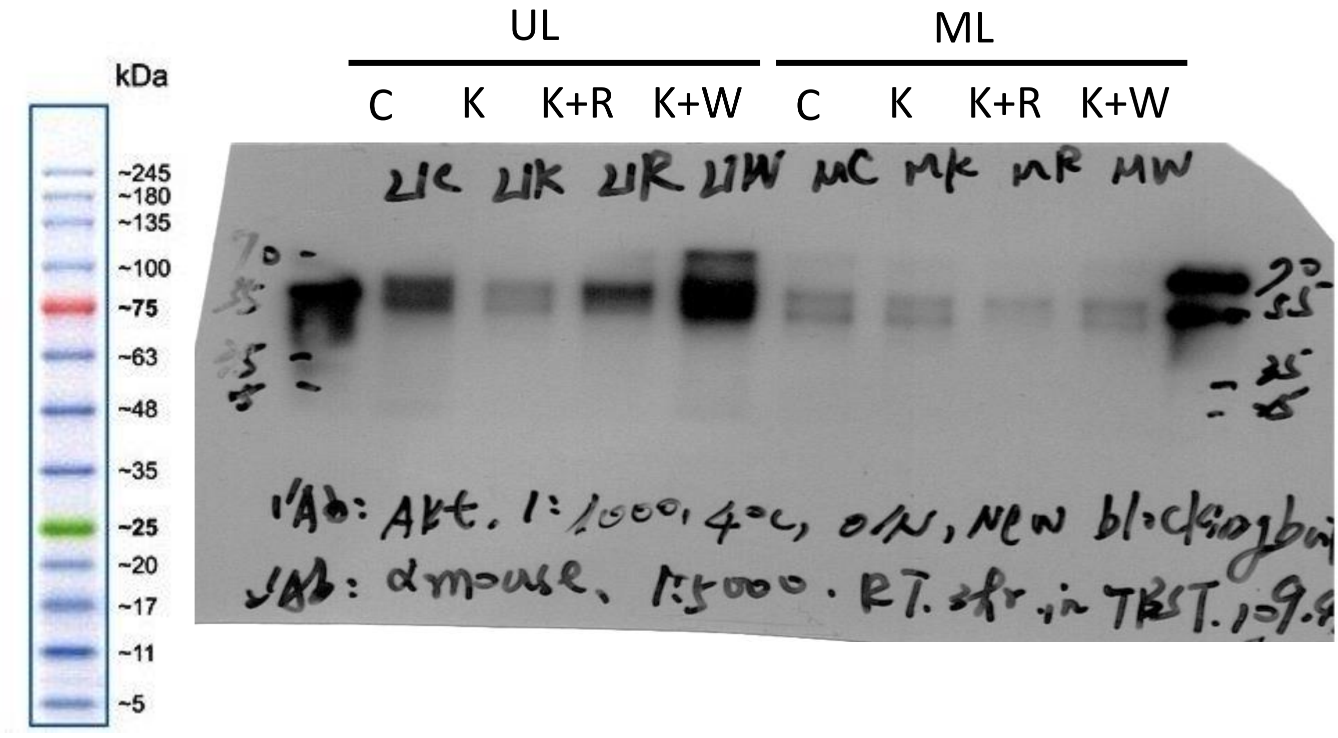

Western blot

Protein: p-Akt

1⁰ Ab: Cell Signaling, rabbit monoclonal IgG

Catalog number: no.#4060

Molecular weight (kDa):60 kDa

Working concentration: 1:1000

Gel (%): 8%

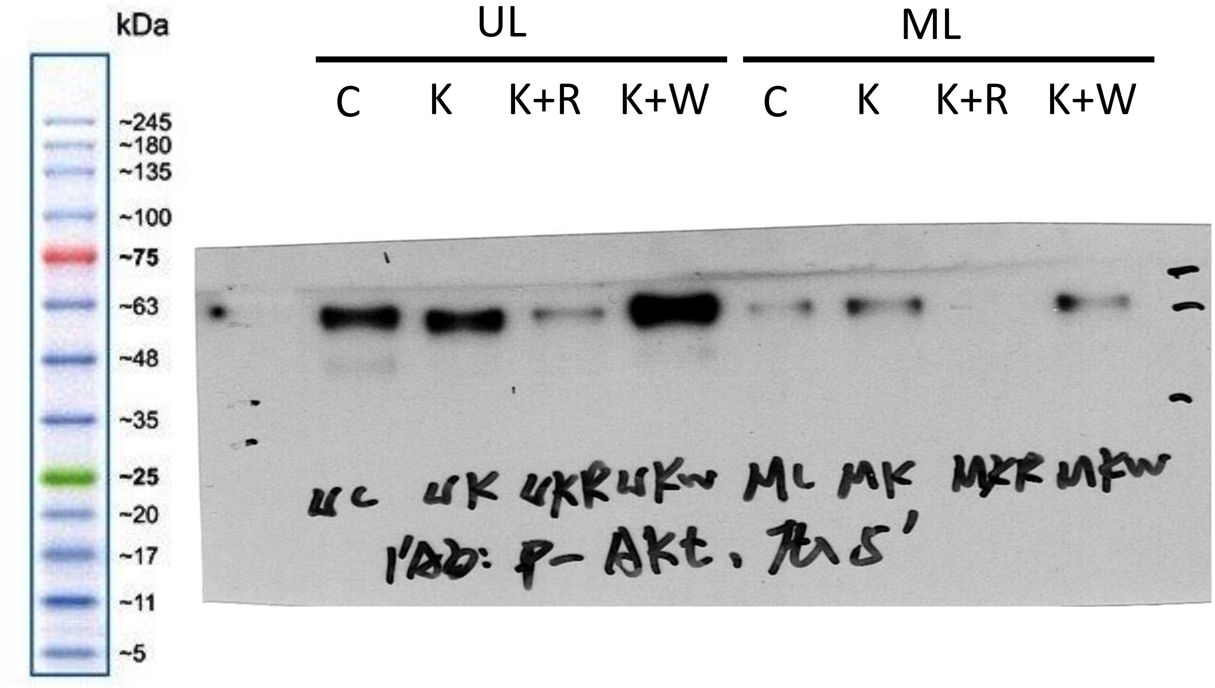

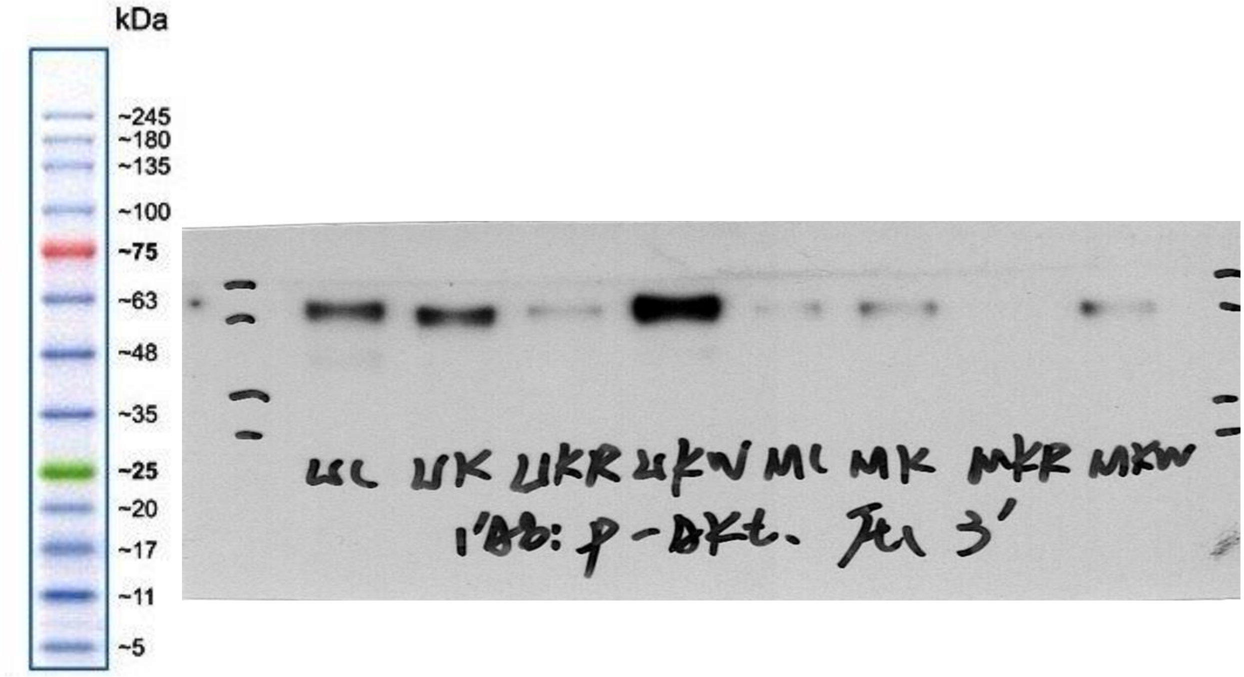

Western blot

Protein: GAPDH

1^0 Ab: Millipore, mouse monoclonal IgG1

Catalog number: no. MAB374

Molecular weight (kDa):36 kDa

Working concentration: 1:1000

Gel (%): 8%

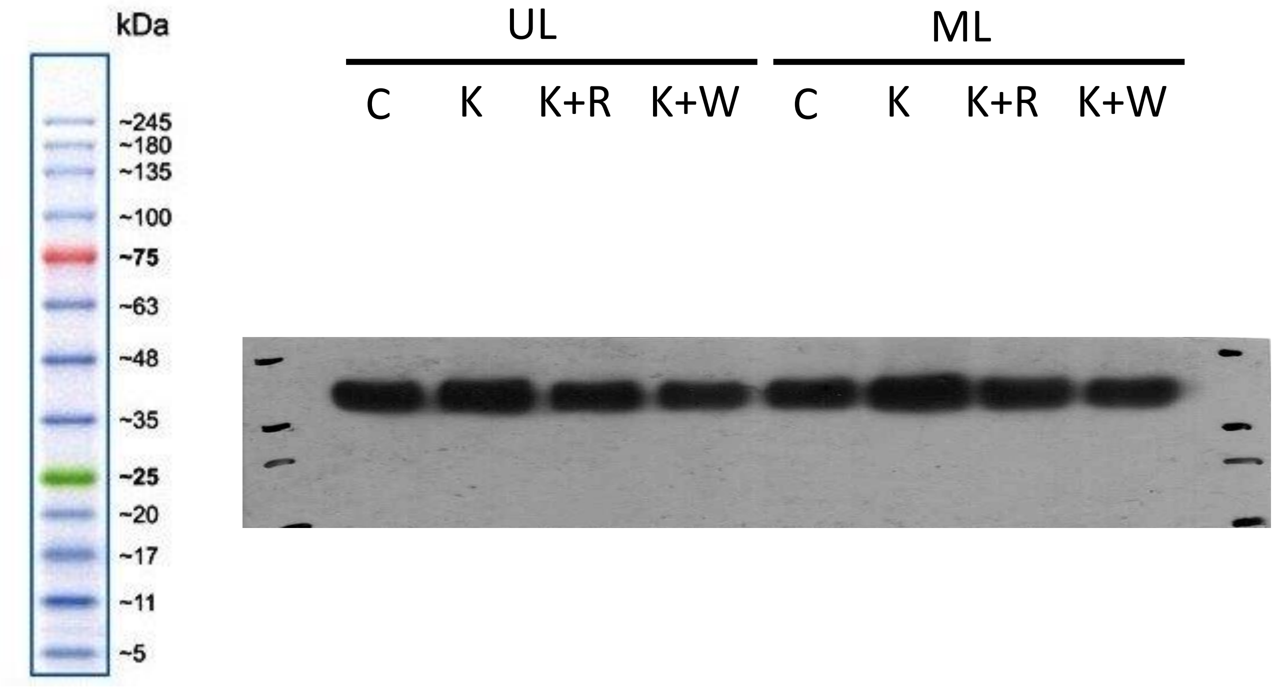

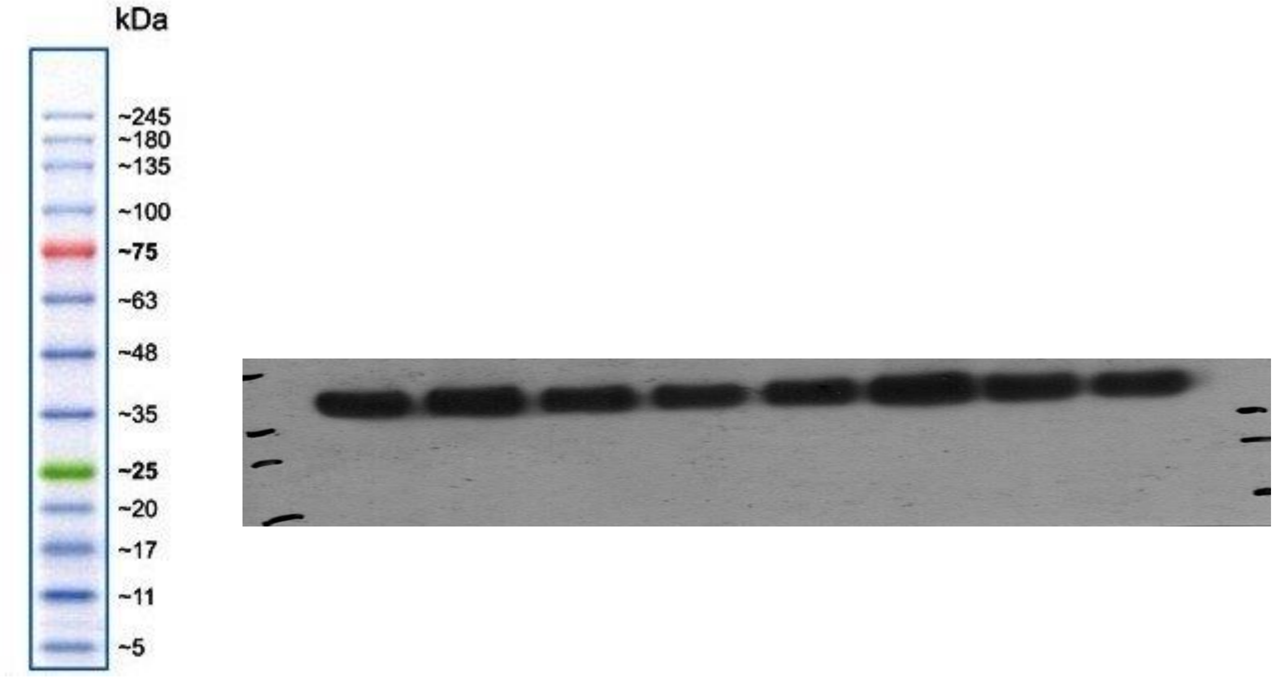

Supplement: Supplementary file 1 [file biology-10-00488-s001.zip › biology-1183477-SI.pdf]
